# Relationship between apathy, glymphatic dysfunction, and white matter integrity in Parkinson’s disease: a multimodal MRI study

**DOI:** 10.3389/fnagi.2026.1939737

**Published:** 2026-10-26

**Authors:** Hao Li, Xuelian Zhao, Wenxia Yang, Jiahao Yan, Zhuo Wang, Siru Kang, Wenzhu Dang, Yang Liu, Jun Wang, Ruiqing Guo, Kai Ai, Guangyao Liu, Jing Zhang

**Affiliations:** 1 The Second Hospital & Clinical Medical School, Lanzhou University, Lanzhou, China; 2 Ganzhou People's Hospital, Ganzhou, China; 3 Department of Magnetic Resonance, The Second Hospital & Clinical Medical School, Lanzhou University, Lanzhou, China; 4 Gansu Province Clinical Research Center for Functional and Molecular Imaging, Lanzhou, China; 5 Department of Clinical and Technical Support, Philips Healthcare, Xi'an, China

**Keywords:** choroid plexus, free-water imaging, glymphatic system, neuroinflammation, Parkinson’s disease apathy, white matter microstructure

## Abstract

**Background:**

Apathy is a disabling non-motor symptom of Parkinson’s disease (PD), but its neuroimaging correlates remain unclear. Glymphatic-related MRI metrics abnormalities and white matter microstructural damage have each been implicated in PD, yet their relationship with apathy is incompletely understood.

**Objective:**

To investigate the relationships among glymphatic-related MRI metrics, white matter abnormalities, and apathy in PD, while accounting for affective, cognitive, motor, and treatment-related factors.

**Methods:**

We prospectively enrolled 41 patients with PD [19 with apathy (APD), 22 without apathy (nAPD)] and 38 healthy controls (HC). Multimodal MRI quantified choroid plexus volume (CPV), perivascular space measures, DTI-ALPS, gBOLD-CSF coupling, free water (FW), free-water-corrected diffusion indices, and NODDI metrics. Tract-based spatial statistics, sparse principal component analysis, partial correlation, sensitivity analyses, and exploratory mediation analyses were performed.

**Results:**

Compared with HC, PD patients showed widespread white matter abnormalities, including increased FW, fwAD, fwMD, fwRD, and ODI and decreased fwFA and NDI. APD patients showed higher FW than nAPD patients in clusters involving the corpus callosum, left superior longitudinal fasciculus, uncinate fasciculus, inferior fronto-occipital fasciculus, and other widespread tracts. Within PD, these FW abnormalities were associated with apathy severity; several associations persisted after adjustment for UPDRS-III and levodopa equivalent daily dose but were attenuated after adjustment for affective or cognitive symptoms. CPV measures were also associated with apathy-related FW abnormalities, with selected associations remaining after adjustment for MoCA or motor/medication measures. Direct associations between individual glymphatic-related MRI metrics and apathy severity were less consistent. Exploratory mediation effects were observed mainly in the combined PD + HC cohort and were not robust within PD or apathy subgroups after multiple-comparison correction.

**Conclusion:**

Apathy in PD was most consistently associated with increased extracellular FW within distributed white matter networks, superimposed on widespread PD-related microstructural damage. Glymphatic-related MRI metrics abnormalities, particularly CPV, were coupled with apathy-related FW but showed weaker direct relationships with apathy severity. These findings support an associative glymphatic–white matter framework for PD apathy, while suggesting partial overlap with broader affective and cognitive dysfunction rather than an apathy-specific causal cascade.

## 1 Introduction

Parkinson’s disease (PD) represents one of the most prevalent progressive neurodegenerative disorders (Morris et al., 2024). Beyond the cardinal motor manifestations—such as bradykinesia, resting tremor, and gait impairment (Tanner and Ostrem, 2024)—the clinical landscape of PD is complicated by a spectrum of non-motor symptoms, including apathy, anxiety, depression, and cognitive impairment (Weintraub and Mamikonyan, 2019; Ahmad et al., 2023). Among these, apathy is a frequent and clinically significant non-motor symptom, typically characterized by a lack of motivation, diminished goal-directed behavior, and reduced responsiveness across behavioral, emotional, and cognitive domains (De Waele et al., 2022; Béreau et al., 2023). Previous studies have indicated that apathy not only exacerbates with disease progression but is also strongly associated with diminished quality of life, impaired social functioning, and increased caregiver burden (Castrioto et al., 2025). Although preliminary evidence suggests the involvement of fronto-striatal and reward-related circuits in the pathogenesis of apathy in PD (Gilmour et al., 2024), the underlying deep pathophysiological mechanisms remain largely elusive. Importantly, apathy in PD frequently coexists with other neuropsychiatric and cognitive manifestations, particularly depression, anxiety, and cognitive impairment (Den Brok et al., 2015; Wen et al., 2016). These symptoms are clinically distinguishable but may share partially overlapping neural substrates involving prefrontal, fronto-striatal, and fronto-limbic circuits (Marafioti et al., 2026). Nevertheless, accumulating evidence indicates that apathy should not simply be regarded as a secondary manifestation of depression or cognitive decline. A meta-analysis showed that approximately half of PD patients with apathy did not have concomitant depression or cognitive impairment (Den Brok et al., 2015), while neuroimaging studies have identified apathy-related alterations that remain detectable after accounting for depressive symptoms or global cognitive status (Marafioti et al., 2026). At the same time, apathy severity may vary with broader disease characteristics, including motor disease burden and dopaminergic state, given the central role of dopaminergic fronto-striatal systems in motivated and reward-guided behavior (Chong and Husain, 2016). Therefore, when investigating the neurobiological correlates of apathy in PD, it is important to consider affective symptoms, cognitive status, motor severity, and dopaminergic medication burden in order to distinguish imaging abnormalities preferentially associated with apathy from those reflecting broader neuropsychiatric or disease-related processes.

In recent years, impairment of brain waste clearance has garnered significant attention in the context of neurodegenerative diseases. The glymphatic system is implicated in the exchange between cerebrospinal fluid (CSF) and interstitial fluid, as well as the clearance of metabolic waste (Naganawa et al., 2024; Iliff et al., 2012; Meng et al., 2024; Ma et al., 2024; Kamagata et al., 2022; Szlufik et al., 2024). Consequently, this fluid transport system presents a promising therapeutic target for the prevention or treatment of PD (Beschorner and Nedergaard, 2024). Given the invasive nature of direct assessment methods, an increasing number of studies have employed non-invasive MRI metrics to characterize glymphatic-related abnormalities from various perspectives. These include Choroid Plexus Volume (CPV), perivascular space (PVS) morphology, Diffusion Tensor Image Analysis along the Perivascular Space (DTI-ALPS), and global low-frequency BOLD-CSF coupling (gBOLD-CSF coupling). However, rather than serving as direct measurements of glymphatic function, these indices act as surrogate MRI markers reflecting CSF production, morphological characteristics of perivascular pathways, directional water diffusion properties, or the coupling between global brain activity and CSF dynamics. Therefore, their biological implications are not entirely identical.

Among these metrics, CPV is posited to be associated with CSF production, the inflammatory status of the choroid plexus, and the burden of brain waste clearance (Iliff et al., 2012; Bitanihirwe et al., 2022; Jeong et al., 2024; Wang et al., 2024). PVS, which maintains connectivity with the subarachnoid space, facilitates the clearance of metabolic waste products, such as α-Synuclein (α-Syn) (Wardlaw et al., 2020; Quadalti et al., 2021). gBOLD-CSF coupling reflects glymphatic function by synchronously measuring low-frequency (<0.1 Hz) BOLD signals in global gray matter and slow CSF flow (Han et al., 2021), and it is increasingly garnering attention for evaluating glymphatic function in PD patients (Chen et al., 2025; Wang et al., 2023). Collectively, these approaches characterize functional alterations in the glymphatic system from multiple perspectives, including CSF production, peri-arterial CSF volumetric spatial characteristics, peri-venous CSF efflux capacity, and metabolic waste clearance. Recently, the utility of the DTI-ALPS index has been subject to debate. Emerging evidence suggests that it reflects local diffusion properties within deep white matter rather than directly characterizing metabolic waste clearance efficiency (Mossige et al., 2026). While debate on this topic persists, the ALPS index remains a sensitive indicator for assessing microstructural integrity and perivascular space status (Qin et al., 2023; Bae et al., 2023a,b).

Beyond glymphatic-related MRI metrics abnormalities, white matter microstructural damage has also been recognized as a significant imaging feature of PD, potentially preceding macroscopic structural alterations such as neuronal loss, axonal degeneration, and demyelination (Bai et al., 2022; Welton et al., 2023). The mechanisms underlying these changes are complex and multifaceted, involving neuroinflammation-mediated cytoskeletal disruption, oxidative stress, and other pathological processes (Cisneros et al., 2022). The Neurite Orientation Dispersion and Density Imaging (NODDI) sequence, based on multi-shell diffusion MRI, characterizes white matter microstructural abnormalities at multiple levels by quantifying various microstructural parameters, including the neurite density index (NDI), orientation dispersion index (ODI), and isotropic volume fraction (fiso) (Kamiya et al., 2020). Existing studies suggest that PD patients exhibit extensive white matter damage associated with cognitive, emotional, and gait impairments (Zhang et al., 2023; Wei et al., 2024; Shen et al., 2022). Apathy, as a symptom characterized by a core lack of motivation, may also involve abnormalities in fronto-striatal and limbic system-related white matter pathways (Gilmour et al., 2024; Lu et al., 2024). On the other hand, the interaction between impaired brain waste clearance, neuroinflammation, oxidative stress, and white matter microstructural damage suggests a potential close link between glymphatic-related MRI metrics abnormalities and white matter microstructural alterations (Cai et al., 2024).

However, existing studies present notable limitations. First, research investigating the association between apathy in PD and glymphatic-related MRI metrics remains largely unexplored. Second, previous studies have predominantly relied on single-modality imaging indices, which precludes a holistic understanding of the complexity inherent in brain waste clearance abnormalities. Finally, there is a paucity of multimodal studies that integrate glymphatic-related MRI metrics with white matter microstructural imaging to further explore their associative patterns with the severity of apathy. To bridge these gaps, this study aims to integrate glymphatic-related MRI metrics—including CPV, PVS, DTI-ALPS, and gBOLD-CSF coupling—with NODDI-derived white matter microstructural parameters. We systematically compared imaging differences among PD patients, healthy controls, and different apathy subtypes, and investigated the relationships between these metrics and apathy severity. Furthermore, mediation analysis was employed to explore the interplay among glymphatic-related MRI metrics abnormalities, white matter microstructural damage, and apathy symptoms, providing a novel perspective for understanding the pathophysiological mechanisms of non-motor symptoms in PD.

## 2 Materials and methods

### 2.1 Participants and clinical data

We prospectively enrolled 41 patients with PD from the Department of Neurology and Neurosurgery at Lanzhou University Second Hospital between March 2023 and February 2025. The inclusion criteria for all PD patients were as follows: (i) fulfillment of the clinical diagnostic criteria for PD; (ii) right-handedness; and (iii) absence of other major neuropsychiatric disorders, history of cerebral hemorrhage, cerebral infarction, traumatic brain injury, or neurosurgery. Concurrently, 38 age and sex matched healthy controls (HC) were recruited. For all subjects (PD and HC), demographic data including age and sex were recorded prior to MRI acquisition. The Apathy Evaluation Scale (AES) was administered under the guidance of a physician. Additionally, Disease duration of all PD patients was recorded as of the date of MRI acquisition. Based on an AES score cutoff of ≥38 (Santangelo et al., 2014; Varanese et al., 2011), the PD cohort was stratified into an apathy group (APD) and a non-apathy group (nAPD). In addition to demographic data (age, sex) and the Apathy Evaluation Scale (AES), all participants (including PD patients and healthy controls) were assessed using the Self-Rating Anxiety Scale (SAS), the Self-Rating Depression Scale (SDS), and the Montreal Cognitive Assessment (MoCA) Beijing version. For PD patients, the Unified Parkinson’s Disease Rating Scale Part III (UPDRS-III) score and Levodopa Equivalent Daily Dose (LEDD) were additionally recorded to evaluate motor severity and dopaminergic medication burden. UPDRS-III and LEDD were not applicable to healthy controls due to the absence of Parkinsonian motor symptoms and dopaminergic treatment. The overall study pipeline is illustrated in Figure 1.

**Figure 1:**
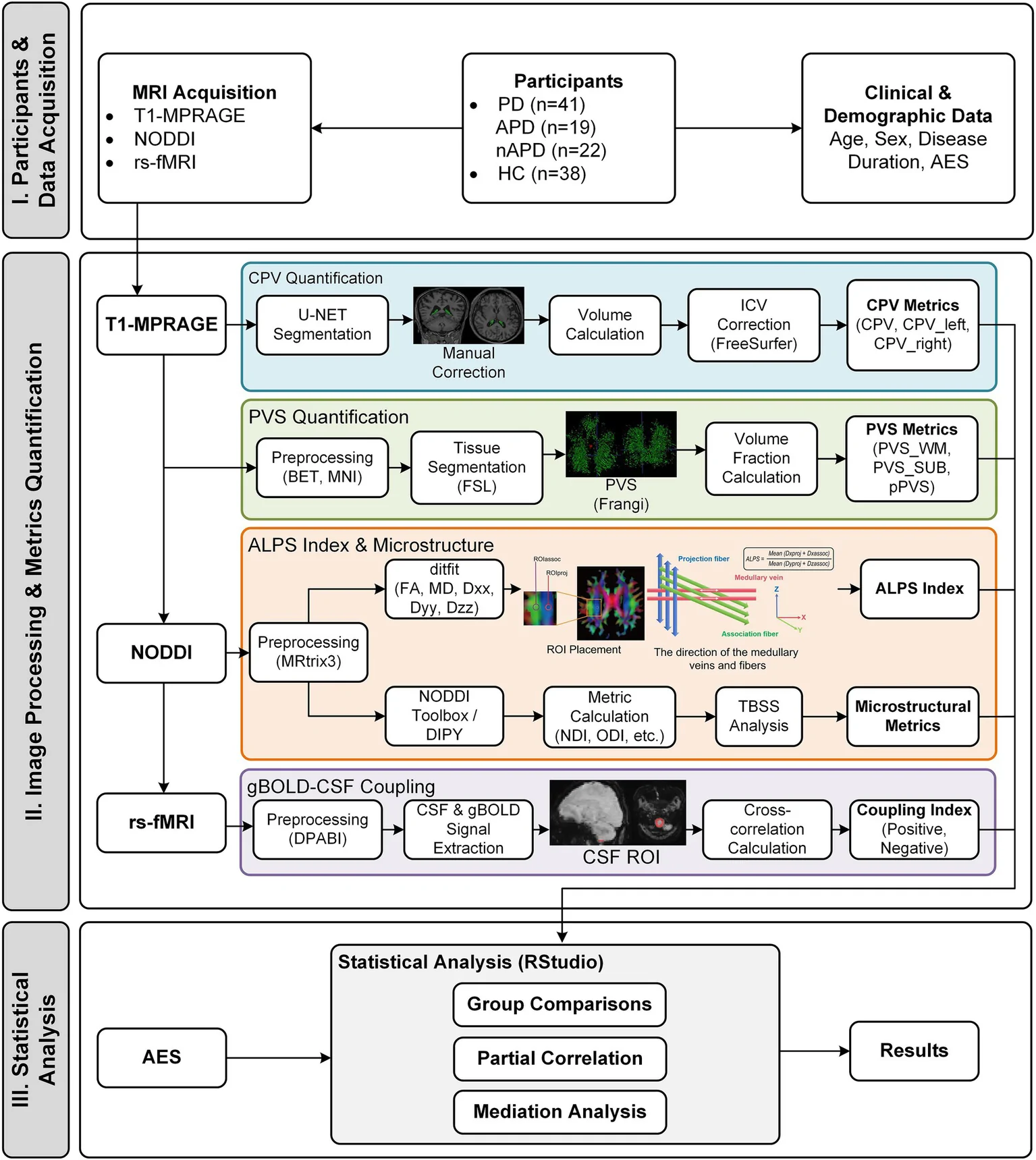
Schematic illustration of the study pipeline. The diagram summarizes the participant groups, MRI data acquisition, image processing steps, metric quantification, and statistical analyses. A total of 79 participants were enrolled, comprising 41 PD Patients were divided into APD and nAPD groups according to an AES score cutoff of ≥38. 19 APD patients, 22 nAPD patients, and 38 HC. Clinical and demographic data included age, sex, disease duration and AES. Three MRI sequences were acquired: T1-MPRAGE for CPV quantification via U-NET segmentation with manual correction and ICV normalization; NODDI for microstructural metrics (neurite density index, orientation dispersion index, free water, etc.) and diffusion tensor imaging-based analysis of the ALPS index; and resting-state fMRI for gBOLD-CSF coupling analysis. Additionally, PVS volume fractions were quantified from T1 images using Frangi filtering in white matter (PVS_WM) and subcortical gray matter (PVS_SUB). Statistical analyses performed in RStudio included group comparisons, partial correlations (controlling for age, sex, and disease duration), and mediation analyses with false discovery rate (FDR) correction. PD, Parkinson’s disease; AES, Apathy Evaluation Scale; APD, apathetic PD; nAPD, non-apathetic PD; HC, healthy controls; ICV, intracranial volume; NODDI, Neurite Orientation Dispersion and Density Imaging; ALPS, index of diffusion along perivascular spaces; gBOLD-CSF coupling, global BOLD–cerebrospinal fluid coupling; TBSS, tract-based spatial statistics; FDR, false discovery rate; CPV, choroid plexus volume; CPV_left, left hemisphere CPV; CPV_right, right hemisphere CPV; PVS, perivascular space volume; PVS_SUB, PVS in subcortical gray matter nuclei; PVS_WM, PVS in the white matter region; pPVS, whole-brain PVS.

### 2.2 MRI acquisition

MRI scans were performed using a Philips Ingenia CX 3.0 T scanner equipped with a 32-channel head coil. To minimize the potential impact of variations in sleep status on the study results, the scanning time was uniformly controlled between 8:00 p.m. and 10:00 p.m. PD patients discontinued medication for more than 12 h prior to MRI data acquisition. To minimize motion artifacts, all participants were placed in the supine position with foam padding inserted into the head coil during the scan. The acquired sequences were as follows: T1-weighted images: Obtained using a sagittal magnetization-prepared rapid gradient-echo (MPRAGE) sequence. Representative imaging parameters were: repetition time (TR) = 6.4 ms, echo time (TE) = 2.9 ms, voxel size = 1.0 × 1.0 × 1.0 mm^3^, and matrix = 256 × 256 × 170. NODDI images: Obtained with the following imaging parameters: TR = 5,000 ms, TE = 102 ms, voxel size = 2.5 × 2.5 × 2.5 mm^3^, and matrix = 96 × 96 × 96. Resting-state BOLD imaging: Performed using a Multiband echo-planar imaging (MB-EPI) sequence. Parameters included: TR = 1,000 ms, TE = 30 ms, voxel size = 3 × 3 × 3 mm^3^, flip angle (FA) = 90°, field of view (FOV) = 256 mm × 256 mm, 240 time points, and a total scan duration of 4 min.

### 2.3 CPV quantification

In this study, a pre-trained deep learning model based on the U-NET architecture (Eisma et al., 2024) was employed to perform automatic and precise segmentation of the choroid plexus (CP) on 3D T1-MPRAGE images. This model, developed based on multi-source MRI datasets, has demonstrated superior performance compared to FreeSurfer and manual segmentation when processing such datasets. To mitigate anatomical variations, all T1 images were spatially aligned to the International Consortium for Brain Mapping-Montreal Neurological Institute 152 standard T1-weighted template (ICBM-MNI152) via nonlinear registration prior to segmentation. Following automatic segmentation, all generated CP masks underwent manual inspection and correction by a senior neuroradiologist using ITK-SNAP software (Version 3.8; http://www.itksnap.org). Finally, we calculated the bilateral choroid plexus volumes (CPV_left, CPV_right) and summed them to obtain the total brain CPV volume (CPV). To correct for individual head size differences, the intracranial volume (ICV) was obtained using the Recon-all command in FreeSurfer (Version 6.0.7.13; http://surfer.nmr.mgh.harvard.edu/). The standardized CPV was calculated as the ratio of raw CPV to ICV (CPV/ICV × 10^3^).

### 2.4 PVS volume fraction calculation

The image preprocessing pipeline began with bias field correction using ANTs (v2.5.4) (Tustison et al., 2010). Subsequently, skull stripping was performed using the Brain Extraction Tool (BET) algorithm in FSL (V6.0.7.13) (Jenkinson et al., 2012). White matter was segmented using FSL-FAST. The resulting white-matter partial-volume map was transformed into MNI space, thresholded at 0.5, and binarized to generate the white-matter mask. Subcortical structures were segmented using FSL-FIRST, and the composite FIRST segmentation was transformed to MNI space and binarized to generate the subcortical mask. The subcortical mask was removed from the white-matter mask to ensure spatially nonoverlapping white-matter and subcortical compartments. The segmentation of PVS itself was accomplished using the multi-scale Frangi filtering function within the Quantitative Imaging Toolkit (QIT, v2.0.1) (Cabeen et al., 2018), with preset parameters referencing previous studies (Sepehrband et al., 2019). PVS burden was quantified relative to normalized brain tissue volume rather than intracranial volume or the corresponding regional mask volume. Normalized brain tissue volume was calculated from the FSL-FAST gray-matter and white-matter partial-volume estimates, with tissue volumes adjusted by the volumetric scaling factor derived from the affine registration matrix:


$$V_{\text{brain}}=V_{\mathrm{GM}}+V_{\mathrm{WM}}$$

The white-matter and subcortical PVS measures were then calculated as


$$\mathrm{PVS}\_\mathrm{WM}(\%)=\frac{V_{\mathrm{PVS},\mathrm{WM}}}{V_{\text{brain}}}\times 100$$

And


$$\mathrm{PVS}\_\mathrm{SUB}(\%)=\frac{V_{\mathrm{PVS},\mathrm{SUB}}}{V_{\text{brain}}}\times 100$$

Total PVS burden was defined as


$$\text{pPVS}(\%)=\mathrm{PVS}\_\mathrm{WM}+\mathrm{PVS}\_\mathrm{SUB}=\frac{V_{\mathrm{PVS},\mathrm{SUB}}+V_{\mathrm{PVS},\mathrm{WM}}}{V_{\text{brain}}}\times 100$$

Thus, PVS_WM and PVS_SUB represent the proportions of normalized brain tissue volume occupied by PVS within the white-matter and subcortical compartments, respectively, whereas pPVS represents the combined PVS burden across both compartments.

### 2.5 DTI-ALPS index calculation

The NODDI data were preprocessed using MRtrix3 (version 3.0.4; http://www.mrtrix.org) (Tournier et al., 2019), including denoising, Gibbs ringing artifact removal, head motion correction, and bias field correction. Subsequently, the dtifit function in FSL (Version 6.0.7.13; https://fsl.fmrib.ox.ac.uk) was used to extract and create metrics such as fractional anisotropy (FA) and mean diffusivity (MD). Then, the *3dTcat*, *3dcalc*, and *3dThreetoRGB* functions in AFNI (version 25.1.03) (Cox, 1996) were employed to obtain diffusivity maps in different directions (Dxx, Dyy, and Dzz) and color-coded FA maps for each subject. Based on the JHU atlas (ICBM label for 2 mm), two spherical regions of interest (ROIs) with a diameter of 5 mm were placed at the level of the body of the lateral ventricle in the projection and association fiber regions on the FA maps (Taoka et al., 2017). The diffusivity values along the x, y, and z directions within each ROI were extracted and recorded as Dxx-proj, Dyy-proj, Dzz-proj, Dxx-assoc, Dyy-assoc, and Dzz-assoc, respectively. The ALPS index was calculated using the following formula:


$$\text{ALPS index}=\frac{\text{mean}(\mathrm{Dxx}-\text{proj},\mathrm{Dxx}-\text{assoc})}{\text{mean}(\mathrm{Dyy}-\text{proj},\mathrm{Dzz}-\text{assoc})}$$

The ALPS index was calculated for each hemisphere (ALPS_right, ALPS_left), and the average of the two was taken as the final ALPS index.

### 2.6 Microstructural damage index calculation

The NODDI data were preprocessed using MRtrix3 (version 3.0.4; http://www.mrtrix.org) (Tournier et al., 2019), including denoising, Gibbs ringing artifact removal, head motion correction, and bias field correction. Subsequently, the dtifit function in FSL (Version 6.0.7.13; https://fsl.fmrib.ox.ac.uk) was used to extract and create metrics such as fractional anisotropy (FA). The NODDI_toolbox_v1.0.5^1^ was then employed on Matlab2019a to calculate the Neurite Density Index (NDI), Orientation Dispersion Index (ODI), and Volume Fraction of Isotropic Diffusion Compartment (fiso). Finally, the Python-based neuroimaging toolbox DIPY^2^ was used to calculate Free Water (FW) and free-water corrected metrics, including free-water corrected FA (fwFA), mean diffusivity (fwMD), axial diffusivity (fwAD), and radial diffusivity (fwRD). Subsequent analysis was performed using Tract-based Spatial Statistics (TBSS). Via FNIRT (FSL’s nonlinear registration tool), the fwFA, fwMD, fwAD, fwRD, NDI, ODI, and fiso images of each participant were registered to the FMRIB58_FA template in Montreal Neurological Institute (MNI) space. By using an FA threshold of 0.2, a mean FA image was calculated from the images of all participants to generate the mean FA skeleton. The registered FA maps of each participant were projected onto the skeleton based on the maximum FA value detected in the search direction. Finally, fwFA, fwMD, fwAD, fwRD, NDI, ODI, and fiso skeletons were created using the projection parameters obtained from the aforementioned steps. Statistical analysis was performed separately for the PD, HC, APD, and nAPD groups, using threshold-free cluster enhancement (TFCE) correction and 5,000 permutation tests. The results were overlaid with the JHU White-Matter Tractography Atlas and the JHU ICBM-DTI-81 White-Matter template to determine anatomical locations in standard space. The *fslmaths* command was used to split clusters with statistical differences, and the *fslmeants* command was used to extract the mean values of the significantly different brain regions for subsequent statistical analysis. No demographic or clinical covariates were included in the voxel-wise TBSS group comparisons. Age, sex, and disease duration were controlled only in the subsequent statistical analyses performed using imaging measures extracted from the significant TBSS clusters.

### 2.7 gBOLD-CSF coupling analysis

fMRI data were processed using DPABI V7.2 (Yan et al., 2016) based on MATLAB software. The first 10 scans were removed to adapt to scanner noise, and the remaining 230 scans were processed through the following four steps: (1) slice timing correction; (2) realignment of head-motion; (3) temporal band-pass filtering (0.01–0.1 Hz); and (4) smoothing using a 4 × 4 × 4 full-width at half maximum (FWHM) kernel. Nuisance regression analysis was not applied to the gBOLD signals, CSF signals, and motion parameters because these variables were the focus of the current study (Wang et al., 2023). A gray matter template was generated on the segmented tissue probability maps of the structural images and transformed into the individual functional image space. Two medical imaging physicians, in a double-blind manner, manually delineated the ROI of the CSF at the edge level between the upper spinal cord and the lower cerebellum using the unsmoothed fMRI images. The BOLD signals of the whole-brain gray matter and CSF were extracted, respectively, and the cross-correlation function between them was calculated to quantify their coupling strength at multiple time-lags (−20s to +20s) based on Pearson correlation. Based on previous studies, we also estimated the cross-correlation function between the negative derivative of the gBOLD signal and the CSF signal (Han et al., 2021). A permutation method with 10,000 iterations was used to obtain the null distribution of coupling, and the statistical significance of coupling was calculated based on this distribution. Finally, the positive and negative coupling peak times and the coupling indices (Positive coupling and Negative coupling) for each subject at the corresponding time points were obtained. Since the coupling index is a negative value, lower values reflect a better association between global brain activity and CSF flow.

### 2.8 Statistical analysis

Statistical analyses were performed using RStudio (Version 4.3.0). Two-sample t-tests and Mann–Whitney U tests were used to compare differences in continuous variables between two groups, while chi-square tests were used for categorical variables. Given the extensive significant results obtained from the TBSS analysis, Sparse Principal Component Analysis (SPCA) based on the average eigenvalue method was used to extract principal components for the PD group (SPCA_PD), incorporating only PD patients based on the TBSS difference results between the PD and HC groups. Because the sign of an SPC and its corresponding loadings is mathematically arbitrary, no *post hoc* sign inversion was applied. Therefore, positive or negative SPC scores were not assigned an intrinsic biological meaning. Components were interpreted according to the absolute magnitude and relative pattern of their loadings, together with the direction of the original TBSS group differences.

Depending on whether the data distribution met normality assumptions, Spearman or Pearson partial correlation analyses were conducted for all subjects, PD, HC, APD, and nAPD groups, regressing out age, sex, and disease duration (for analyses involving HC group data, only age and sex were regressed out due to missing disease duration data). These analyses explored the correlations among glymphatic-related MRI metrics (CPV, CPV_right, CPV_left, pPVS, PVS_WM, PVS_SUB, ALPS, ALPS_right, ALPS_left, Positive coupling, and Negative coupling), NODDI-related metrics (principal components), and AES. Correlations among glymphatic metrics were also explored within each group. To address potential confounding by clinical comorbidities, data on depression (SDS), anxiety (SAS), cognition (MoCA), motor severity (UPDRS-III), and medication burden (LEDD) were included as covariates in sensitivity analyses. In analyses involving the entire cohort (PD + HC) or the HC group alone, age and sex were included as core covariates. In analyses restricted to PD patients, including the overall PD cohort and the APD and nAPD subgroups, age, sex, and disease duration were included as core covariates. To evaluate the potential influence of overlapping clinical dimensions, three sensitivity models were constructed on the basis of the corresponding core model. Model A additionally adjusted for anxiety and depressive symptoms (SAS and SDS), and Model B additionally adjusted for cognitive performance (MoCA); these two models were applicable to the entire cohort, PD, HC, APD, and nAPD analyses. Model C additionally adjusted for motor severity and dopaminergic medication burden (UPDRS-III and LEDD) and was therefore restricted to analyses involving PD participants, including the overall PD cohort and the APD and nAPD subgroups, because UPDRS-III and LEDD were not applicable to healthy controls. A fully adjusted model was further constructed when sample size and multicollinearity permitted. For analyses of the entire cohort or HC group, the fully adjusted model included age, sex, SAS, SDS, and MoCA. For analyses of the overall PD cohort, the fully adjusted model included age, sex, disease duration, SAS, SDS, MoCA, UPDRS-III, and LEDD. Given the limited sample sizes of the APD and nAPD subgroups, the separate sensitivity models were prioritized over the fully adjusted model to reduce the risk of overfitting. To avoid multicollinearity, the Variance Inflation Factor (VIF) was calculated for all covariates in the partial correlation models involving conventional clinical covariates. A maximum VIF ≥ 5 indicated significant collinearity, in which case the collinear covariates were split into separate models rather than being included simultaneously. It should be noted that VIF diagnostics were applied exclusively to the covariate sets within each model. For partial correlation analyses involving SPCA-derived principal components and APD/nAPD TBSS-derived FW clusters, these metrics served as the core independent variables rather than covariates. Therefore, VIF calculation was not performed on these independent variables themselves. Furthermore, since the base covariates (e.g., age, sex, disease duration) in these exploratory analyses were identical to those in the primary analysis, which already passed VIF diagnostics (max VIF < 5), no repeated VIF reporting was necessary. For multiple comparison correction, a hierarchical FDR strategy was applied to preserve the statistical power of the primary findings. Specifically, the primary analysis (Pool 1: Core model), sensitivity analyses (Pool 2: Models A/B/C), and exploratory comorbidity correlation analyses (Pool 3) were corrected for multiple comparisons using the Benjamini–Hochberg method in independent hypothesis pools, thereby avoiding excessive penalty on the primary results by exploratory analyses.

Finally, due to the limited sample size of the PD subgroups (APD = 19, nAPD = 22), mediation analyses were retained as exploratory only, controlling solely for age, sex, and disease duration to avoid model overfitting. Mediation analysis based on multiple regression was performed for the PD, APD, and nAPD groups. Glymphatic-related MRI metrics and NODDI-related metrics (principal components) were selected as independent and mediator variables, respectively, with neuropsychiatric scores (clinical symptom severity) as the outcome variable. All models controlled for age, sex, and disease duration. A non-parametric percentile bootstrap method with 5,000 resamples was used to generate 95% confidence intervals (CIs) for indirect, direct, and total effects. *p*-values for indirect effects were calculated from the bootstrap distribution (two-tailed test). To avoid false positives due to multiple comparisons, FDR correction (Benjamini-Hochberg method) was applied to the *p*-values of indirect, direct, and total effects for all mediation models. Corrected *p* < 0.05 was considered statistically significant.

To delineate the full clinical spectrum from healthy aging to late-stage apathy in Parkinson’s disease, exploratory mediation analyses were additionally conducted in the age-matched entire cohort (PD + HC). Within this framework, SPCA was performed on white matter microstructural alterations based on the entire cohort to extract principal components for the full sample (SPCA_All). Given the inherent inter-group mean differences in glymphatic function and white matter microstructural metrics between HC and PD patients, statistical associations in the full-sample analysis may be partially driven by disease status differences. Therefore, the full-sample results are cautiously interpreted as supplementary descriptive evidence supporting the “biological continuum of apathy” hypothesis, rather than as confirmatory primary evidence validating apathy-specific mechanisms within PD.

## 3 Results

### 3.1 Demographic characteristics

A total of 41 patients with PD (56.1% male) and 38 healthy controls (HC) (52.6% male) were included. No significant differences in age or sex were found between groups. The median disease duration in the PD group was 6.00 (3.00–9.00) years. Compared with HC, PD patients exhibited more severe apathy (AES: 36.00 (30.00–42.00) vs. 24.00 (23.00–28.00), *p_FDR_* < 0.001; Figure 2A) and significant differences in SAS, SDS, and MoCA (SAS: 40.00 (35.00–43.00) vs. 28.50 (26.00–35.75); SDS: 46.00 (37.00–50.00) vs. 28.00 (25.25–35.50); MoCA: 22.00 (17.00–25.00) vs. 25.00 (24.00–26.00); all *p_FDR_* < 0.001) (see Table 1).

**Figure 2:**
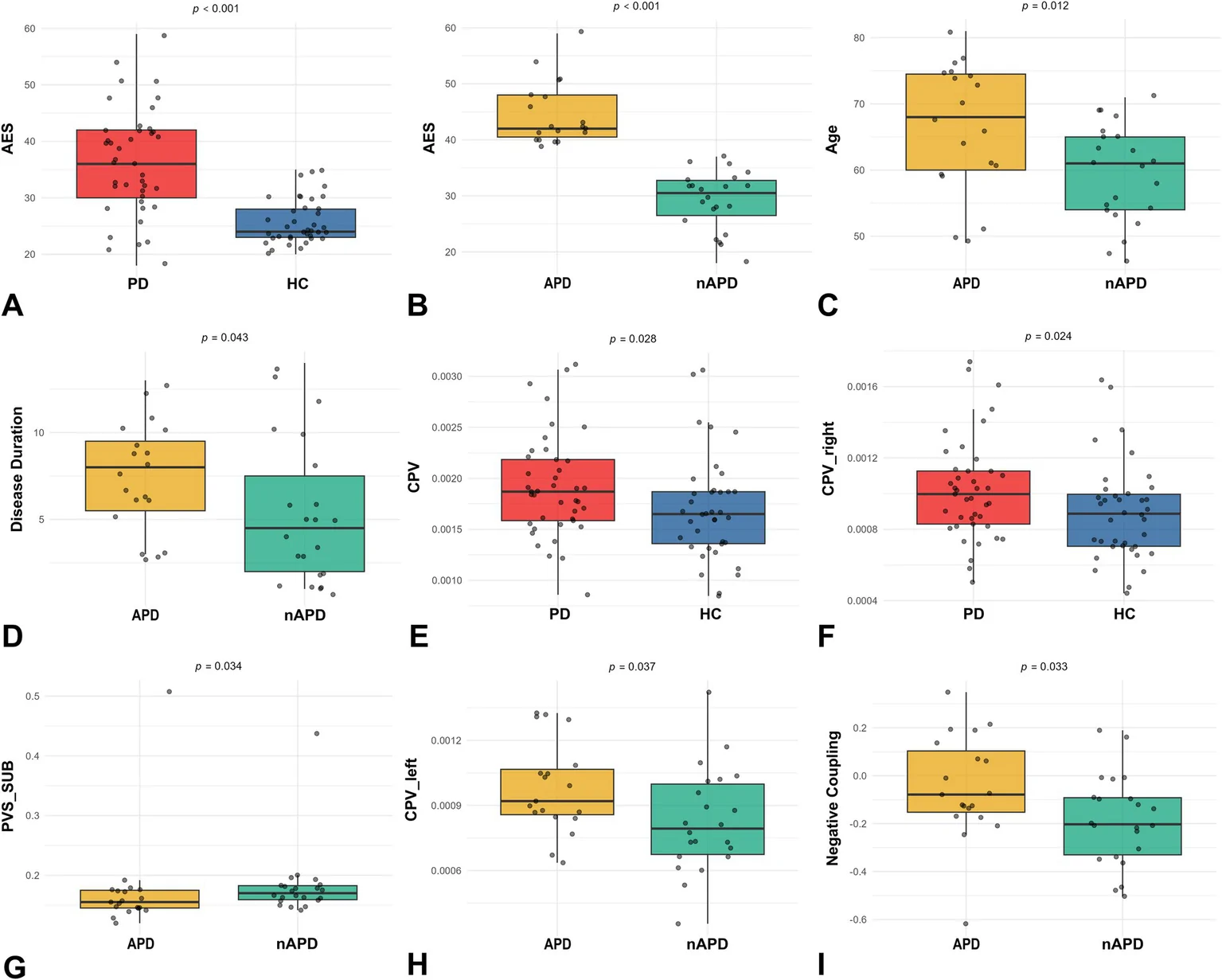
Significant differences in clinical and imaging measures between PD patients and HC, as well as between APD and nAPD patients. **(A)** PD patients exhibited significantly higher apathy scores than HC (AES: 36.00 (30.00–42.00) vs. 24.00 (23.00–28.00), *pFDR* < 0.001). **(B)** After FDR correction, the APD group had significantly higher AES scores than the nAPD group (42.00 (40.50–48.00) vs. 30.50 (26.50–32.75), *p_FDR_* < 0.001). **(C,D)** Before FDR correction, APD patients were older, had longer disease duration, and exhibited more severe anxiety and depressive symptoms than nAPD patients (age: 66.47 ± 9.78 vs. 59.36 ± 7.44, *p* = 0.012; disease duration: 8.00 (5.50–9.50) vs. 4.50 (2.00–7.50), *p* = 0.043). **(E,F)** Before FDR correction, PD patients showed larger bilateral and right CPV compared with HC (PD vs. HC; CPV: 1.87 (1.59–2.18) vs. 1.65 (1.36–1.87), *p* = 0.028; CPV_right: 1.00 (0.83–1.13) vs. 0.89 (0.71–1.00), *p* = 0.024). **(G–I)** Before FDR correction, APD patients had smaller PVS_SUB, larger left CPV, and higher negative coupling compared with nAPD patients (APD vs. nAPD; PVS_SUB: 0.16 (0.15–0.17) vs. 0.17 (0.16–0.18), *p* = 0.034; CPV_left: 0.98 ± 0.21 vs. 0.83 ± 0.24, *p* = 0.037; negative coupling: −0.05 ± 0.22 vs. –0.19 ± 0.19, *p* = 0.033). Data are presented as mean ± SD or median (Q1–Q3), as appropriate according to the distribution of the data. All *p*-values are two-sided; FDR correction was applied for multiple comparisons where indicated. PD, Parkinson’s disease; HC, healthy controls; APD, apathetic PD; nAPD, non-apathetic PD; FDR, false discovery rate; AES, Apathy Evaluation Scale; CPV, choroid plexus volume; CPV_left, left hemisphere CPV; CPV_right, right hemisphere CPV; PVS_SUB, PVS in subcortical gray matter nuclei.

**Table 1:** Demographic, clinical, and MRI characteristics of PD and HC participants.

Characteristic	PD (*n* = 41)	HC (*n* = 38)	*p*
Age (years)	63.00 (55.00–69.00)	60.50 (56.00–65.00)	0.841
Gender (male/female)	23/18	20/18	0.988
Disease duration (years)	6.00 (3.00–9.00)	–	–
AES	36.00 (30.00–42.00)	24.00 (23.00–28.00)	<0.001
SAS	40.00 (35.00–43.00)	28.50 (26.00–35.75)	<0.001
SDS	46.00 (37.00–50.00)	28.00 (25.25–35.50)	<0.001
MoCA	22.00 (17.00–25.00)	25.00 (24.00–26.00)	<0.001
UPDRS-III	47.90 ± 27.56	–	–
LEDD	411.10 ± 264.70	–	–
CPV (‰)	1.87 (1.59–2.18)	1.65 (1.36–1.87)	0.028*
CPV_right (‰)	1.00 (0.83–1.13)	0.89 (0.71–1.00)	0.024*
CPV_left (‰)	0.900 ± 0.236	0.802 ± 0.271	0.250
pPVS (%)	0.43 (0.39–0.46)	0.42 (0.39–0.45)	0.988
PVS_WM (%)	0.26 (0.23–0.28)	0.26 (0.23–0.28)	0.988
PVS_SUB (%)	0.16 (0.15–0.18)	0.17 (0.15–0.18)	0.988
ALPS	1.50 ± 0.21	1.53 ± 0.21	0.748
ALPS_right	1.48 ± 0.21	1.52 ± 0.23	0.748
ALPS_left	1.51 ± 0.25	1.55 ± 0.23	0.748
Negative coupling	−0.12 ± 0.21	−0.07 ± 0.20	0.523
Positive coupling	0.13 ± 0.30	0.11 ± 0.24	0.988

Data are presented as mean ± standard deviation (SD) or median (Q1–Q3) for continuous variables, according to the Shapiro–Wilk test. If both groups were normally distributed, mean ± SD was used. If normality was not satisfied in at least one group, median (Q1–Q3) was presented for both groups. * indicates *p*-values before FDR correction. All tests were two-sided, and *p* < 0.05 was considered statistically significant.

AES, Apathy Evaluation Scale; SAS, Self-Rating Anxiety Scale; SDS, Self-Rating Depression Scale; MoCA, Montreal Cognitive Assessment (Beijing version); UPDRS-III, Unified Parkinson’s Disease Rating Scale Part III; LEDD, Levodopa Equivalent Daily Dose; PD, Parkinson’s disease; HC, healthy controls; CPV, choroid plexus volume; CPV_left, left hemisphere CPV; CPV_right, right hemisphere CPV; PVS, perivascular space volume; PVS_SUB, PVS in subcortical gray matter nuclei; PVS_WM, PVS in the white matter region; pPVS, whole-brain PVS; ALPS, index of diffusion along perivascular spaces; ALPS_left, left hemisphere ALPS; ALPS_right, right hemisphere ALPS.

Among all PD patients, 19 were classified into the apathy group (APD) (AES ≥ 38) and 22 into the non-apathy group (nAPD) (AES < 38). As shown in Table 2, there was no significant difference in sex between the two groups. After FDR correction, the APD group had significantly higher AES scores than the nAPD group (42.00 (40.50–48.00) vs. 30.50 (26.50–32.75), *p_FDR_* < 0.001; Figure 2B). Before FDR correction, the APD group had significantly higher SAS and SDS scores, and was older with a longer disease duration than the nAPD group (SAS: 43.32 ± 8.42 vs. 36.95 ± 6.04, *p* = 0.008; SDS: 46.47 ± 8.17 vs. 40.59 ± 9.36, *p* = 0.040; Age: 66.47 ± 9.78 vs. 59.36 ± 7.44, *p* = 0.012; Disease duration: 8.00 (5.50–9.50) vs. 4.50 (2.00–7.50), *p* = 0.043; Figures 2C,D).

**Table 2:** Demographic, clinical, and MRI characteristics of APD and nAPD participants.

Characteristic	APD (*n* = 19)	nAPD (*n* = 22)	*p*
Age (years)	66.47 ± 9.78	59.36 ± 7.44	0.012*
Gender (male/female)	13/6	10/12	0.298
Disease duration (years)	8.00 (5.50–9.50)	4.50 (2.00–7.50)	0.043*
AES	42.00 (40.50–48.00)	30.50 (26.50–32.75)	<0.001
SAS	43.32 ± 8.42	36.95 ± 6.04	0.008*
SDS	46.47 ± 8.17	40.59 ± 9.36	0.040*
MoCA	20.00 (14.00–23.50)	22.00 (21.00–25.75)	0.196
UPDRS-III	44.68 ± 31.82	50.68 ± 23.70	0.901
LEDD	416.74 ± 276.04	406.23 ± 260.96	0.618
CPV (‰)	2.07 ± 0.49	1.80 ± 0.49	0.133
CPV_right (‰)	1.08 ± 0.30	0.97 ± 0.27	0.254
CPV_left (‰)	0.98 ± 0.21	0.83 ± 0.24	0.037*
pPVS (%)	0.40 (0.37–0.44)	0.43 (0.40–0.47)	0.210
PVS_WM (%)	0.24 ± 0.04	0.26 ± 0.03	0.133
PVS_SUB (%)	0.16 (0.15–0.17)	0.17 (0.16–0.18)	0.034*
ALPS	1.51 ± 0.19	1.48 ± 0.23	0.685
ALPS_right	1.50 ± 0.22	1.46 ± 0.20	0.646
ALPS_left	1.53 ± 0.18	1.50 ± 0.30	0.770
Negative coupling	−0.05 ± 0.22	−0.19 ± 0.19	0.033*
Positive coupling	0.04 (−0.12–0.21)	0.25 (0.15–0.31)	0.109

Data are presented as mean ± standard deviation (SD) or median (Q1–Q3) for continuous variables, according to the Shapiro–Wilk test. If both groups were normally distributed, mean ± SD was used. If normality was not satisfied in at least one group, median (Q1–Q3) was presented for both groups. * indicates *p*-values before FDR correction. All tests were two-sided, and *p* < 0.05 was considered statistically significant.

APD, apathetic PD; nAPD, non-apathetic PD; AES, Apathy Evaluation Scale; SAS, Self-Rating Anxiety Scale; SDS, Self-Rating Depression Scale; MoCA, Montreal Cognitive Assessment (Beijing version); UPDRS-III, Unified Parkinson’s Disease Rating Scale Part III; LEDD, Levodopa Equivalent Daily Dose; CPV, choroid plexus volume; CPV_left, left hemisphere CPV; CPV_right, right hemisphere CPV; PVS, perivascular space volume; PVS_SUB, PVS in subcortical gray matter nuclei; PVS_WM, PVS in the white matter region; pPVS, whole-brain PVS; ALPS, index of diffusion along perivascular spaces; ALPS_left, left hemisphere ALPS; ALPS_right, right hemisphere ALPS.

### 3.2 Alterations in glymphatic-related MRI metrics

Before FDR correction, compared with the HC group, PD patients exhibited larger bilateral and right CPV (PD vs. HC; CPV‰: 1.87 (1.59–2.18) vs. 1.65 (1.36–1.87), *p* = 0.028; CPV_right‰: 1.00 (0.83–1.13) vs. 0.89 (0.71–1.00), *p* = 0.024) (Figures 2E,F). The mean gBOLD-CSF cross-correlation function in the HC group showed a positive peak at a -5 s lag (*p* < 0.001) and a negative peak at a + 8 s lag (*p* < 0.001). In the PD group, the mean gBOLD-CSF cross-correlation function showed a positive peak at a -5 s lag (*p* < 0.001) and a negative peak at a + 11 s lag (*p* < 0.001).

Compared with the nAPD group, and before FDR correction, the APD group exhibited significantly smaller PVS_SUB, larger left CPV volume, and higher Negative coupling (APD vs. nAPD; PVS_SUB: 0.16 (0.15–0.17) vs. 0.17 (0.16–0.18), *p* = 0.034; CPV_left‰: 0.98 ± 0.21 vs. 0.83 ± 0.24, *p* = 0.037; Negative coupling: −0.05 ± 0.22 vs. -0.19 ± 0.19, *p* = 0.033) (Figures 2G–I). The mean gBOLD-CSF cross-correlation function in the APD group showed a positive peak at a -4 s lag (*p* < 0.001) and a negative peak at a + 3 s lag (*p* < 0.001). In the nAPD group, the mean gBOLD-CSF cross-correlation function showed a positive peak at a -6 s lag (*p* < 0.001) and a negative peak at a + 10s lag (*p* < 0.001).

### 3.3 Microstructural damage

In the TBSS analysis comparing the PD and HC groups, PD patients showed increased fwAD, fwMD, fwRD, FW, and ODI, and decreased fwFA and NDI compared to the HC group. After 5,000 permutation tests, we identified 8 significant fwFA clusters, mainly located in the right superior longitudinal fasciculus, forceps minor, and forceps major (Supplementary Table S1); 4 significant fwAD clusters, involving the bilateral superior longitudinal fasciculus, inferior fronto-occipital fasciculus, all parts of the corpus callosum, and anterior thalamic radiation (Supplementary Table S2); 10 significant fwMD clusters, covering the bilateral corona radiata, cingulum, and widespread association fiber regions (Supplementary Table S3); 10 significant fwRD clusters, mainly observed in the right corticospinal tract, bilateral corona radiata, splenium of the corpus callosum, and inferior fronto-occipital fasciculus (Supplementary Table S4); 6 significant FW clusters showing bilateral widespread distribution, affecting the corpus callosum, corona radiata, and centrum semiovale (Supplementary Table S5); 1 significant NDI cluster (42,071 voxels) covering all parts of the corpus callosum, internal capsule, corona radiata, and bilateral superior longitudinal fasciculus (Supplementary Table S6); and 5 significant ODI clusters limited to the right centrum semiovale, inferior fronto-occipital fasciculus, and inferior longitudinal fasciculus (Supplementary Table S7), while no significant differences were found in fiso (Table 3). To ensure the scientific validity of subsequent mediation analyses, two SPCA analyses were performed. First, based on all subjects (PD + HC), SPCA analysis yielded 5 Sparse Principal Components (SPCs) with a mean sparsity of 54.3% and a total variance explanation rate of 53.94% (Table 4; Supplementary Table S8): SPC1_All was centered on increased fwRD, primarily reflecting myelin integrity damage in the right hemisphere (corticospinal tract, corona radiata) and left frontal lobe, with highly concentrated loadings (fwRD cluster 10 = 0.862), making it the primary source of single structural variation; SPC2_All was characterized by increased fwAD, focusing on projection fibers such as the bilateral anterior thalamic radiation and corticospinal tract; SPC3_All was dominated by increased ODI, concentrated in fiber dispersion in the right hemisphere, accompanied by relative preservation of fwFA (positive loadings); SPC4_All showed an antagonistic relationship between fwFA and fwMD (positive vs. negative), involving the right superior longitudinal fasciculus and body of the corpus callosum; and SPC5_All had the highest variance explanation rate (16.87%), centered on increased FW, accompanied by abnormalities in multiple metrics such as fwMD, fwRD, and ODI. In the SPCA analysis targeting PD patients, 3 SPCs were obtained with a mean sparsity of 57.1% and a total variance explanation rate of 67.54% (Table 5; Supplementary Table S9): SPC1_PD (Figure 3A) was dominated by increased fwMD (fwMD cluster 1 = 0.415, cluster 5 = 0.374, cluster 8 = 0.327, cluster 2 = 0.321), accompanied by fwRD contribution (cluster 10 = 0.328), involving extensive white matter regions such as the right centrum semiovale/corona radiata, left occipitotemporal lobe, and bilateral corpus callosum, explaining the largest variance (31.55%) and serving as the main source of white matter variation in PD patients; SPC2_PD (Figure 3B) was characterized by an antagonistic relationship between decreased fwFA and increased fwRD (fwFA cluster 7 = −0.675, cluster 6 = −0.277, cluster 8 = −0.188 vs. fwRD cluster 3 = 0.419, cluster 5 = 0.339), involving regions such as the right frontal lobe, forceps minor/anterior thalamic radiation, left temporal lobe, inferior fronto-occipital fasciculus, and uncinate fasciculus; and SPC3_PD (Figure 3C) showed its largest absolute loading for FW cluster 6 (loading = −0.647), with additional contributions from fwMD (cluster 3 = −0.442), ODI (cluster 1 = −0.377), fwRD (cluster 8 = −0.291), and fwAD (cluster 4 = −0.252), covering extensive bilateral white matter, corpus callosum, and right occipital association fibers.

**Table 3:** Summary of TBSS differences between the PD and HC groups, group differences were assessed using 5,000 permutation tests with threshold-free cluster enhancement (TFCE) correction; TFCE-corrected *p* < 0.05 was considered statistically significant.

Metric	Direction	Number of clusters	Max cluster (voxels)	Primary affected regions
fwFA	HC > PD	8	3,152	Right SLF, Forceps minor/major, CST
fwAD	PD > HC	4	17,895	Bilateral SLF, IFOF, Corpus callosum, ATR
fwMD	PD > HC	10	9,811	Bilateral corona radiata, Cingulum, Association fibers
fwRD	PD > HC	10	12,910	Right CST, Bilateral corona radiata, Splenium, IFOF
FW	PD > HC	6	17,807	Extensive bilateral WM, Corpus callosum, Corona radiata
NDI	HC > PD	1	42,071	Whole-brain extensive WM (single large cluster)
ODI	PD > HC	5	3,531	Right centrum semiovale, IFOF, ILF
fiso	—	0	0	No significant difference

fwFA, free water-corrected fractional anisotropy; fwAD, free water-corrected axial diffusivity; fwMD, free water-corrected mean diffusivity; fwRD, free water-corrected radial diffusivity; FW, free water; NDI, neurite density index; ODI, orientation dispersion index; fiso, isotropic volume fraction; PD, Parkinson’s disease; HC, healthy controls; SLF, Superior longitudinal fasciculus; IFOF, Inferior fronto-occipital fasciculus; ILF, Inferior longitudinal fasciculus; CST, Corticospinal tract; ATR, Anterior thalamic radiation; WM, White matter.

**Table 4:** Summary of SPCA results of TBSS differences and corresponding brain regions in the whole cohort (PD + HC).

Component	Eigenvalue	Variance (%)	Cumulative (%)	Primary metrics	Key loadings	Brain regions
SPC1_All	4.321	12.34	12.34	fwRD↑ (6/7)	fwRDclu10 0.862	Right CST/corona radiata, Left ATR
SPC2_All	3.558	10.16	22.51	fwAD↑ (4/7)	fwADclu3–0.588, clu1–0.546, clu2–0.509	Bilateral ATR, Brainstem projection fibers
SPC3_All	3.357	9.59	32.10	ODI↑ (4/9) fwFA↓	ODIclu5–0.470, clu1–0.463, clu2–0.405	Right centrum semiovale, Occipitotemporal fibers
SPC4_All	1.740	4.97	37.07	fwFA↑ fwMD↓	fwFAclu4 0.679, fwMDclu1–0.349, clu5–0.329	Right SLF, Body of corpus callosum
SPC5_All	5.904	16.87	53.94	FW↑ (2/16) fwMD↑	FWclu5–0.485, clu6–0.480, fwMDclu3–0.441	Extensive bilateral WM, Right SLF

Total variance explained = 53.94%, average sparsity = 54.3%, *n* = 79 (PD + HC).

clu, cluster; fwRD, free water-corrected radial diffusivity; fwAD, free water-corrected axial diffusivity; ODI, orientation dispersion index; fwFA, free water-corrected fractional anisotropy; fwMD, free water-corrected mean diffusivity; FW, free water; CST, corticospinal tract; ATR, anterior thalamic radiation; SLF, superior longitudinal fasciculus; WM, white matter.

**Table 5:** Summary of SPCA results of TBSS differences and corresponding brain regions in the PD group.

Component	Eigenvalue	Variance (%)	Cumulative (%)	Primary metrics	Key loadings	Brain regions
SPC1_PD	8.833	31.55	31.55	fwMD↑ (8/12) fwRD↑ (3/12)	fwMDclu1 0.415, clu5 0.374, fwRDclu10 0.328, fwMDclu8 0.327, fwMDclu2 0.321	Right centrum semiovale/corona radiata, left occipitotemporal, bilateral corpus callosum, right frontal deep
SPC2_PD	5.876	20.99	52.53	fwFA↓ (3/12) fwRD↑ (4/12)	fwFAclu7–0.675, fwRDclu3 0.419, fwRDclu5 0.339, fwFAclu6–0.277, fwRDclu1 0.231	Right frontal/forceps minor/ATR, left temporal/IFOF/uncinate fasciculus
SPC3_PD	4.204	15.01	67.54	FW↑ (1/12) fwMD↑ (1/12) ODI↑ (1/12) fwRD↑ (1/12) fwAD↑ (1/12)	FWclu6–0.647, fwMDclu3–0.442, ODIclu1–0.377, fwRDclu8–0.291, fwADclu4–0.252	Extensive bilateral WM/corpus callosum/corona radiata, right occipital association fibers

Total variance explained = 67.54%, average sparsity = 57.1%, *n* = 41 (PD only).

clu, cluster; fwMD, free water-corrected mean diffusivity; fwRD, free water-corrected radial diffusivity; fwFA, free water-corrected fractional anisotropy; FW, free water; ODI, orientation dispersion index; fwAD, free water-corrected axial diffusivity; NDI, neurite density index; CST, corticospinal tract; IFOF, inferior fronto-occipital fasciculus; ATR, anterior thalamic radiation; WM, white matter.

**Figure 3:**
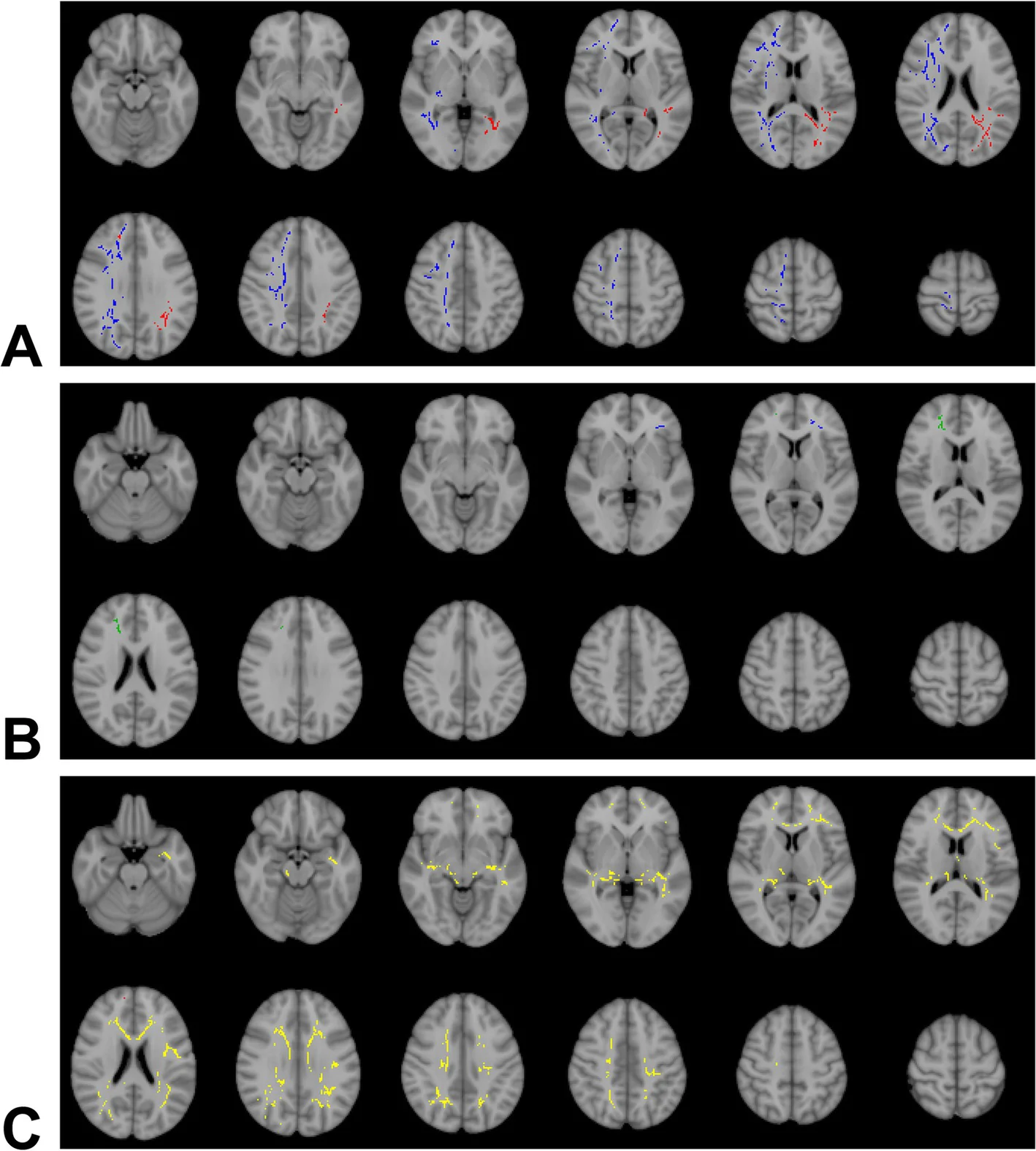
Components of each SPC derived from SPCA of TBSS differences in the PD group, showing only strong loadings (|loading| ≥ 0.3), SPCs were derived from imaging measures extracted from TBSS clusters identified using 5,000 permutation tests with TFCE correction at *p* < 0.05. **(A)** SPC1_PD – Colored in blue for fwRD and red for fwMD. Consists of: fwMDclu1 (0.415), fwMDclu5 (0.374), fwRDclu10 (0.328), fwMDclu8 (0.327), and fwMDclu2 (0.321). **(B)** SPC2_PD – Colored in blue for fwRD and green for fwFA. Consists of: fwFAclu7 (−0.675), fwRDclu3 (0.419), and fwRDclu5 (0.339). **(C)** SPC3_PD – Colored in red for fwMD and yellow for FW. Consists of: FWclu6 (−0.647) and fwMDclu3 (−0.442). SPC, sparse principal component; SPCA, sparse principal component analysis; TBSS, tract-based spatial statistics; PD, Parkinson’s disease; fwRD, free-water corrected radial diffusivity; fwMD, free-water corrected mean diffusivity; fwFA, free-water corrected fractional anisotropy; FW, free-water volume fraction. Cluster numbers are indicated as “clu” followed by their identifier.

In the TBSS analysis investigating differences between the APD and nAPD groups, 5,000 permutation tests revealed 3 significant FW clusters, all showing higher values in the APD group compared to the nAPD group. Among these 3 clusters, 2 were clearly located on the left side, while the cluster with the largest number of voxels was bilateral but with a centroid/peak lateralized to the left hemisphere, mainly involving all parts of the corpus callosum, the left superior longitudinal fasciculus, the uncinate fasciculus, and the inferior fronto-occipital fasciculus. The largest cluster encompassed almost all major white matter tracts bilaterally (Table 6 and Figure 4). Due to the limited number of significant results, SPCA analysis was not performed.

**Table 6:** Summary of FW TBSS differences between the APD and nAPD groups. All reported clusters showed higher FW values in APD than in nAPD.

Cluster	JHU white-matter Tractography Atlas	JHU ICBM-DTI-81 white-matter labels	Side	Voxel	*p*-value	MNI coordinate X	MNI coordinate Y	MNI coordinate Z
1	Uncinate fasciculus/Inferior fronto-occipital fasciculus/Anterior thalamic radiation	Unclassified	L	26	<0.050	−37.2	36	−4.92
2	Superior longitudinal fasciculus/Superior longitudinal fasciculus (temporal part)/Corticospinal tract	Unclassified	L	1,096	<0.001	−31.1	−11.1	43
3	Forceps minor/Anterior thalamic radiation/Corticospinal tract/Cingulum/Inferior fronto-occipital fasciculus/Superior longitudinal fasciculus/Uncinate fasciculus	Genu of corpus callosum/Body of corpus callosum/Splenium of corpus callosum/Corona radiata/Internal capsule/Posterior thalamic radiation	Bilateral	18,911	<0.001	−7.05	−11.7	20.7

Group differences were assessed using 5,000 permutation tests with threshold-free cluster enhancement (TFCE) correction, and only clusters surviving TFCE-corrected *p* < 0.05 are reported. MNI coordinates are given in millimeters. Anatomical locations were identified using the JHU White-Matter Tractography Atlas and the JHU ICBM-DTI-81 White-Matter Labels.

FW, free-water volume fraction; TBSS, tract-based spatial statistics; APD, apathetic Parkinson’s disease; nAPD, non-apathetic Parkinson’s disease; MNI, Montreal Neurological Institute; TFCE, threshold-free cluster enhancement.

**Figure 4:**
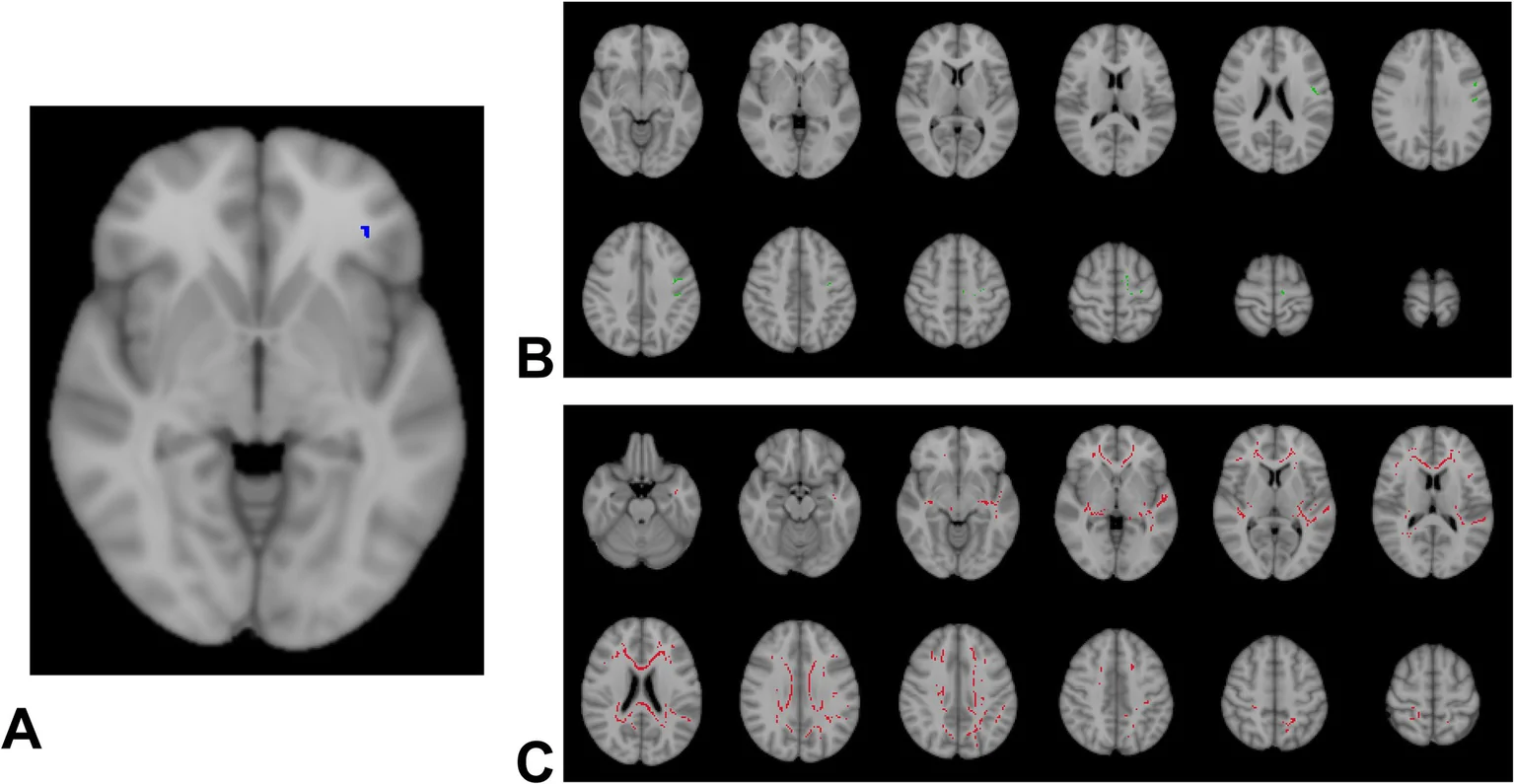
Significant FW clusters identified from TBSS comparing APD and nAPD patients. After 5,000 permutations, three clusters showed significantly higher FW values in the APD group than in the nAPD group (all *p* < 0.001, threshold-free cluster enhancement correction). **(A)** FWcluster1 (blue) – Involves the uncinate fasciculus, inferior fronto-occipital fasciculus, and anterior thalamic radiation according to the JHU White-Matter Tractography Atlas. **(B)** FWcluster2 (green) – Involves the superior longitudinal fasciculus, superior longitudinal fasciculus (temporal part), and corticospinal tract (JHU White-Matter Tractography Atlas). **(C)** FWcluster3 (red) – According to the JHU White-Matter Tractography Atlas, involves the forceps minor, anterior thalamic radiation, corticospinal tract, cingulum, inferior fronto-occipital fasciculus, superior longitudinal fasciculus, and uncinate fasciculus. According to the JHU ICBM-DTI-81 White-Matter Labels, it involves the genu, body, and splenium of the corpus callosum; corona radiata; internal capsule; and posterior thalamic radiation. FW, free-water volume fraction; TBSS, tract-based spatial statistics; APD, apathetic PD; nAPD, non-apathetic PD. Cluster nomenclature follows the JHU atlases as indicated.

In the TBSS analysis comparing the APD and HC groups, 5,000 permutation tests identified 45 significant fiso clusters. Of these, 8 clusters showed higher values in the HC group than in the APD group, primarily concentrated in the corpus callosum, cerebral peduncles, and superior cerebellar peduncles, while 37 clusters showed higher values in the APD group than in the HC group, covering almost all white matter regions (Supplementary Table S10). Additionally, 4 significant fwAD clusters were found, all showing higher values in the APD group than in the HC group, involving the corpus callosum, bilateral internal capsule, and corona radiata (Supplementary Table S11). Four significant fwFA clusters were identified, all showing higher values in the HC group than in the APD group, covering the forceps minor of the corpus callosum, corticospinal tract, and anterior thalamic radiation (Supplementary Table S12). One significant fwMD cluster showed higher values in the APD group than in the HC group, with extensive involvement (Supplementary Table S13). Two significant fwRD clusters were found, both showing higher values in the APD group than in the HC group, mainly involving the right hemisphere corpus callosum and corona radiata (Supplementary Table S14). One significant FW cluster showed higher values in the APD group than in the HC group, with extensive bilateral involvement (Supplementary Table S15). One significant NDI cluster showed higher values in the HC group than in the APD group, covering almost all types of white matter fiber tracts (Supplementary Table S16), and 5 significant ODI clusters showed higher values in the APD group than in the HC group, covering the corpus callosum and occipitotemporal association fibers (Supplementary Table S17). In the comparison between the nAPD and HC groups, 36 significant fiso clusters were identified. Of these, 8 clusters showed higher values in the HC group than in the nAPD group, primarily concentrated in the corpus callosum, superior cerebellar peduncles, and cerebral peduncles. There were 28 clusters showing higher values in the nAPD group than in the HC group, with two large clusters located in the left and right hemispheres, respectively, in a separated and extensive state (Supplementary Table S18). Additionally, 4 significant NDI clusters were found, all showing higher values in the HC group than in the nAPD group, mainly affecting the superior longitudinal fasciculus, posterior corona radiata, posterior thalamic radiation, and corpus callosum (Supplementary Table S19).

### 3.4 Correlations among glymphatic-related MRI metrics, white matter microstructural abnormalities, and apathy

In the correlation analysis between glymphatic-related MRI metrics and the severity of apathy symptoms, the maximum variance inflation factor (VIF_max) for all partial correlation models was less than 5, indicating no significant multicollinearity among control variables and stable model estimates. When all subjects were included: after controlling for age and sex, AES was significantly positively correlated with CPV, CPV_right, and CPV_left before FDR correction (CPV: *r* = 0.263, *p* = 0.021; CPV_right: *r* = 0.258, *p* = 0.023; CPV_left: *r* = 0.232, *p* = 0.042); after further controlling for SAS and SDS, AES was significantly negatively correlated with PVS_WM, PVS_SUB, and pPVS before FDR correction (PVS_WM: *r* = −0.250, *p* = 0.031; PVS_SUB: *r* = −0.235, *p* = 0.042; pPVS: *r* = −0.232, *p* = 0.045), and with Positive coupling after FDR correction (*r* = −0.326, *p_FDR_* = 0.047); no significant correlations were found after controlling for MoCA; in the fully adjusted model (age, sex, SAS, SDS, MoCA), AES was significantly negatively correlated with PVS_SUB and Positive coupling before FDR correction (PVS_SUB: *r* = −0.244, *p* = 0.036; Positive coupling: *r* = −0.342, *p* = 0.032) (Supplementary Tables S20–S24). In the PD group, only in the fully adjusted model (age, sex, disease duration, SAS, SDS, MoCA, LEDD, UPDRS-III) was AES significantly positively correlated with ALPS_right before FDR correction (*r* = 0.352, *p* = 0.045), while no significant correlations were observed in other models (Supplementary Table S21). In the HC group, after controlling for age and sex, AES was significantly negatively correlated with Positive coupling before FDR correction (*r* = −0.342, *p* = 0.041); after further controlling for SAS and SDS (*r* = −0.448, *p* = 0.008), after controlling for MoCA (*r* = −0.361, *p* = 0.033), and in the fully adjusted model (*r* = −0.398, *p* = 0.022), this negative correlation remained significant before FDR correction (Supplementary Table S22). Finally, in the APD and nAPD groups, AES in the APD group showed no significant correlations with any glymphatic-related MRI metrics across all control models; in the nAPD group, after controlling for age, sex, and disease duration, AES was significantly negatively correlated with PVS_SUB before FDR correction (*r* = −0.470, *p* = 0.042), and after further controlling for SAS and SDS, this negative correlation remained significant (*r* = −0.508, *p* = 0.037), while no significant correlations were found in other models (Supplementary Tables S23, S24).

To delineate the biological continuum, we performed exploratory partial correlation analyses in the entire sample. After controlling for age and sex, AES was significantly positively correlated with SPC1_All (*r* = 0.364, *p_FDR_* = 0.016), and significantly negatively correlated with SPC2_All (*r* = −0.524, *p_FDR_* < 0.001), SPC3_All (*r* = −0.617, *p_FDR_* < 0.001), SPC4_All (*r* = −0.307, *p_FDR_* = 0.036), and SPC5_All (*r* = −0.524, *p_FDR_* < 0.001). CPV was significantly positively correlated with SPC1_All (*r* = 0.451, *p_FDR_* = 0.001), and significantly negatively correlated with SPC3_All (*r* = −0.410, *p_FDR_* = 0.004) and SPC5_All (*r* = −0.362, *p_FDR_* = 0.016). CPV_right was significantly positively correlated with SPC1_All (*r* = 0.456, *p_FDR_* = 0.001), and significantly negatively correlated with SPC3_All (*r* = −0.430, *p_FDR_* = 0.002) and SPC5_All (*r* = −0.346, *p_FDR_* = 0.019). CPV_left was significantly positively correlated with SPC1_All (*r* = 0.396, *p_FDR_* = 0.006), and significantly negatively correlated with SPC3_All (*r* = −0.340, *p_FDR_* = 0.020) and SPC5_All (*r* = −0.340, *p_FDR_* = 0.020). pPVS and PVS_SUB showed no significant correlations. PVS_WM was significantly positively correlated with SPC4_All (*r* = 0.321, *p_FDR_* = 0.032). ALPS was significantly positively correlated with SPC2_All (*r* = 0.312, *p_FDR_* = 0.035) and SPC4_All (*r* = 0.357, *p_FDR_* = 0.017). ALPS_left was significantly positively correlated with SPC4_All (*r* = 0.393, *p_FDR_* = 0.007). ALPS_right was significantly positively correlated with SPC2_All (*r* = 0.323, *p_FDR_* = 0.031). After controlling for age, sex, SAS, and SDS, AES was significantly negatively correlated with SPC2_All (*r* = −0.352, *p_FDR_* = 0.032), SPC3_All (*r* = −0.467, *p_FDR_* = 0.004), and SPC5_All (*r* = −0.331, *p_FDR_* = 0.041). CPV was significantly positively correlated with SPC1_All (*r* = 0.425, *p_FDR_* = 0.006), and significantly negatively correlated with SPC3_All (*r* = −0.363, *p_FDR_* = 0.025). CPV_right was significantly positively correlated with SPC1_All (*r* = 0.426, *p_FDR_* = 0.006), and significantly negatively correlated with SPC3_All (*r* = −0.381, *p_FDR_* = 0.019). CPV_left was significantly positively correlated with SPC1_All (*r* = 0.376, *p_FDR_* = 0.020). ALPS was significantly positively correlated with SPC2_All (*r* = 0.326, *p_FDR_* = 0.041) and SPC4_All (*r* = 0.365, *p_FDR_* = 0.025). ALPS_left was significantly positively correlated with SPC4_All (*r* = 0.413, *p_FDR_* = 0.008). ALPS_right was significantly positively correlated with SPC2_All (*r* = 0.322, *p_FDR_* = 0.043). After controlling for age, sex, and MoCA, AES was significantly negatively correlated with SPC2_All (*r* = −0.376, *p_FDR_* = 0.019), SPC3_All (*r* = −0.469, *p_FDR_* = 0.003), and SPC5_All (*r* = −0.395, *p_FDR_* = 0.014). CPV was significantly positively correlated with SPC1_All (*r* = 0.396, *p_FDR_* = 0.014), and significantly negatively correlated with SPC3_All (*r* = −0.323, *p_FDR_* = 0.043). CPV_right was significantly positively correlated with SPC1_All (*r* = 0.389, *p_FDR_* = 0.014), and significantly negatively correlated with SPC3_All (*r* = −0.321, *p_FDR_* = 0.043). CPV_left was significantly positively correlated with SPC1_All (*r* = 0.361, *p_FDR_* = 0.027), and significantly negatively correlated with SPC3_All (*r* = −0.290, *p_FDR_* = 0.011). ALPS was significantly positively correlated with SPC4_All (*r* = 0.346, *p_FDR_* = 0.035). ALPS_left was significantly positively correlated with SPC4_All (*r* = 0.388, *p_FDR_* = 0.014). ALPS_right was significantly positively correlated with SPC2_All (*r* = 0.310, *p_FDR_* = 0.049). In the fully adjusted model (age, sex, SAS, SDS, MoCA), AES was significantly negatively correlated with SPC3_All (*r* = −0.384, *p_FDR_* = 0.022). CPV was significantly positively correlated with SPC1_All (*r* = 0.398, *p_FDR_* = 0.020). CPV_right was significantly positively correlated with SPC1_All (*r* = 0.392, *p_FDR_* = 0.020). CPV_left was significantly positively correlated with SPC1_All (*r* = 0.361, *p_FDR_* = 0.039). ALPS was significantly positively correlated with SPC4_All (*r* = 0.358, *p_FDR_* = 0.039). ALPS_left was significantly positively correlated with SPC4_All (*r* = 0.393, *p_FDR_* = 0.020) (Supplementary Table S25). Exploratory analyses showed that SAS was significantly positively correlated with SPC1_All (*r* = 0.310, *p_FDR_* = 0.025), and significantly negatively correlated with SPC2_All (*r* = −0.364, *p_FDR_* = 0.006), SPC3_All (*r* = −0.448, *p_FDR_* < 0.001), SPC4_All (*r* = −0.290, *p_FDR_* = 0.040), and SPC5_All (*r* = −0.459, *p_FDR_* < 0.001). MoCA was significantly negatively correlated with SPC1_All (*r* = −0.318, *p_FDR_* = 0.022), and significantly positively correlated with SPC2_All (*r* = 0.420, *p_FDR_* < 0.001), SPC3_All (*r* = 0.481, *p_FDR_* < 0.001), and SPC5_All (*r* = 0.389, *p_FDR_* = 0.003) (Supplementary Table S26).

Subsequently, including only PD patients, we analyzed the correlations between AES, glymphatic-related MRI metrics, and the 3 SPCs derived from SPCA_PD. In addition to age and sex, the effect of disease duration was also controlled for. The partial correlation results (Figure 5A) showed that AES was significantly negatively correlated with SPC3_PD (*r* = −0.493, *p_FDR_* = 0.011). CPV was significantly positively correlated with SPC1_PD (*r* = 0.446, *p_FDR_* = 0.017) and SPC2_PD (*r* = 0.680, *p_FDR_* < 0.001), and significantly negatively correlated with SPC3_PD (*r* = −0.490, *p_FDR_* = 0.011). CPV_right was significantly positively correlated with SPC1_PD (*r* = 0.473, *p_FDR_* = 0.014) and SPC2_PD (*r* = 0.674, *p_FDR_* < 0.001), and significantly negatively correlated with SPC3_PD (*r* = −0.490, *p_FDR_* = 0.011). CPV_left was significantly positively correlated with SPC2_PD (*r* = 0.618, *p_FDR_* < 0.001), and significantly negatively correlated with SPC3_PD (*r* = −0.441, *p_FDR_* = 0.017). PVS and PVS_SUB showed no significant correlations. PVS_WM was significantly negatively correlated with SPC1_PD (*r* = −0.467, *p_FDR_* = 0.014), and significantly positively correlated with SPC3_PD (*r* = 0.388, *p_FDR_* = 0.044). ALPS was significantly negatively correlated with SPC1_PD (*r* = −0.449, *p_FDR_* = 0.017). ALPS_left was significantly negatively correlated with SPC1_PD (*r* = −0.456, *p_FDR_* = 0.016). ALPS_right showed no significant correlation. After controlling for age, sex, disease duration, SAS, and SDS, CPV was significantly positively correlated with SPC1_PD (*r* = 0.444, *p_FDR_* = 0.027) and SPC2_PD (*r* = 0.678, *p_FDR_* < 0.001), and significantly negatively correlated with SPC3_PD (*r* = −0.464, *p_FDR_* = 0.023). CPV_right was significantly positively correlated with SPC1_PD (*r* = 0.484, *p_FDR_* = 0.022) and SPC2_PD (*r* = 0.680, *p_FDR_* < 0.001), and significantly negatively correlated with SPC3_PD (*r* = −0.468, *p_FDR_* = 0.023). CPV_left was significantly positively correlated with SPC2_PD (*r* = 0.608, *p_FDR_* = 0.001), and significantly negatively correlated with SPC3_PD (*r* = −0.413, *p_FDR_* = 0.045). ALPS was significantly negatively correlated with SPC1_PD (*r* = −0.451, *p_FDR_* = 0.026). ALPS_left was significantly negatively correlated with SPC1_PD (*r* = −0.466, *p_FDR_* = 0.023). After controlling for age, sex, disease duration, and MoCA, AES was significantly negatively correlated with SPC3_PD (*r* = −0.417, *p_FDR_* = 0.033). CPV was significantly positively correlated with SPC1_PD (*r* = 0.414, *p_FDR_* = 0.033) and SPC2_PD (*r* = 0.668, *p_FDR_* < 0.001), and significantly negatively correlated with SPC3_PD (*r* = −0.451, *p_FDR_* = 0.031). CPV_right was significantly positively correlated with SPC1_PD (*r* = 0.433, *p_FDR_* = 0.031) and SPC2_PD (*r* = 0.665, *p_FDR_* < 0.001), and significantly negatively correlated with SPC3_PD (*r* = −0.435, *p_FDR_* = 0.031). CPV_left was significantly positively correlated with SPC2_PD (*r* = 0.612, *p_FDR_* < 0.001), and significantly negatively correlated with SPC3_PD (*r* = −0.431, *p_FDR_* = 0.031). PVS_WM was significantly negatively correlated with SPC1_PD (*r* = −0.422, *p_FDR_* = 0.033). ALPS was significantly negatively correlated with SPC1_PD (*r* = −0.440, *p_FDR_* = 0.031). ALPS_left was significantly negatively correlated with SPC1_PD (*r* = −0.450, *p_FDR_* = 0.031). After controlling for age, sex, disease duration, LEDD, and UPDRS-III, AES was significantly negatively correlated with SPC3_PD (*r* = −0.489, *p_FDR_* = 0.013). CPV was significantly positively correlated with SPC1_PD (*r* = 0.441, *p_FDR_* = 0.021) and SPC2_PD (*r* = 0.677, *p_FDR_* < 0.001), and significantly negatively correlated with SPC3_PD (*r* = −0.490, *p_FDR_* = 0.013). CPV_right was significantly positively correlated with SPC1_PD (*r* = 0.464, *p_FDR_* = 0.020) and SPC2_PD (*r* = 0.671, *p_FDR_* < 0.001), and significantly negatively correlated with SPC3_PD (*r* = −0.489, *p_FDR_* = 0.013). CPV_left was significantly positively correlated with SPC2_PD (*r* = 0.623, *p_FDR_* < 0.001), and significantly negatively correlated with SPC3_PD (*r* = −0.446, *p_FDR_* = 0.021). PVS_WM was significantly negatively correlated with SPC1_PD (*r* = −0.503, *p_FDR_* = 0.013), and significantly positively correlated with SPC3_PD (*r* = 0.441, *p_FDR_* = 0.021). ALPS was significantly negatively correlated with SPC1_PD (*r* = −0.437, *p_FDR_* = 0.021). ALPS_left was significantly negatively correlated with SPC1_PD (*r* = −0.446, *p_FDR_* = 0.021) (Supplementary Table S27). In the fully adjusted model (age, sex, disease duration, SAS, SDS, MoCA, LEDD, UPDRS-III), no significant correlations were observed after FDR correction (Supplementary Table S28).

**Figure 5:**
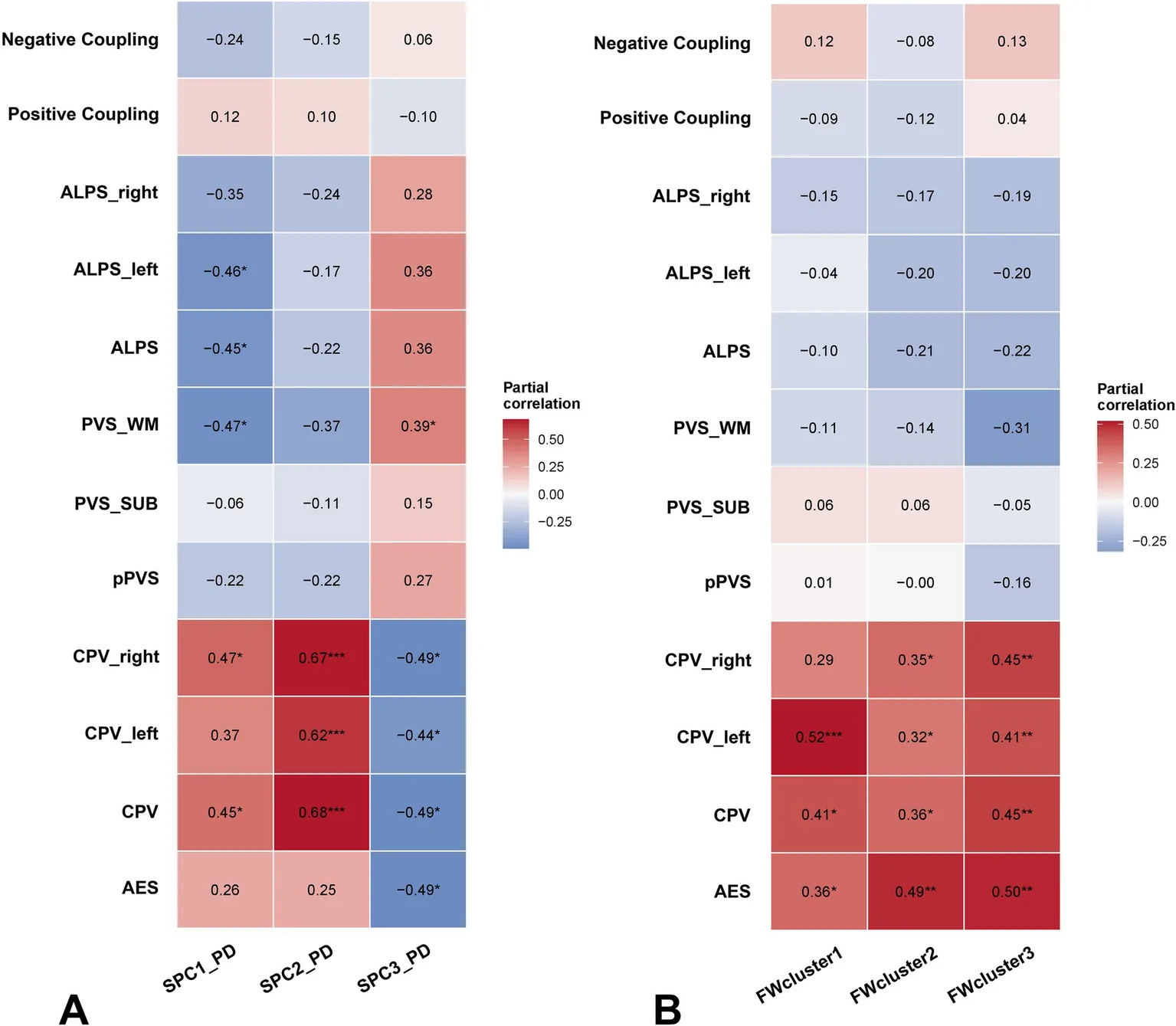
Partial correlation analysis of glymphatic-related MRI metrics, microstructural damage, and apathy in the PD group. All partial correlations were adjusted for age, sex, and disease duration. **(A)** Microstructural damage was represented by three SPCs derived from SPCA of TBSS differences between PD patients and HC. **(B)** Microstructural damage was represented by three FW clusters that showed significant differences between APD and nAPD patients in TBSS analysis. *p*-values in **(A)** were corrected for multiple comparisons using the FDR. *P*-values in **(B)** are shown before FDR correction (no significant correlations remained after FDR correction). Significance levels: *p < 0.05, **p < 0.01, ***p < 0.001. PD, Parkinson’s disease; HC, healthy controls; APD, apathetic PD; nAPD, non-apathetic PD; TBSS, tract-based spatial statistics; SPCA, sparse principal component analysis; SPC, sparse principal component; FW, free-water; FDR, false discovery rate; AES, Apathy Evaluation Scale; CPV, choroid plexus volume; CPV_left, left hemisphere CPV; CPV_right, right hemisphere CPV; PVS, perivascular space volume; PVS_SUB, PVS in subcortical gray matter nuclei; PVS_WM, PVS in the white matter region; pPVS, whole-brain PVS; ALPS, index of diffusion along perivascular spaces; ALPS_left, left hemisphere ALPS; ALPS_right, right hemisphere ALPS.

Using the significant microstructural damage metrics from the TBSS comparison between the APD and nAPD groups, we performed partial correlation analyses in the PD group (Figure 5B). In the core analysis controlling for age, sex, and disease duration, AES was significantly positively correlated with FWcluster1 (*r* = 0.359, *p_FDR_* = 0.027), FWcluster2 (*r* = 0.493, *p_FDR_* = 0.020), and FWcluster3 (*r* = 0.501, *p_FDR_* = 0.020); CPV was significantly positively correlated with FWcluster3 (*r* = 0.453, *p_FDR_* = 0.036); CPV_right was significantly positively correlated with FWcluster3 (*r* = 0.447, *p_FDR_* = 0.036); and CPV_left was significantly positively correlated with FWcluster1 (*r* = 0.518, *p_FDR_* = 0.020). After controlling for age, sex, disease duration, and MoCA, CPV_left remained significantly positively correlated with FWcluster1 (*r* = 0.512, *p_FDR_* = 0.043). After controlling for age, sex, disease duration, LEDD, and UPDRS-III, AES was significantly positively correlated with FWcluster2 (*r* = 0.493, *p_FDR_* = 0.027) and FWcluster3 (*r* = 0.506, *p_FDR_* = 0.027); CPV_left was significantly positively correlated with FWcluster1 (*r* = 0.520, *p_FDR_* = 0.027). After controlling for age, sex, disease duration, SAS, and SDS, as well as in the fully adjusted model (age, sex, disease duration, SAS, SDS, MoCA, LEDD, UPDRS-III), no significant correlations were observed after FDR correction (Supplementary Table S29). Exploratory analyses in the PD group showed that SAS was significantly positively correlated with FWcluster3 (*r* = 0.344, *p_FDR_* = 0.035) (Supplementary Table S30). In the APD group (Figure 6A), no significant correlations were observed after FDR correction across all control models (including the core analysis and models controlling for SAS and SDS, MoCA, LEDD and UPDRS-III, and the fully adjusted model) (Supplementary Table S31); in exploratory analyses, no significant correlations were found between SAS, SDS, or MoCA and any of the three FW clusters (Supplementary Table S32). In the nAPD group (Figure 6B), no significant correlations were observed after FDR correction across all control models (including the core analysis and models controlling for SAS and SDS, MoCA, LEDD and UPDRS-III, and the fully adjusted model) (Supplementary Table S33); exploratory analyses showed that MoCA was significantly negatively correlated with FWcluster2 (*r* = −0.637, *p_FDR_* = 0.030), while SAS and SDS showed no significant correlations with any FW cluster (Supplementary Table S34).

**Figure 6:**
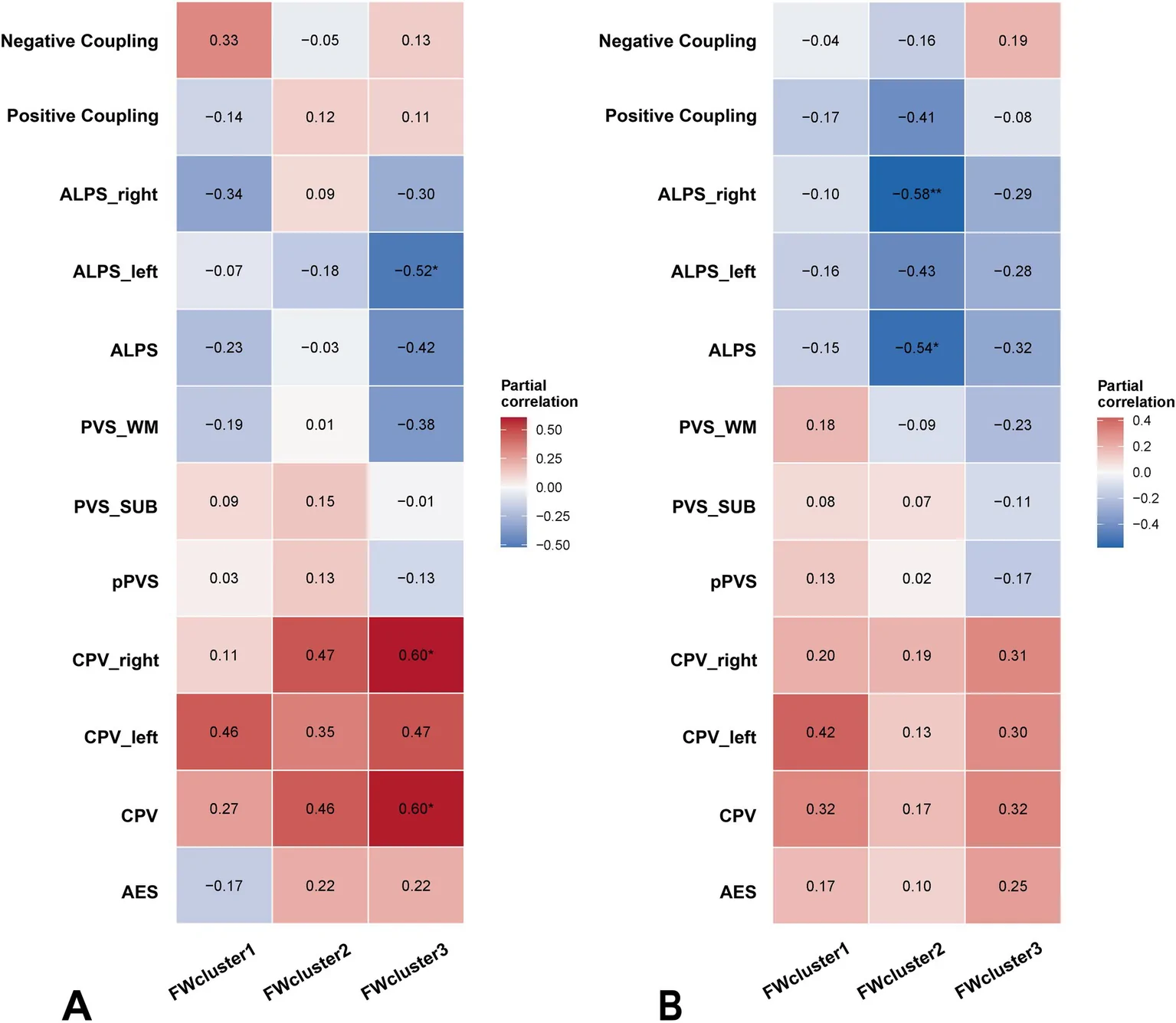
Partial correlation analysis of glymphatic-related MRI metrics, microstructural damage, and apathy in the APD and nAPD groups. All partial correlations were adjusted for age, sex, and disease duration. **(A)** Partial correlation results in the APD group. **(B)** Partial correlation results in the nAPD group. All *p*-values are presented without false discovery rate (FDR) correction (i.e., uncorrected *p*-values). Significance levels: *p < 0.05, **p < 0.01. APD, apathetic PD; nAPD, non-apathetic PD; FW, free-water; FDR, false discovery rate; AES, Apathy Evaluation Scale; CPV, choroid plexus volume; CPV_left, left hemisphere CPV; CPV_right, right hemisphere CPV; PVS, perivascular space volume; PVS_SUB, PVS in subcortical gray matter nuclei; PVS_WM, PVS in the white matter region; pPVS, whole-brain PVS; ALPS, index of diffusion along perivascular spaces; ALPS_left, left hemisphere ALPS; ALPS_right, right hemisphere ALPS.

### 3.5 Exploratory mediation analysis of glymphatic-related MRI metrics, white matter microstructural abnormalities, and clinical symptoms

Consistent with the correlation analysis, we first conducted exploratory mediation analyses across diagnostic categories in the entire sample to assess characteristics of the biological continuum. First, we included all subjects (PD + HC) for analysis. The microstructural damage metrics were the 5 SPCs obtained after SPCA on the TBSS analysis results between the PD and HC groups within the entire sample range. The outcome variable was AES. A total of 17 significant pathways were found after FDR correction (Supplementary Table S35). Among them, 10 were full mediation effects, mainly concentrated in the CPV series metrics (CPV, CPV_left, CPV_right) - SPC1_All / SPC2_All / SPC3_All / SPC5_All - AES. Specifically, CPV and CPV_left indirectly affected AES by decreasing SPC3_All / SPC5_All, while the direct effects of the CPV series on AES were not significant after controlling for SPC3_All / SPC5_All, indicating that SPC3_All / SPC5_All are necessary mediators for the CPV series to influence apathy. In contrast, the paths for CPV with SPC2_All / SPC1_All were opposite to the above; CPV indirectly increased AES by increasing SPC1_All / SPC2_All. There was 1 partial mediation effect: CPV_right - SPC2_All - AES, where only 37% of the effect was through SPC2_All, suggesting that in addition to SPC2_All, there are other neural mechanisms for the influence of CPV_right on apathy. There were 6 suppression effects, of which 2 were competitive suppression: SPC2_All - ALPS / ALPS_right - AES. The higher the SPC2_All, the lower the AES; however, SPC2_All indirectly increased AES by improving ALPS / ALPS_right, and the two effects canceled each other out. The remaining 4 were suppressed total effects: ALPS series - SPC2_All / SPC4_All - AES. Although the total effect was not significant, there was a significant “one side damage, one side protection” mechanism internally, meaning that ALPS indirectly decreased AES by increasing SPC2_All / SPC4_All, producing a negative indirect effect. This protective mediation effect canceled out the positive direct effect of ALPS on AES, leading to an apparent lack of association between ALPS and apathy.

Using the 3 SPCs from the within-PD group SPCA results, the mediation analysis within the PD group showed that after FDR correction, the indirect effects of all pathways did not reach significance (Supplementary Table S36). However, trend-level significance (*p* < 0.05 before FDR correction and 95% CI not including 0) still existed. The CPV series metrics (CPV, CPV_left, CPV_right) showed a trend-level indirect effect on AES through SPC3_PD, with a pattern close to full mediation. The CPV series indirectly decreased AES by decreasing SPC3_PD, producing a positive indirect effect. After controlling for SPC3_PD, the direct effects of the CPV series on AES were not significant, and the total effect was also not significant, consistent with the trend characteristics of full mediation. One pathway showed a trend-level competitive suppression: SPC3_PD - ALPS - AES. An increase in SPC3_PD tended to decrease AES; however, SPC3_PD indirectly increased AES by increasing ALPS, producing a positive indirect effect. This protective mediation effect was in the same direction as the direct improvement effect of SPC3_PD, leading to a weakened absolute value of the total effect, presenting a competitive suppression trend. In addition, multiple pathways showed a suppressed total effect pattern, including ALPS series metrics - SPC3_PD - AES and PVS_WM - SPC3_PD - AES. Among them, an increase in the ALPS series tended to decrease AES by decreasing SPC3_PD, but the direct effect of ALPS on AES was positive, and the opposite directions led to a suppressed total effect. The latter showed a similar suppression trend: an increase in PVS_WM decreased AES by increasing SPC3_PD, but its direct effect was not significant, and the total effect did not reach significance.

Finally, we analyzed the mediation analysis based on the TBSS analysis results between the APD and nAPD groups (Supplementary Table S37). Similar to the previous analyses, we did not find significant pathways after FDR correction, but we found that there were still 3 trend-level significant pathways before FDR correction. All 3 pathways with FW cluster 3 as the mediator showed a trend of suppression effect. The CPV series metrics produced a positive indirect effect on apathy through the increase of FW cluster 3, that is, tending to increase AES, but this effect was masked by other unmeasured factors, leading to an apparent lack of significant association between glymphatic function and apathy.

## 4 Discussion

Using multimodal neuroimaging techniques, the present study systematically explored the relationship between apathy symptoms, glymphatic system dysfunction, and white matter microstructural damage in PD patients for the first time. The main findings include: (1) PD patients exhibit microstructural abnormalities spanning a widespread white matter skeleton; (2) The most stable and specific direct difference between APD and nAPD is the elevation of FW, rather than comprehensive abnormalities in glymphatic-related MRI metrics; (3) CPV shows significant coupling with specific white matter damage patterns, suggesting that brain waste clearance impairment is closely related to white matter degeneration, but the evidence for a mediation causal chain within the disease is insufficient.

### 4.1 Extensive white matter microstructural abnormalities in PD

Consistent with previous studies (Wei et al., 2024; Shah et al., 2024), the present study, using NODDI-related metrics based on free-water corrected diffusion parameters, demonstrated that PD patients exhibit widespread and multidimensional white matter microstructural abnormalities compared to HC. These include diffuse abnormalities characterized by decreased fwFA, increased fwAD/fwMD/fwRD/FW, decreased NDI, and increased ODI. The aforementioned abnormalities suggest that white matter alterations in PD are not limited to a single case level but may simultaneously involve multiple aspects, including myelin integrity disruption (fwRD) (Yao et al., 2025), axonal swelling/transport impairment (fwAD) (Jing et al., 2025), neurite density reduction (NDI), fiber orientation dispersion (ODI) (Wei et al., 2024; Mitchell et al., 2019), and interstitial edema (FW) (Xu et al., 2025). Overall, these results support the existence of a background of diffuse white matter damage in PD and provide a disease baseline reference for the subsequent identification of apathy-specific abnormalities.

It is noteworthy that the SPCA_PD model explained a larger proportion of variance than the SPCA_All model (67.54% vs. 53.94%). This suggests that when HC are included in the same covariance structure, part of the disease-internal heterogeneity might be diluted by case–control mean differences. Therefore, principal components within PD are more suitable as statistical representations characterizing co-varying patterns within the disease. The results of SPCA_ALL more likely reflect the macroscopic gradient of the transition from normal aging to disease state, confounded by mixed signals of compensation and decompensation. For example, SPC4_All demonstrated an antagonistic relationship between increased fwFA and decreased fwMD. In contrast, SPCA_PD extracted three highly specific core components: diffuse extracellular water increase (SPC1_PD), a frontal-temporal limbic white matter pattern characterized predominantly by lower fwFA and higher fwRD (SPC2_PD), and a distributed multidimensional white matter abnormality pattern (SPC3_PD). Among them, SPC1_PD is characterized by widespread fwMD elevation involving the bilateral corona radiata and corpus callosum, which may correspond to widespread neuroinflammatory responses or blood–brain barrier leakage, constituting the fundamental background of white matter damage in PD. SPC2_PD presents a unique “axon-myelin mismatch” feature, with high loading regions specifically concentrated in the right frontal lobe (forceps minor of corpus callosum) and left temporal lobe (uncinate fasciculus). These regions correspond precisely to the limbic system circuits that regulate motivation, emotion, and complex behavior (Zhang et al., 2023; Shen et al., 2022). SPC3_PD integrates abnormalities in multiple metrics such as FW and ODI, likely representing “global structural disintegration” in the late stages of the disease. These three SPCs more purely depict the major imaging-derived patterns of white matter variation within PD during the PD disease process. These extensive abnormalities constitute the disease baseline reference for the subsequent analysis of apathy-specific pathological changes.

### 4.2 Apathy-related white matter microenvironment abnormalities

Having established the pathological background of general white matter damage in PD, identifying specific neuroimaging biomarkers associated with the apathy phenotype is the core focus of this study. The results indicate that in the direct comparison between APD and nAPD, the most stable and significant intergroup differences were concentrated in the abnormal elevation of FW, while other microstructural diffusion metrics did not exhibit comparable stability. This phenomenon suggests that the imaging characteristics associated with PD apathy may primarily stem from alterations in the extracellular microenvironment of specific white matter tracts, rather than extensive or consistent structural damage to axons and neurites. As an MRI-derived estimate of the extracellular isotropic water fraction, increased FW may reflect several processes, including neuroinflammatory, vascular, blood–brain barrier, or interstitial-fluid changes are key events in the occurrence of apathy (Shah et al., 2024). In terms of anatomical distribution, the regions with elevated FW in APD patients were not random but specifically involved the left fronto-temporal association fibers, corpus callosum, and bilateral anterior thalamic radiation. These regions are precisely the core white matter structures of the fronto-limbic circuits and fronto-striatal pathways (De Waele et al., 2022; Gilmour et al., 2024; Zhang et al., 2023; Shen et al., 2022; Lucas-Jiménez et al., 2018). Based on this, it can be further speculated that the occurrence of apathy is closely related to the microenvironmental destabilization of the fronto-limbic and fronto-striatal pathways. Importantly, this categorical finding was further supported by dimensional analyses within the PD cohort: AES scores were positively associated with all three APD–nAPD FW clusters after controlling for age, sex, and disease duration. Thus, convergent evidence from both group-level and symptom-severity analyses suggests that increased extracellular free water represents a prominent white matter correlate of apathy in PD rather than merely a nonspecific consequence of the overall PD disease state.

From the perspective of anatomical spatial distribution characteristics, the FW elevation associated with APD is concentrated in regions such as the uncinate fasciculus, superior longitudinal fasciculus, corpus callosum, and anterior thalamic radiation. These white matter tracts constitute the important anatomical basis for the motivation, emotion, and behavioral regulation network. Combined with the SPCA_PD results, there is a certain spatial correspondence between APD-related FW abnormalities and the principal components representing white matter microenvironment alterations. Specifically, the FW differences (APD > nAPD) in the left superior longitudinal fasciculus (FW Cluster 2) and bilateral corona radiata (FW Cluster 3) significantly overlapped with SPC1_PD, which represents diffuse microstructural damage. Meanwhile, the elevated FW in the left uncinate fasciculus (FW Cluster 1) and forceps minor (FW Cluster 3) precisely corresponded to the anterior temporal limbic network defined by SPC2_PD. This result further suggests that apathy-related pathological changes may not exist in isolation but are deeply rooted in the evolutionary trajectory of overall white matter damage in PD. Furthermore, the comparison of TBSS analysis between APD vs. HC and nAPD vs. HC showed that the white matter damage in APD patients was more extensive and severe (NDI abnormal clusters reached 44,110 voxels), while the damage in nAPD patients was relatively localized and showed a state of bilateral dissociation (maximum cluster approximately 7,233 voxels). Simultaneously, our comparison of TBSS analysis results between APD vs. HC and nAPD vs. HC revealed that the cluster centroids of the most significant microstructural alterations in APD (FW elevation, NDI reduction) precisely corresponded to the columns and body of the fornix. This region connects the hippocampus and hypothalamus and serves as a key hub for emotion-motivation circuits (Manca et al., 2022; Shaikh et al., 2022). Damage here effectively severs the pathway between affective drive input and motivational output, suggesting fronto-limbic disconnection. Furthermore, APD-related microstructural impairments (such as increased ODI and abnormal fwRD) were more pronounced in the right hemisphere. This aligns with neuropsychological theories positing that the right hemisphere dominates novelty processing, vigilance, and socio-emotional processing (Kheirkhah et al., 2021). The preferential damage to the right association fiber networks may lead to patients’ difficulty in generating emotional responses to environmental changes, thereby manifesting as indifference to external stimuli (Filley, 2020). This suggests that apathy-related alterations emerge on the background of broader PD-related white matter pathology rather than representing an anatomically isolated lesion pattern. From a microstructural perspective, increased FW reflects expansion or alteration of the extracellular water compartment and may be influenced by neuroinflammatory, vascular, blood–brain barrier, or interstitial-fluid processes. Therefore, although the predominance of FW abnormalities points toward disruption of the extracellular white matter microenvironment, the present data do not allow attribution to a single underlying biological mechanism.

The sensitivity analyses further delineated the clinical specificity of the FW–apathy relationship. APD and nAPD did not differ significantly in UPDRS-III scores or LEDD, and the associations between AES and two of the major FW clusters remained significant after additional adjustment for motor severity and dopaminergic medication burden. These findings suggest that the observed FW abnormalities are unlikely to be explained primarily by differences in motor disease severity or dopaminergic treatment. In contrast, adjustment for anxiety and depressive symptoms attenuated the associations between AES and the FW clusters, while exploratory analyses demonstrated an independent association between SAS scores and FW cluster 3. This pattern suggests that part of the extracellular white matter abnormality associated with apathy may be shared with broader affective dysfunction rather than being exclusively attributable to apathy (Wen et al., 2016). A similar, although less direct, overlap may exist with cognitive function: MoCA scores did not differ significantly between APD and nAPD, arguing against a simple explanation based on greater cognitive impairment in the apathetic group, whereas attenuation of the cluster-level AES–FW associations after adjustment for MoCA suggests that motivational and cognitive dimensions may nevertheless share part of the same distributed white matter substrate.

Taken together, these findings support a model in which extracellular white matter abnormalities are closely linked to the clinical expression of apathy in PD but are not entirely symptom-specific. The persistence of the principal FW–apathy associations after accounting for motor severity and medication burden, together with their attenuation after accounting for affective and cognitive dimensions, suggests a combination of apathy-related and shared neuropsychiatric mechanisms (Marafioti et al., 2026). Accordingly, FW is more appropriately interpreted as a sensitive imaging correlate of the white matter microenvironment associated with PD apathy, embedded within broader affective and cognitive network dysfunction, rather than as a biomarker uniquely specific to apathy.

### 4.3 Coupling of glymphatic-related MRI metrics with white matter damage

In addition to the prominent FW abnormalities identified in APD, several glymphatic-related MRI metrics showed nominal differences between APD and nAPD, including larger left CPV, lower PVS_SUB volume fraction, and a less negative gBOLD–CSF negative coupling value. However, these between-group differences did not survive FDR correction and should therefore be regarded as exploratory rather than defining imaging features of the apathy phenotype. Moreover, CPV, PVS, ALPS, and gBOLD–CSF coupling represent biologically distinct aspects of intracranial fluid homeostasis, including choroid plexus status and CSF production (de Deus Vieira et al., 2024), perivascular space morphology (Jiang-Xie et al., 2025), local directional diffusion characteristics of water molecules (Mossige et al., 2026), and temporal interaction between global brain activity and CSF flow (Wang et al., 2023), respectively. Accordingly, the observed abnormalities should not be reduced to a unitary concept of “glymphatic failure,” but may instead reflect alterations across multiple components of brain fluid and metabolic homeostasis.

When considered specifically in relation to apathy, the glymphatic-related MRI findings showed a different pattern from the more robust FW abnormalities. Although APD patients exhibited nominally larger left CPV, lower PVS_SUB, and weaker gBOLD–CSF negative coupling than nAPD patients, these differences did not survive FDR correction, and direct associations between individual glymphatic-related MRI metrics and AES were not consistently reproduced within the PD or APD subgroups. This contrasts with the FW findings, for which both the APD–nAPD comparison and dimensional associations with AES converged. Therefore, rather than representing direct imaging markers of apathy themselves, CPV, PVS, ALPS, and gBOLD–CSF coupling may reflect broader alterations in intracranial fluid and metabolic homeostasis that provide a physiological context in which apathy-related white matter abnormalities occur. This interpretation is compatible with emerging evidence that MRI measures related to brain clearance processes are associated with multiple motor and non-motor manifestations of PD rather than with a single clinical phenotype (Wang Y. et al., 2026).

Within this broader context, CPV showed the clearest relationship with the white matter abnormalities linked to apathy. In the PD cohort, total and hemispheric CPV measures were significantly associated with the FW clusters that differentiated APD from nAPD, and the association between left CPV and FW cluster 1 remained significant after additional adjustment for MoCA and after adjustment for UPDRS-III and LEDD. The biological plausibility of this association is supported by the central role of the choroid plexus in CSF production, blood–CSF barrier regulation, immune surveillance, and maintenance of CNS homeostasis (Ghersi-Egea et al., 2018; Hutton et al., 2022). Importantly, an independent multimodal MRI study has recently demonstrated a significant positive association between choroid plexus volume fraction and white matter FW in PD, together with an inverse association between choroid plexus volume and the DTI-ALPS index (Wang Q. et al., 2026). Thus, our findings extend this previously observed CPV–FW relationship to white matter regions specifically associated with the apathy phenotype. Nevertheless, because the CPV–FW associations were attenuated after adjustment for anxiety and depressive symptoms, this coupling should not be regarded as specific to apathy. Instead, it may represent a shared homeostatic or neuroimmune process that contributes to the white matter substrate on which apathy and other affective symptoms are expressed.

The remaining glymphatic-related MRI metrics further support this cautious interpretation. Previous studies have demonstrated reduced gBOLD–CSF coupling in PD and associations of this measure with cognitive or motor manifestations (Han et al., 2021), while more recent multimodal evidence indicates that both DTI-ALPS and gBOLD–CSF coupling are also altered in PD patients with anxiety (Chen et al., 2025). These observations are consistent with our finding that associations between glymphatic-related MRI metrics and AES varied after adjustment for affective and cognitive symptoms, suggesting that these indices are not uniquely related to apathy. Against this multidimensional homeostatic background, however, FW remained the imaging feature most consistently linked to apathy in the present study: it differentiated APD from nAPD and correlated with AES severity within the PD cohort. We therefore propose that glymphatic-related MRI metrics abnormalities should be interpreted primarily as complementary physiological correlates of the white matter environment in which apathy emerges, whereas extracellular FW abnormalities provide the more immediate structural correlate of the apathy phenotype. The observed CPV–FW coupling offers a potential bridge between these two levels, but its temporal and causal direction cannot be established from the present cross-sectional data.

Exploratory mediation analyses were further used to examine whether the observed associations among glymphatic-related MRI metrics, white matter abnormalities, and apathy severity could be statistically organized within a common framework. Several mediation structures involving glymphatic-related MRI metrics, white matter components, and AES were observed in the combined PD + HC sample. These findings are broadly consistent with the possibility that alterations in intracranial fluid homeostasis and white matter microenvironmental damage occur along a shared biological spectrum associated with increasing apathy severity. However, these effects were substantially attenuated when analyses were restricted to the PD cohort and did not remain robust in the APD or nAPD subgroups after correction for multiple comparisons. Because the full-sample effects may partly reflect disease-related differences between PD and HC, they should not be interpreted as evidence for an apathy-specific causal pathway. Importantly, the mediation findings should be considered in the context of the more robust primary results. In the present study, FW abnormalities both differentiated APD from nAPD and were dimensionally associated with AES severity, whereas associations between individual glymphatic-related MRI metrics and AES were less consistent. At the same time, CPV showed significant coupling with APD-related FW abnormalities within the PD cohort. Taken together, these observations suggest a hierarchical associative pattern in which glymphatic- and choroid plexus-related alterations may reflect a broader intracranial homeostatic context, white matter extracellular abnormalities are more closely linked to the apathy phenotype, and the two levels are partially coupled. The exploratory mediation results provide supplementary support for this framework but do not establish the temporal or causal sequence among these processes. Longitudinal studies will therefore be required to determine whether alterations in intracranial fluid homeostasis precede, accompany, or follow the white matter changes associated with apathy in PD.

### 4.4 Summary and limitations

Overall, the present study identifies white matter extracellular microenvironmental abnormalities as the imaging feature most closely associated with apathy in PD. Against a background of widespread multidimensional white matter damage in PD, elevated FW distinguished APD from nAPD and showed dimensional associations with AES severity, particularly within white matter pathways involved in motivational, affective, and higher-order associative processing. These findings suggest that extracellular white matter abnormalities may represent a relatively proximal imaging correlate of the apathy phenotype. In comparison, individual glymphatic-related MRI metrics showed less consistent direct associations with apathy severity. Nevertheless, the significant coupling between CPV and apathy-related FW abnormalities indicates that alterations in choroid plexus-related homeostasis may be linked to the white matter microenvironment in which apathy emerges. The attenuation of some associations after adjustment for affective and cognitive symptoms further suggests that these processes are not entirely specific to apathy but may partly overlap with broader neuropsychiatric dysfunction in PD. Collectively, our findings support an associative framework in which glymphatic- and choroid plexus-related homeostatic alterations are coupled with white matter microstructural abnormalities, while FW abnormalities appear more directly related to the clinical expression of apathy. The present data do not establish a unidirectional pathological cascade.

Several limitations should be considered. First, the cross-sectional design precludes determination of the temporal sequence among glymphatic-related MRI metrics alterations, white matter abnormalities, and the development of apathy. Consequently, the directionality of the observed CPV–FW and imaging–symptom associations cannot be established. Second, the overall sample size was modest, particularly after stratification into APD and nAPD, which reduced statistical power for subgroup analyses and may have contributed to the instability of some correlations and mediation effects after multiple-comparison correction. The exploratory mediation analyses were therefore adjusted only for age, sex, and disease duration to limit model overfitting; potential effects of anxiety, depression, cognition, motor severity, and dopaminergic medication burden could not be simultaneously incorporated into these models. Third, apathy in PD frequently coexists with affective and cognitive symptoms. Although sensitivity analyses additionally considered SAS, SDS, MoCA, UPDRS-III, and LEDD, several FW- and CPV-related associations were attenuated after adjustment for affective or cognitive measures. These findings suggest partial overlap among motivational, affective, and cognitive dimensions, and residual shared neurobiological variance cannot be completely separated in the present sample. Importantly, APD and nAPD did not differ significantly in UPDRS-III or LEDD, and several principal FW associations remained after adjustment for these variables, arguing against a simple explanation based on motor severity or dopaminergic medication burden alone. Fourth, CPV, PVS, ALPS, and gBOLD–CSF coupling are indirect MRI markers of different components of intracranial fluid and homeostatic physiology rather than direct measurements of glymphatic clearance. Their biological meanings are not interchangeable, and abnormalities across these measures should therefore not be collectively interpreted as evidence of a unitary “glymphatic dysfunction.” Finally, age and disease duration showed nominal differences between APD and nAPD. Although these variables were included as covariates in the relevant analyses, residual confounding cannot be excluded. In addition, mediation effects observed predominantly in the combined PD + HC sample may partly reflect disease-related differences between diagnostic groups rather than mechanisms specific to apathy within PD. Future studies with larger samples, longitudinal follow-up, and independent multimodal validation will be required to determine whether glymphatic-related homeostatic alterations precede, accompany, or follow the development of white matter extracellular abnormalities and apathy in PD.

## 5 Conclusion

This study integrated multiple glymphatic-related MRI metrics with advanced diffusion imaging to characterize the relationships among intracranial homeostatic alterations, white matter microstructural abnormalities, and apathy in Parkinson’s disease. The main findings are as follows: (1) PD patients exhibited widespread multidimensional white matter abnormalities, providing a structural background against which apathy-related alterations emerge. (2) Increased white matter free water represented the most consistent imaging feature associated with apathy, distinguishing APD from nAPD and showing dimensional associations with apathy severity, particularly within white matter pathways involved in fronto-limbic, fronto-striatal, and higher-order associative processing. Although these associations were not fully explained by motor severity or dopaminergic medication burden, their attenuation after adjustment for affective and cognitive symptoms suggests partial overlap with broader neuropsychiatric mechanisms. (3) Glymphatic-related MRI metrics showed less consistent direct relationships with apathy; however, the significant coupling between choroid plexus volume and apathy-related FW abnormalities suggests that choroid plexus- and CSF-related homeostatic alterations may be linked to the extracellular white matter environment in which apathy develops. Taken together, these findings support an associative glymphatic–white matter framework for PD apathy, in which extracellular white matter abnormalities appear more closely related to the clinical expression of apathy, whereas glymphatic-related alterations may reflect a broader homeostatic context. Longitudinal studies are required to determine the temporal and causal relationships among these processes.

## Data Availability

The raw data supporting the conclusions of this article will be made available by the authors, without undue reservation.

## Footnotes

1
https://www.nitrc.org/projects/noddi_toolbox

2
https://dipy.org/
