# Supplementary material for: Relationship between apathy, glymphatic dysfunction, and white matter integrity in Parkinson’s disease: a multimodal MRI study
Source: Front Aging Neurosci. 2026 Oct 26;18:1939737. doi: 10.3389/fnagi.2026.1939737 (PMC13745724; doi:10.3389/fnagi.2026.1939737)
Supplement: Supplementary file 1 [file Data_Sheet_1.docx]

| Cluster | JHU White-Matter Tractography Atlas | JHU ICBM-DTI-81 White-Matter Labels | Side | Voxel | P-value | MNI coordinate X | MNI coordinate Y | MNI coordinate Z |
| --- | --- | --- | --- | --- | --- | --- | --- | --- |
| 1 | Superior longitudinal fasciculus | Unclassified | R | 2 | <0.050 | 21.5 | -49.5 | 58.5 |
| 2 | Unclassified | Unclassified | R | 5 | <0.050 | 20.4 | -50.4 | 61.4 |
| 3 | Forceps minor / Anterior thalamic radiation / Inferior fronto-occipital fasciculus | Unclassified | R | 5 | <0.050 | 15.6 | 56.8 | 5 |
| 4 | Superior longitudinal fasciculus | Unclassified | R | 17 | <0.050 | 24.4 | -46.9 | 57.4 |
| 5 | Forceps minor / Anterior thalamic radiation / Cingulum (cingulate gyrus) | Unclassified | R | 411 | <0.001 | 13.5 | 42.3 | 33.6 |
| 6 | Cingulum (cingulate gyrus) | Body of corpus callosum / Anterior corona radiata / Superior corona radiata | R | 475 | <0.001 | 17.3 | 3.25 | 34.5 |
| 7 | Forceps minor / Anterior thalamic radiation | Anterior corona radiata / Genu of corpus callosum | R | 606 | <0.001 | 20.1 | 36.8 | 18.5 |
| 8 | Corticospinal tract / Forceps major / Inferior fronto-occipital fasciculus / Superior longitudinal fasciculus | Posterior corona radiata / Superior corona radiata / Splenium of corpus callosum / Posterior thalamic radiation / Superior longitudinal fasciculus | R | 3152 | <0.001 | 22.1 | -40.1 | 35.3 |

Supplementary Table S1: Summary of fwFA TBSS differences between the PD and HC groups.

| Cluster | JHU White-Matter Tractography Atlas | JHU ICBM-DTI-81 White-Matter Labels | Side | Voxel | P-value | MNI coordinate X | MNI coordinate Y | MNI coordinate Z |
| --- | --- | --- | --- | --- | --- | --- | --- | --- |
| 1 | Anterior thalamic radiation / Corticospinal tract | Unclassified | R | 2 | <0.500 | 6 | -25.5 | -20.5 |
| 2 | Corticospinal tract | Corticospinal tract / Cerebral peduncle | R | 55 | <0.001 | 8.93 | -21.5 | -22.3 |
| 3 | Anterior thalamic radiation / Corticospinal tract | Superior cerebellar peduncle / Middle cerebellar peduncle | L | 417 | <0.001 | -4.55 | -27.1 | -17.9 |
| 4 | Superior longitudinal fasciculus / Inferior fronto-occipital fasciculus / Forceps major & minor / Cingulum / Anterior thalamic radiation / Corticospinal tract | Corpus callosum (genu, body, splenium) / Internal capsule / Corona radiata / Superior longitudinal fasciculus / Cingulum | Bilateral | 17895 | <0.001 | 0 | -20 | 20 |

Supplementary Table S2: Summary of fwAD TBSS differences between the PD and HC groups.

| Cluster | JHU White-Matter Tractography Atlas | JHU ICBM-DTI-81 White-Matter Labels | Side | Voxel | P-value | MNI coordinate X | MNI coordinate Y | MNI coordinate Z |
| --- | --- | --- | --- | --- | --- | --- | --- | --- |
| 1 | Unclassified | Unclassified | R | 1 | <0.050 | 17 | 30 | 38 |
| 2 | Unclassified | Unclassified | R | 14 | <0.050 | 19.9 | 25.4 | 35.5 |
| 3 | Forceps minor / Anterior thalamic radiation / Cingulum (cingulate gyrus) | Unclassified | R | 36 | <0.050 | 12.5 | 56 | 19.8 |
| 4 | Forceps minor / Cingulum (cingulate gyrus) | Unclassified | R | 55 | <0.050 | 11.5 | 46.7 | 34.7 |
| 5 | Forceps minor / Inferior fronto-occipital fasciculus / Anterior thalamic radiation | Unclassified | R | 65 | <0.050 | 17.1 | 39.4 | 30.4 |
| 6 | Cingulum (cingulate gyrus) | Cingulum (cingulate gyrus) / Body of corpus callosum | L | 97 | <0.001 | -7.63 | -0.877 | 32.6 |
| 7 | Cingulum (cingulate gyrus) | Cingulum (cingulate gyrus) | L | 118 | <0.001 | -10.4 | -30.1 | 33.1 |
| 8 | Superior longitudinal fasciculus / Inferior longitudinal fasciculus / Inferior fronto-occipital fasciculus / Forceps major | Posterior thalamic radiation / Splenium of corpus callosum / Superior longitudinal fasciculus | L | 3859 | <0.001 | -30.5 | -53.3 | 17 |
| 9 | Forceps minor / Anterior thalamic radiation / Uncinate fasciculus / Inferior fronto-occipital fasciculus | Anterior corona radiata / Superior corona radiata / Body of corpus callosum / Superior longitudinal fasciculus | L | 6122 | <0.001 | -22.3 | 20.1 | 27.7 |
| 10 | Superior longitudinal fasciculus / Inferior fronto-occipital fasciculus / Inferior longitudinal fasciculus / Forceps major / Corticospinal tract | Superior corona radiata / Anterior corona radiata / Posterior thalamic radiation / Splenium of corpus callosum / Superior longitudinal fasciculus | R | 9811 | <0.001 | 30 | -20.6 | 22.1 |

Supplementary Table S3: Summary of fwMD TBSS differences between the PD and HC groups.

| Cluster | JHU White-Matter Tractography Atlas | JHU ICBM-DTI-81 White-Matter Labels | Side | Voxel | P-value | MNI coordinate X | MNI coordinate Y | MNI coordinate Z |
| --- | --- | --- | --- | --- | --- | --- | --- | --- |
| 1 | Cingulum (cingulate gyrus) / Anterior thalamic radiation | Posterior corona radiata | L | 4 | <0.050 | -20 | -50.5 | 28.5 |
| 2 | Unclassified | Unclassified | L | 42 | <0.001 | -14.7 | 16.9 | 47.7 |
| 3 | Forceps minor / Cingulum (cingulate gyrus) / Anterior thalamic radiation | Anterior corona radiata | L | 55 | <0.050 | -19.2 | 40.1 | 8.2 |
| 4 | Superior longitudinal fasciculus / Superior longitudinal fasciculus (temporal part) | Unclassified | L | 63 | <0.001 | -39.2 | -47.5 | 33.1 |
| 5 | Inferior fronto-occipital fasciculus / Uncinate fasciculus / Anterior thalamic radiation | Anterior corona radiata | L | 125 | <0.001 | -29.6 | 35.3 | 2.22 |
| 6 | Superior longitudinal fasciculus / Superior longitudinal fasciculus (temporal part) | Posterior corona radiata | L | 292 | <0.001 | -29.6 | -53.1 | 28.2 |
| 7 | Forceps major / Inferior fronto-occipital fasciculus / Inferior longitudinal fasciculus / Anterior thalamic radiation | Posterior thalamic radiation | L | 438 | <0.001 | -27.4 | -71.9 | 21.5 |
| 8 | Forceps major / Inferior fronto-occipital fasciculus / Inferior longitudinal fasciculus | Splenium of corpus callosum / Posterior thalamic radiation | L | 885 | <0.001 | -26.1 | -54.2 | 7.15 |
| 9 | Forceps minor / Anterior thalamic radiation / Cingulum (cingulate gyrus) / Uncinate fasciculus | Anterior corona radiata / Superior corona radiata | L | 2237 | <0.001 | -20 | 27.7 | 28.2 |
| 10 | Corticospinal tract / Inferior fronto-occipital fasciculus / Forceps major & minor / Superior longitudinal fasciculus | Superior corona radiata / Anterior corona radiata / Posterior thalamic radiation / Splenium of corpus callosum / Superior longitudinal fasciculus | R | 12910 | <0.001 | 25.5 | -14.4 | 27.7 |

Supplementary Table S4: Summary of fwRD TBSS differences between the PD and HC groups.

| Cluster | JHU White-Matter Tractography Atlas | JHU ICBM-DTI-81 White-Matter Labels | Side | Voxel | P-value | MNI coordinate X | MNI coordinate Y | MNI coordinate Z |
| --- | --- | --- | --- | --- | --- | --- | --- | --- |
| 1 | Superior longitudinal fasciculus | Unclassified | R | 5 | <0.050 | 49.2 | -37.8 | 18.6 |
| 2 | Superior longitudinal fasciculus / Superior longitudinal fasciculus (temporal part) | Unclassified | R | 7 | <0.050 | 44.4 | -39.4 | 20.4 |
| 3 | Superior longitudinal fasciculus | Unclassified | R | 11 | <0.050 | 46.1 | -36.2 | 13.7 |
| 4 | Superior longitudinal fasciculus / Superior longitudinal fasciculus (temporal part) | Unclassified | R | 21 | <0.001 | 36.5 | 15.4 | 19 |
| 5 | Superior longitudinal fasciculus / Superior longitudinal fasciculus (temporal part) | Superior longitudinal fasciculus | R | 472 | <0.001 | 40.3 | 1.47 | 19.5 |
| 6 | Forceps minor / Anterior thalamic radiation / Corticospinal tract / Inferior fronto-occipital fasciculus / Superior longitudinal fasciculus / Uncinate fasciculus | Genu of corpus callosum / Body of corpus callosum / Retrolenticular part of internal capsule / Corona radiata / Superior longitudinal fasciculus | Bilateral | 17807 | <0.001 | -5.09 | -13.2 | 18.8 |

Supplementary Table S5: Summary of FW TBSS differences between the PD and HC groups.

| Cluster | JHU White-Matter Tractography Atlas | JHU ICBM-DTI-81 White-Matter Labels | Side | Voxel | P-value | MNI coordinate X | MNI coordinate Y | MNI coordinate Z |
| --- | --- | --- | --- | --- | --- | --- | --- | --- |
| 1 | Superior longitudinal fasciculus / Forceps minor & major / Inferior fronto-occipital fasciculus / Inferior longitudinal fasciculus / Anterior thalamic radiation / Corticospinal tract / Cingulum / Uncinate fasciculus | Corpus callosum (genu, body, splenium) / Internal capsule / Corona radiata / Posterior thalamic radiation / Superior longitudinal fasciculus / Cingulum | Bilateral | 42071 | <0.001 | 2.07 | -20.3 | 20.8 |

Supplementary Table S6: Summary of NDI TBSS differences between the PD and HC groups.

| Cluster | JHU White-Matter Tractography Atlas | JHU ICBM-DTI-81 White-Matter Labels | Side | Voxel | P-value | MNI coordinate X | MNI coordinate Y | MNI coordinate Z |
| --- | --- | --- | --- | --- | --- | --- | --- | --- |
| 1 | Inferior fronto-occipital fasciculus / Inferior longitudinal fasciculus | Unclassified | R | 14 | <0.050 | 36.1 | -75.1 | 17.1 |
| 2 | Unclassified | Unclassified | R | 21 | <0.001 | 37.7 | -67.5 | 25.3 |
| 3 | Cingulum (cingulate gyrus) / Cingulum (hippocampus) / Forceps major / Inferior fronto-occipital fasciculus | Splenium of corpus callosum / Cingulum (cingulate gyrus) | R | 647 | <0.001 | 14.1 | -61.1 | 33.6 |
| 4 | Inferior fronto-occipital fasciculus / Inferior longitudinal fasciculus / Forceps major | Posterior thalamic radiation | R | 1150 | <0.001 | 26.3 | -79.4 | -1.97 |
| 5 | Forceps minor / Corticospinal tract / Anterior thalamic radiation / Inferior fronto-occipital fasciculus / Uncinate fasciculus | Body of corpus callosum / Anterior corona radiata / Superior corona radiata | R | 3531 | <0.001 | 18.3 | -3.11 | 36.7 |

Supplementary Table S7: Summary of ODI TBSS differences between the PD and HC groups.

| Component | **Strong Loadings (≥0.3)** | Moderate Loadings (0.1-0.3) |
| --- | --- | --- |
| SPC1_All | fwRDcluster10 (0.862) | fwRDcluster9 (0.289), fwRDcluster1 (0.276), fwFAcluster7 (-0.168), fwRDcluster7 (0.122), fwRDcluster4 (0.102) |
| SPC2_All | fwADcluster3 (-0.588), fwADcluster1 (-0.546), fwADcluster2 (-0.509) | fwADcluster4 (-0.169), fwRDcluster4 (-0.143), fwMDcluster7 (-0.137) |
| SPC3_All | ODIcluster5 (-0.470), ODIcluster1 (-0.463), ODIcluster2 (-0.405), ODIcluster4 (-0.402) | fwFAcluster3 (0.296), fwFAcluster8 (0.289), fwFAcluster4 (0.159), fwRDcluster7 (-0.116), fwMDcluster4 (0.107) |
| SPC4_All | fwFAcluster4 (0.679), fwMDcluster1 (-0.349), fwMDcluster5 (-0.329), fwFAcluster6 (-0.301) | fwMDcluster4 (-0.289), fwFAcluster7 (-0.237), fwFAcluster5 (0.154), ODIcluster5 (0.138), fwRDcluster4 (-0.105), fwRDcluster5 (0.105) |
| SPC5_All | FWcluster5 (-0.485), FWcluster6 (-0.480), fwMDcluster3 (-0.441) | fwMDcluster6 (-0.286), fwMDcluster9 (-0.246), fwRDcluster8 (-0.200), fwRDcluster3 (-0.163), fwRDcluster5 (-0.160), ODIcluster1 (-0.144), fwFAcluster3 (-0.142), fwADcluster4 (-0.131), fwMDcluster4 (-0.123), fwFAcluster6 (0.122), fwADcluster1 (0.104) |

Supplementary Table S8. Components of each SPC from SPCA analysis of TBSS differences in the whole cohort (|loading| ≥ 0.1).

Total variance explained = 53.94%, average sparsity = 54.3%, n = 79 (PD + HC).

fwRD, free water‑corrected radial diffusivity; fwAD, free water‑corrected axial diffusivity; ODI, orientation dispersion index; fwFA, free water‑corrected fractional anisotropy; fwMD, free water‑corrected mean diffusivity; FW, free water.

| Component | **Strong Loadings (≥0.3)** | Moderate Loadings (0.1-0.3) |
| --- | --- | --- |
| SPC1_PD | fwMDclu1 (0.415), fwMDclu5 (0.374), fwRDclu10 (0.328), fwMDclu8 (0.327), fwMDclu2 (0.321) | fwMDclu4 (0.289), fwMDclu10 (0.287), fwFAclu5 (-0.280), fwMDclu9 (0.212), fwRDclu4 (0.208), fwRDclu1 (0.186) |
| SPC2_PD | fwFAclu7 (-0.675), fwRDclu3 (0.419), fwRDclu5 (0.339) | fwFAclu6 (-0.277), fwRDclu1 (0.231), fwFAclu8 (-0.188), fwMDclu7 (0.180), fwRDclu6 (0.149), NDIclu (-0.105), fwRDclu7 (0.100) |
| SPC3_PD | FWclu6 (**-0.647**), fwMDclu3 (**-0.442**) | ODIclu1 (-0.377), fwRDclu8 (-0.291), fwADclu4 (-0.252), fwMDclu7 (-0.193), fwMDclu6 (-0.171), fwFAclu8 (0.146) |

Supplementary Table S9. Components of each SPC from SPCA analysis of TBSS differences in the PD group (|loading| ≥ 0.1).

Total variance explained = 53.94%, average sparsity = 54.3%, n = 79 (PD + HC).

fwMD, free water‑corrected mean diffusivity; fwRD, free water‑corrected radial diffusivity; fwFA, free water‑corrected fractional anisotropy; FW, free water; ODI, orientation dispersion index; fwAD, free water‑corrected axial diffusivity; NDI, neurite density index; CST, corticospinal tract; IFOF, inferior fronto‑occipital fasciculus; ATR, anterior thalamic radiation; WM, white matter.

| Direction | Cluster | JHU White-Matter Tractography Atlas | JHU ICBM-DTI-81 White-Matter Labels | Side | Voxel | P-value | MNI coordinate X | MNI coordinate Y | MNI coordinate Z |
| --- | --- | --- | --- | --- | --- | --- | --- | --- | --- |
| HC > APD | 1 | — | Unclassified | — | 30 | <0.001 | 0.204 | -15.8 | -20.4 |
|  | 2 | — | Unclassified | — | 41 | <0.001 | 0.049 | 3.5 | 15.4 |
|  | 3 | Corticospinal tract / Anterior thalamic radiation | Cerebral peduncle | L | 50 | <0.001 | -9.14 | -13.3 | -16.2 |
|  | 4 | Corticospinal tract / Anterior thalamic radiation | Cerebral peduncle | L | 63 | <0.001 | -11.7 | -24.7 | -26.7 |
|  | 5 | Anterior thalamic radiation | Superior cerebellar peduncle | L | 72 | <0.001 | -6.33 | -42.7 | -26.7 |
|  | 6 | Anterior thalamic radiation | Superior cerebellar peduncle | R | 106 | <0.001 | 6.45 | -41.6 | -25.8 |
|  | 7 | Corticospinal tract / Anterior thalamic radiation | Cerebral peduncle | R | 251 | <0.001 | 11.6 | -19.6 | -16 |
|  | 8 | Forceps minor / Forceps major / Anterior thalamic radiation / Cingulum | Genu of corpus callosum / Body of corpus callosum / Splenium of corpus callosum / Corona radiata | Bilateral | 10086 | <0.001 | 0.106 | -16.5 | 14.9 |
| APD > HC | 9-43 | Various small clusters (corticospinal tract, cingulum, etc.) | Unclassified / Various | Bilateral | 1-24 | <0.050 | — | — | — |
|  | 45 | Anterior thalamic radiation | Unclassified | L | 102 | <0.001 | -8.6 | -11.6 | 5.64 |
|  | 45 | Anterior thalamic radiation / Corticospinal tract / Superior longitudinal fasciculus / Inferior fronto-occipital fasciculus / Inferior longitudinal fasciculus / Uncinate fasciculus | Internal capsule / Corona radiata / Cerebral peduncle / Cerebellar peduncles / Superior longitudinal fasciculus | Bilateral | 103266 | <0.001 | 1.26 | -23.2 | 12.3 |

Supplementary Table S10: Summary of fiso TBSS differences between the APD and HC groups.

| Cluster | JHU White-Matter Tractography Atlas | JHU ICBM-DTI-81 White-Matter Labels | Side | Voxel | P-value | MNI coordinate X | MNI coordinate Y | MNI coordinate Z |
| --- | --- | --- | --- | --- | --- | --- | --- | --- |
| 1 | Anterior thalamic radiation / Corticospinal tract / Superior longitudinal fasciculus | Unclassified | R | 9 | <0.050 | -22 | -44.7 | 49.6 |
| 2 | Cingulum (cingulate gyrus) / Cingulum (hippocampus) / Forceps major / Inferior fronto-occipital fasciculus / Inferior longitudinal fasciculus / Superior longitudinal fasciculus | Body of corpus callosum / Splenium of corpus callosum / Posterior thalamic radiation / Posterior corona radiata / Cingulum / Superior longitudinal fasciculus | L | 4650 | <0.001 | -26.8 | -42.2 | 17.9 |
| 3 | Forceps minor / Anterior thalamic radiation / Inferior fronto-occipital fasciculus / Uncinate fasciculus / Superior longitudinal fasciculus | Genu of corpus callosum / Anterior limb of internal capsule / Posterior limb of internal capsule / Anterior Lcorona radiata / Superior corona radiata / Inferior fronto-occipital fasciculus | L | 5145 | <0.001 | -21.7 | 12.8 | 16.4 |
| 4 | Corticospinal tract / Anterior thalamic radiation / Inferior fronto-occipital fasciculus / Inferior longitudinal fasciculus / Superior longitudinal fasciculus / Uncinate fasciculus | Posterior limb of internal capsule / Anterior corona radiata / Superior corona radiata / Posterior thalamic radiation / Superior longitudinal fasciculus | L | 13911 | <0.001 | 22.2 | -16.9 | 6.11 |

Supplementary Table S11: Summary of fwAD TBSS differences between the APD and HC groups.

| Cluster | JHU White-Matter Tractography Atlas | JHU ICBM-DTI-81 White-Matter Labels | Side | Voxel | P-value | MNI coordinate X | MNI coordinate Y | MNI coordinate Z |
| --- | --- | --- | --- | --- | --- | --- | --- | --- |
| 1 | Forceps minor / Anterior thalamic radiation | Unclassified | R | 3 | <0.050 | 19.3 | 33.3 | 23.7 |
| 2 | Corticospinal tract | Unclassified | R | 6 | <0.050 | 15.2 | -32.3 | 55.7 |
| 3 | Corticospinal tract | Unclassified | R | 64 | <0.001 | 18.9 | -19.6 | 55.6 |
| 4 | Forceps minor / Anterior thalamic radiation / Inferior fronto-occipital fasciculus | Anterior corona radiata | R | 127 | <0.001 | 21.2 | 39.7 | 16.6 |

Supplementary Table S12: Summary of fwFA TBSS differences between the APD and HC groups.

| Cluster | JHU White-Matter Tractography Atlas | JHU ICBM-DTI-81 White-Matter Labels | Side | Voxel | P-value | MNI coordinate X | MNI coordinate Y | MNI coordinate Z |
| --- | --- | --- | --- | --- | --- | --- | --- | --- |
| 1 | Forceps minor & major / Anterior thalamic radiation / Corticospinal tract / Cingulum / Inferior fronto-occipital fasciculus / Inferior longitudinal fasciculus / Superior longitudinal fasciculus / Uncinate fasciculus | Genu of corpus callosum / Body of corpus callosum / Splenium of corpus callosum / Internal capsule / Corona radiata / Posterior thalamic radiation / External capsule / Superior longitudinal fasciculus / Cingulum | Bilateral | 34302 | <0.001 | 3.79 | -7.38 | 17.8 |

Supplementary Table S13: Summary of fwMD TBSS differences between the APD and HC groups.

| Cluster | JHU White-Matter Tractography Atlas | JHU ICBM-DTI-81 White-Matter Labels | Side | Voxel | P-value | MNI coordinate X | MNI coordinate Y | MNI coordinate Z |
| --- | --- | --- | --- | --- | --- | --- | --- | --- |
| 1 | — | Unclassified | R | 37 | <0.050 | 37.6 | -68.4 | 28 |
| 2 | Forceps minor & major / Anterior thalamic radiation / Corticospinal tract / Inferior fronto-occipital fasciculus / Superior longitudinal fasciculus | Body of corpus callosum / Splenium of corpus callosum / Corona radiata / Posterior thalamic radiation / Superior longitudinal fasciculus | R | 8096 | <0.001 | 22.9 | -0.422 | 29.1 |

Supplementary Table S14: Summary of fwRD TBSS differences between the APD and HC groups.

| Cluster | JHU White-Matter Tractography Atlas | JHU ICBM-DTI-81 White-Matter Labels | Side | Voxel | P-value | MNI coordinate X | MNI coordinate Y | MNI coordinate Z |
| --- | --- | --- | --- | --- | --- | --- | --- | --- |
| 1 | Forceps minor & major / Anterior thalamic radiation / Corticospinal tract / Inferior fronto-occipital fasciculus / Inferior longitudinal fasciculus / Superior longitudinal fasciculus / Uncinate fasciculus | Genu of corpus callosum / Body of corpus callosum / Splenium of corpus callosum / Internal capsule / Corona radiata / Posterior thalamic radiation / External capsule / Superior longitudinal fasciculus / Fornix | Bilateral | 39758 | <0.001 | -0.23 | -11.3 | 16.7 |

Supplementary Table S15: Summary of FW TBSS differences between the APD and HC groups.

| Cluster | JHU White-Matter Tractography Atlas | JHU ICBM-DTI-81 White-Matter Labels | Side | Voxel | P-value | MNI coordinate X | MNI coordinate Y | MNI coordinate Z |
| --- | --- | --- | --- | --- | --- | --- | --- | --- |
| 1 | Forceps minor & major / Anterior thalamic radiation / Corticospinal tract / Cingulum / Inferior fronto-occipital fasciculus / Inferior longitudinal fasciculus / Superior longitudinal fasciculus / Uncinate fasciculus | Genu of corpus callosum / Body of corpus callosum / Splenium of corpus callosum / Internal capsule / Corona radiata / Posterior thalamic radiation / External capsule / Superior longitudinal fasciculus / Cingulum / Fornix | Bilateral | 44110 | <0.001 | 2.92 | -12.2 | 18.1 |

Supplementary Table S16: Summary of NDI TBSS differences between the APD and HC groups.

| Cluster | JHU White-Matter Tractography Atlas | JHU ICBM-DTI-81 White-Matter Labels | Side | Voxel | P-value | MNI coordinate X | MNI coordinate Y | MNI coordinate Z |
| --- | --- | --- | --- | --- | --- | --- | --- | --- |
| 1 | Inferior fronto-occipital fasciculus / Inferior longitudinal fasciculus | Unclassified | R | 52 | <0.050 | 41.9 | -66.3 | -6.62 |
| 2 | Corticospinal tract | Unclassified | R | 55 | <0.050 | 19.5 | -20.7 | 54.9 |
| 3 | Forceps minor / Anterior thalamic radiation / Inferior fronto-occipital fasciculus | Unclassified | R | 102 | <0.001 | 21.7 | 43.3 | 16.1 |
| 4 | Corticospinal tract / Superior longitudinal fasciculus | Body of corpus callosum / Superior corona radiata / Posterior corona radiata | R | 178 | <0.001 | 21.4 | -28.3 | 40 |
| 5 | Forceps major / Inferior fronto-occipital fasciculus / Inferior longitudinal fasciculus | Posterior thalamic radiation | R | 600 | <0.001 | 22.8 | -81.8 | 1.82 |

Supplementary Table S17: Summary of ODI TBSS differences between the APD and HC groups.

| Direction | Cluster | JHU White-Matter Tractography Atlas | JHU ICBM-DTI-81 White-Matter Labels | Side | Voxel | P-value | MNI coordinate X | MNI coordinate Y | MNI coordinate Z |
| --- | --- | --- | --- | --- | --- | --- | --- | --- | --- |
| HC > APD | 1 | — | Unclassified | — | 1 | <0.001 | -18 | -37 | 14 |
|  | 2 | — | Unclassified | — | 2 | <0.001 | -6.5 | -37 | -9 |
|  | 3 | Corticospinal tract / Anterior thalamic radiation | Cerebral peduncle | R | 4 | <0.001 | 12 | -25.5 | -15.5 |
|  | 4 | Anterior thalamic radiation | Superior cerebellar peduncle | R | 5 | <0.001 | -5 | -40.8 | -24.2 |
|  | 5 | Anterior thalamic radiation | Unclassified | L | 31 | <0.050 | 22.1 | -37.5 | 13.3 |
|  | 6 | Anterior thalamic radiation | Superior cerebellar peduncle | R | 58 | <0.050 | 6.07 | -42.1 | -25.5 |
|  | 7 | — | Unclassified | — | 76 | <0.050 | 0.459 | 7.29 | 10.6 |
|  | 8 | Forceps minor & major / Anterior thalamic radiation / Cingulum / Inferior fronto-occipital fasciculus / Inferior longitudinal fasciculus | Genu of corpus callosum / Body of corpus callosum / Splenium of corpus callosum / Corona radiata / Posterior thalamic radiation | Bilateral | 9826 | <0.050 | 0.004 | -18.5 | 16.4 |
| APD > HC | 9-30 | Various small clusters | Unclassified / Various | Bilateral | 1-17 | <0.05 | — | — | — |
|  | 31 | Superior longitudinal fasciculus / Superior longitudinal fasciculus (temporal part) | External capsule | R | 23 | <0.001 | 35 | -2.22 | 5.95 |
|  | 32 | Cingulum (hippocampus) | Unclassified | L | 29 | <0.001 | -28.2 | -31.9 | -15.5 |
|  | 33 | Anterior thalamic radiation | Unclassified | R | 120 | <0.001 | 9.95 | -9.99 | 7.21 |
|  | 34 | Inferior longitudinal fasciculus | Unclassified | R | 214 | <0.001 | 40.5 | -72.8 | 6.98 |
|  | 35 | Superior longitudinal fasciculus / Inferior fronto-occipital fasciculus / Inferior longitudinal fasciculus / Uncinate fasciculus / Forceps minor & major / Corticospinal tract / Cingulum | Superior corona radiata / Anterior corona radiata / Internal capsule / Cingulum / Superior longitudinal fasciculus | R | 39622 | <0.001 | 29.4 | -19.9 | 23.1 |
|  | 36 | Superior longitudinal fasciculus / Inferior fronto-occipital fasciculus / Inferior longitudinal fasciculus / Uncinate fasciculus / Forceps minor / Corticospinal tract | Corona radiata / Internal capsule / Superior longitudinal fasciculus | L | 54962 | <0.001 | -21.6 | -28.8 | 7.5 |

Supplementary Table S18: Summary of fiso TBSS differences between the nAPD and HC groups.

| Cluster | JHU White-Matter Tractography Atlas | JHU ICBM-DTI-81 White-Matter Labels | Side | Voxel | P-value | MNI coordinate X | MNI coordinate Y | MNI coordinate Z |
| --- | --- | --- | --- | --- | --- | --- | --- | --- |
| 1 | Inferior longitudinal fasciculus / Inferior fronto-occipital fasciculus | Unclassified | R | 1 | <0.050 | 36 | -73 | 17 |
| 2 | Anterior thalamic radiation / Inferior fronto-occipital fasciculus / Inferior longitudinal fasciculus / Superior longitudinal fasciculus | Posterior corona radiata / Posterior thalamic radiation | L | 69 | <0.050 | -27.8 | -45.6 | 20.9 |
| 3 | Superior longitudinal fasciculus / Superior longitudinal fasciculus (temporal part) / Corticospinal tract / Anterior thalamic radiation | Superior corona radiata / External capsule / Superior longitudinal fasciculus | R | 894 | <0.001 | 34.2 | 3.4 | 20.7 |
| 4 | Superior longitudinal fasciculus / Forceps major / Inferior fronto-occipital fasciculus / Inferior longitudinal fasciculus / Superior longitudinal fasciculus (temporal part) / Corticospinal tract | Body of corpus callosum / Splenium of corpus callosum / Posterior corona radiata / Posterior thalamic radiation / Superior longitudinal fasciculus / Tapetum | Bilateral | 7233 | <0.001 | 4.12 | -47.1 | 24.2 |

Supplementary Table S19: Summary of NDI TBSS differences between the nAPD and HC groups.

|  | Core | | | Core + SAS + SDS | | | Core + MoCA | | | ALL | | |
| --- | --- | --- | --- | --- | --- | --- | --- | --- | --- | --- | --- | --- |
|  | *r* | *p* | *VIF_max* | *r* | *p* | *VIF_max* | *r* | *p* | *VIF_max* | *r* | *p* | *VIF_max* |
| PVS_WM | -0.199 | 0.226 | 1.008 | -0.250 | 0.031* | 3.103 | -0.098 | 0.554 | 1.080 | -0.174 | 0.295 | 3.211 |
| PVS_SUB | -0.093 | 0.660 | 1.008 | -0.235 | 0.042* | 3.103 | -0.146 | 0.528 | 1.080 | -0.244 | 0.036* | 3.211 |
| pPVS | -0.145 | 0.384 | 1.008 | -0.232 | 0.045* | 3.103 | -0.087 | 0.554 | 1.080 | -0.177 | 0.295 | 3.211 |
| CPV | 0.263 | 0.021* | 1.008 | 0.219 | 0.127 | 3.103 | 0.093 | 0.554 | 1.080 | 0.118 | 0.385 | 3.211 |
| CPV_right | 0.258 | 0.023* | 1.008 | 0.211 | 0.127 | 3.103 | 0.048 | 0.750 | 1.080 | 0.084 | 0.477 | 3.211 |
| CPV_left | 0.232 | 0.042* | 1.008 | 0.193 | 0.153 | 3.103 | 0.121 | 0.546 | 1.080 | 0.130 | 0.372 | 3.211 |
| ALPS_left | 0.019 | 0.883 | 1.008 | 0.028 | 0.814 | 3.103 | 0.136 | 0.528 | 1.080 | 0.107 | 0.401 | 3.211 |
| ALPS_right | 0.028 | 0.883 | 1.008 | 0.133 | 0.310 | 3.103 | 0.172 | 0.508 | 1.080 | 0.212 | 0.257 | 3.211 |
| ALPS | 0.017 | 0.883 | 1.008 | 0.065 | 0.639 | 3.103 | 0.175 | 0.508 | 1.080 | 0.165 | 0.295 | 3.211 |
| negativecoupling | 0.053 | 0.883 | 1.008 | 0.180 | 0.167 | 3.103 | 0.030 | 0.800 | 1.080 | 0.140 | 0.367 | 3.211 |
| Positivecouplig | -0.156 | 0.384 | 1.008 | -0.326 | 0.047 | 3.103 | -0.224 | 0.508 | 1.080 | -0.342 | 0.032 | 3.211 |

Supplementary Table S20. Partial correlations between glymphatic dysfunction metrics and apathy severity (AES) in the whole cohort (PD + HC, n = 79) after regressing out different sets of covariates.

Four adjustment schemes are shown: Core (age and sex), Core + SAS + SDS (age, sex, SAS, and SDS), Core + MoCA (age, sex, and MoCA), and ALL (age, sex, SAS, SDS, and MoCA). Values are partial correlation coefficients (r) and corresponding p values. VIF max indicates the maximum variance inflation factor among covariates within each scheme. * indicates p‑values before FDR correction.

AES, Apathy Evaluation Scale; SAS, Self-Rating Anxiety Scale; SDS, Self-Rating Depression Scale; MoCA, Montreal Cognitive Assessment; CPV, choroid plexus volume; CPV_left, left hemisphere CPV; CPV_right, right hemisphere CPV; PVS, perivascular space volume; PVS_SUB, PVS in subcortical gray matter nuclei; PVS_WM, PVS in the white matter region; pPVS, whole-brain PVS; ALPS, index of diffusion along perivascular spaces; ALPS_left, left hemisphere ALPS; ALPS_right, right hemisphere ALPS.

|  | Core | | | Core + SAS + SDS | | | Core + MoCA | | | Core + LEDD + UPDRS-III | | | ALL | | |
| --- | --- | --- | --- | --- | --- | --- | --- | --- | --- | --- | --- | --- | --- | --- | --- |
|  | *r* | *p* | *VIF_max* | *r* | *p* | *VIF_max* | *r* | *p* | *VIF_max* | *r* | *p* | *VIF_max* | *r* | *p* | *VIF_max* |
| PVS_WM | -0.191 | 0.486 | 1.135 | -0.183 | 0.449 | 2.562 | -0.044 | 0.798 | 1.156 | -0.227 | 0.426 | 1.399 | -0.046 | 0.845 | 3.008 |
| PVS_SUB | -0.284 | 0.379 | 1.135 | -0.282 | 0.436 | 2.562 | -0.264 | 0.579 | 1.156 | -0.278 | 0.401 | 1.399 | -0.131 | 0.845 | 3.008 |
| pPVS | -0.176 | 0.486 | 1.135 | -0.156 | 0.499 | 2.562 | -0.053 | 0.798 | 1.156 | -0.190 | 0.426 | 1.399 | 0.035 | 0.845 | 3.008 |
| CPV | 0.268 | 0.379 | 1.135 | 0.183 | 0.449 | 2.562 | 0.180 | 0.579 | 1.156 | 0.271 | 0.401 | 1.399 | 0.112 | 0.845 | 3.008 |
| CPV_right | 0.302 | 0.379 | 1.135 | 0.218 | 0.449 | 2.562 | 0.175 | 0.579 | 1.156 | 0.309 | 0.401 | 1.399 | 0.127 | 0.845 | 3.008 |
| CPV_left | 0.200 | 0.486 | 1.135 | 0.123 | 0.580 | 2.562 | 0.170 | 0.579 | 1.156 | 0.202 | 0.426 | 1.399 | 0.086 | 0.845 | 3.008 |
| ALPS_left | 0.045 | 0.787 | 1.135 | 0.103 | 0.606 | 2.562 | 0.097 | 0.773 | 1.156 | 0.043 | 0.804 | 1.399 | 0.077 | 0.845 | 3.008 |
| ALPS_right | 0.152 | 0.486 | 1.135 | 0.267 | 0.436 | 2.562 | 0.228 | 0.579 | 1.156 | 0.181 | 0.426 | 1.399 | 0.352 | 0.045* | 3.008 |
| ALPS | 0.104 | 0.589 | 1.135 | 0.196 | 0.449 | 2.562 | 0.173 | 0.579 | 1.156 | 0.116 | 0.549 | 1.399 | 0.222 | 0.845 | 3.008 |
| negativecoupling | 0.141 | 0.486 | 1.135 | 0.265 | 0.436 | 2.562 | 0.081 | 0.773 | 1.156 | 0.135 | 0.528 | 1.399 | 0.146 | 0.845 | 3.008 |
| Positivecoupling | 0.169 | 0.486 | 1.135 | 0.087 | 0.616 | 2.562 | 0.098 | 0.773 | 1.156 | 0.174 | 0.426 | 1.399 | 0.070 | 0.845 | 3.008 |

Supplementary Table S21. Partial correlations between glymphatic dysfunction metrics and apathy severity (AES) in the PD cohort after regressing out different sets of covariates.

Five adjustment schemes are shown: Core (age, sex and disease duration), Core + SAS + SDS (age, sex, disease duration, SAS, and SDS), Core + MoCA (age, sex, disease duration, and MoCA), Core + LEDD + UPDRS-III (age, sex, disease duration, LEDD and UPDRS-III) and ALL (age, sex, disease duration, LEDD, UPDRS-III, SAS, SDS, and MoCA). Values are partial correlation coefficients (r) and corresponding p values. VIF max indicates the maximum variance inflation factor among covariates within each scheme. * indicates p‑values before FDR correction.

AES, Apathy Evaluation Scale; SAS, Self-Rating Anxiety Scale; SDS, Self-Rating Depression Scale; MoCA, Montreal Cognitive Assessment; LEDD, Levodopa Equivalent Daily Dose; UPDRS-III, Unified Parkinson’s Disease Rating Scale Part III; CPV, choroid plexus volume; CPV_left, left hemisphere CPV; CPV_right, right hemisphere CPV; PVS, perivascular space volume; PVS_SUB, PVS in subcortical gray matter nuclei; PVS_WM, PVS in the white matter region; pPVS, whole-brain PVS; ALPS, index of diffusion along perivascular spaces; ALPS_left, left hemisphere ALPS; ALPS_right, right hemisphere ALPS.

|  | Core | | | Core + SAS + SDS | | | Core + MoCA | | | ALL | | |
| --- | --- | --- | --- | --- | --- | --- | --- | --- | --- | --- | --- | --- |
|  | *r* | *p* | *VIF_max* | *r* | *p* | *VIF_max* | *r* | *p* | *VIF_max* | *r* | *p* | *VIF_max* |
| PVS_WM | -0.247 | 0.702 | 1.048 | -0.286 | 0.370 | 2.260 | -0.212 | 0.709 | 1.170 | -0.219 | 0.605 | 2.574 |
| PVS_SUB | 0.100 | 0.775 | 1.048 | -0.183 | 0.549 | 2.260 | -0.126 | 0.789 | 1.170 | -0.236 | 0.605 | 2.574 |
| pPVS | -0.142 | 0.750 | 1.048 | -0.290 | 0.370 | 2.260 | -0.197 | 0.709 | 1.170 | -0.251 | 0.605 | 2.574 |
| CPV | 0.154 | 0.750 | 1.048 | 0.206 | 0.531 | 2.260 | 0.005 | 0.976 | 1.170 | 0.068 | 0.861 | 2.574 |
| CPV_right | 0.049 | 0.853 | 1.048 | 0.101 | 0.735 | 2.260 | -0.089 | 0.789 | 1.170 | -0.050 | 0.861 | 2.574 |
| CPV_left | 0.194 | 0.704 | 1.048 | 0.243 | 0.456 | 2.260 | 0.074 | 0.789 | 1.170 | 0.143 | 0.702 | 2.574 |
| ALPS_left | 0.223 | 0.702 | 1.048 | 0.088 | 0.735 | 2.260 | 0.234 | 0.709 | 1.170 | 0.174 | 0.702 | 2.574 |
| ALPS_right | -0.013 | 0.941 | 1.048 | -0.076 | 0.735 | 2.260 | -0.087 | 0.789 | 1.170 | -0.119 | 0.702 | 2.574 |
| ALPS | 0.082 | 0.775 | 1.048 | -0.004 | 0.980 | 2.260 | 0.063 | 0.789 | 1.170 | 0.018 | 0.923 | 2.574 |
| negativecoupling | 0.095 | 0.775 | 1.048 | 0.135 | 0.701 | 2.260 | 0.120 | 0.789 | 1.170 | 0.130 | 0.702 | 2.574 |
| Positivecouplig | -0.342 | 0.041* | 1.048 | -0.448 | 0.008* | 2.260 | -0.361 | 0.033* | 1.170 | -0.398 | 0.022* | 2.574 |

Supplementary Table S22. Partial correlations between glymphatic dysfunction metrics and apathy severity (AES) in the HC cohort after regressing out different sets of covariates.

Four adjustment schemes are shown: Core (age and sex), Core + SAS + SDS (age, sex, SAS, and SDS), Core + MoCA (age, sex, and MoCA), and ALL (age, sex, SAS, SDS, and MoCA). Values are partial correlation coefficients (r) and corresponding p values. VIF max indicates the maximum variance inflation factor among covariates within each scheme. * indicates p‑values before FDR correction.

AES, Apathy Evaluation Scale; SAS, Self-Rating Anxiety Scale; SDS, Self-Rating Depression Scale; MoCA, Montreal Cognitive Assessment; CPV, choroid plexus volume; CPV_left, left hemisphere CPV; CPV_right, right hemisphere CPV; PVS, perivascular space volume; PVS_SUB, PVS in subcortical gray matter nuclei; PVS_WM, PVS in the white matter region; pPVS, whole-brain PVS; ALPS, index of diffusion along perivascular spaces; ALPS_left, left hemisphere ALPS; ALPS_right, right hemisphere ALPS.

|  | Core | | | Core + SAS + SDS | | | Core + MoCA | | | Core + LEDD + UPDRS-III | | | ALL | | |
| --- | --- | --- | --- | --- | --- | --- | --- | --- | --- | --- | --- | --- | --- | --- | --- |
|  | *r* | *p* | *VIF_max* | *r* | *p* | *VIF_max* | *r* | *p* | *VIF_max* | *r* | *p* | *VIF_max* | *r* | *p* | *VIF_max* |
| PVS_WM | -0.267 | 0.978 | 1.181 | -0.109 | 0.967 | 2.276 | -0.188 | 0.972 | 1.516 | -0.388 | 0.875 | 1.360 | -0.214 | 0.975 | 3.033 |
| PVS_SUB | -0.073 | 0.978 | 1.181 | 0.025 | 0.967 | 2.276 | -0.104 | 0.972 | 1.516 | -0.182 | 0.875 | 1.360 | 0.011 | 0.975 | 3.033 |
| pPVS | -0.039 | 0.978 | 1.181 | 0.066 | 0.967 | 2.276 | 0.010 | 0.972 | 1.516 | -0.135 | 0.875 | 1.360 | 0.095 | 0.975 | 3.033 |
| CPV | 0.142 | 0.978 | 1.181 | -0.053 | 0.967 | 2.276 | 0.114 | 0.972 | 1.516 | 0.077 | 0.875 | 1.360 | -0.062 | 0.975 | 3.033 |
| CPV_right | 0.335 | 0.978 | 1.181 | 0.040 | 0.967 | 2.276 | 0.260 | 0.972 | 1.516 | 0.199 | 0.875 | 1.360 | -0.032 | 0.975 | 3.033 |
| CPV_left | -0.023 | 0.978 | 1.181 | -0.058 | 0.967 | 2.276 | -0.045 | 0.972 | 1.516 | 0.051 | 0.875 | 1.360 | -0.040 | 0.975 | 3.033 |
| ALPS_left | -0.200 | 0.978 | 1.181 | 0.104 | 0.967 | 2.276 | -0.189 | 0.972 | 1.516 | -0.046 | 0.875 | 1.360 | 0.147 | 0.975 | 3.033 |
| ALPS_right | 0.007 | 0.978 | 1.181 | 0.155 | 0.967 | 2.276 | 0.085 | 0.972 | 1.516 | 0.197 | 0.875 | 1.360 | 0.308 | 0.975 | 3.033 |
| ALPS | -0.073 | 0.978 | 1.181 | 0.116 | 0.967 | 2.276 | -0.024 | 0.972 | 1.516 | 0.093 | 0.875 | 1.360 | 0.217 | 0.975 | 3.033 |
| negativecoupling | -0.257 | 0.978 | 1.181 | 0.012 | 0.967 | 2.276 | -0.310 | 0.972 | 1.516 | -0.290 | 0.875 | 1.360 | -0.106 | 0.975 | 3.033 |
| Positivecoupling | 0.217 | 0.978 | 1.181 | -0.093 | 0.967 | 2.276 | 0.058 | 0.972 | 1.516 | 0.213 | 0.875 | 1.360 | -0.168 | 0.975 | 3.033 |

Supplementary Table S23. Correlation results between glymphatic dysfunction metrics and apathy severity after regressing out different sets of covariates in the APD group.

Five adjustment schemes are shown: Core (age, sex and disease duration), Core + SAS + SDS (age, sex, disease duration, SAS, and SDS), Core + MoCA (age, sex, disease duration, and MoCA), Core + LEDD + UPDRS-III (age, sex, disease duration, LEDD and UPDRS-III) and ALL (age, sex, disease duration, LEDD, UPDRS-III, SAS, SDS, and MoCA). Values are partial correlation coefficients (r) and corresponding p values. VIF max indicates the maximum variance inflation factor among covariates within each scheme. * indicates p‑values before FDR correction.

AES, Apathy Evaluation Scale; SAS, Self-Rating Anxiety Scale; SDS, Self-Rating Depression Scale; MoCA, Montreal Cognitive Assessment; LEDD, Levodopa Equivalent Daily Dose; UPDRS-III, Unified Parkinson’s Disease Rating Scale Part III; CPV, choroid plexus volume; CPV_left, left hemisphere CPV; CPV_right, right hemisphere CPV; PVS, perivascular space volume; PVS_SUB, PVS in subcortical gray matter nuclei; PVS_WM, PVS in the white matter region; pPVS, whole-brain PVS; ALPS, index of diffusion along perivascular spaces; ALPS_left, left hemisphere ALPS; ALPS_right, right hemisphere ALPS.

|  | Core | | | Core + SAS + SDS | | | Core + MoCA | | | Core + LEDD + UPDRS-III | | | ALL | | |
| --- | --- | --- | --- | --- | --- | --- | --- | --- | --- | --- | --- | --- | --- | --- | --- |
|  | *r* | *p* | *VIF_max* | *r* | *p* | *VIF_max* | *r* | *p* | *VIF_max* | *r* | *p* | *VIF_max* | *r* | *p* | *VIF_max* |
| PVS_WM | -0.204 | 0.786 | 1.084 | -0.230 | 0.756 | 3.308 | -0.193 | 0.886 | 1.328 | -0.225 | 0.841 | 2.696 | -0.208 | 0.918 | 4.307 |
| PVS_SUB | -0.470 | 0.042* | 1.084 | -0.508 | 0.037* | 3.308 | -0.312 | 0.886 | 1.328 | -0.466 | 0.651 | 2.696 | -0.356 | 0.918 | 4.307 |
| pPVS | -0.278 | 0.786 | 1.084 | -0.336 | 0.756 | 3.308 | -0.163 | 0.886 | 1.328 | -0.284 | 0.841 | 2.696 | -0.205 | 0.918 | 4.307 |
| CPV | 0.193 | 0.786 | 1.084 | 0.205 | 0.756 | 3.308 | 0.124 | 0.886 | 1.328 | 0.189 | 0.841 | 2.696 | 0.136 | 0.919 | 4.307 |
| CPV_right | 0.279 | 0.786 | 1.084 | 0.300 | 0.756 | 3.308 | 0.185 | 0.886 | 1.328 | 0.273 | 0.841 | 2.696 | 0.206 | 0.918 | 4.307 |
| CPV_left | 0.079 | 0.825 | 1.084 | 0.082 | 0.756 | 3.308 | 0.048 | 0.886 | 1.328 | 0.078 | 0.841 | 2.696 | 0.051 | 0.949 | 4.307 |
| ALPS_left | -0.229 | 0.786 | 1.084 | -0.215 | 0.756 | 3.308 | -0.036 | 0.886 | 1.328 | -0.219 | 0.841 | 2.696 | 0.018 | 0.952 | 4.307 |
| ALPS_right | 0.051 | 0.837 | 1.084 | 0.134 | 0.756 | 3.308 | 0.246 | 0.886 | 1.328 | 0.081 | 0.841 | 2.696 | 0.405 | 0.918 | 4.307 |
| ALPS | -0.129 | 0.823 | 1.084 | -0.088 | 0.756 | 3.308 | 0.096 | 0.886 | 1.328 | -0.106 | 0.841 | 2.696 | 0.197 | 0.918 | 4.307 |
| negativecoupling | -0.078 | 0.825 | 1.084 | -0.088 | 0.756 | 3.308 | -0.089 | 0.886 | 1.328 | -0.051 | 0.846 | 2.696 | -0.093 | 0.919 | 4.307 |
| Positivecoupling | -0.152 | 0.823 | 1.084 | -0.168 | 0.756 | 3.308 | 0.105 | 0.886 | 1.328 | -0.133 | 0.841 | 2.696 | 0.117 | 0.919 | 4.307 |

Supplementary Table S24. Correlation results between glymphatic dysfunction metrics and apathy severity after regressing out different sets of covariates in the nAPD group.

Five adjustment schemes are shown: Core (age, sex and disease duration), Core + SAS + SDS (age, sex, disease duration, SAS, and SDS), Core + MoCA (age, sex, disease duration, and MoCA), Core + LEDD + UPDRS-III (age, sex, disease duration, LEDD and UPDRS-III) and ALL (age, sex, disease duration, LEDD, UPDRS-III, SAS, SDS, and MoCA). Values are partial correlation coefficients (r) and corresponding p values. VIF max indicates the maximum variance inflation factor among covariates within each scheme. * indicates p‑values before FDR correction.

AES, Apathy Evaluation Scale; SAS, Self-Rating Anxiety Scale; SDS, Self-Rating Depression Scale; MoCA, Montreal Cognitive Assessment; LEDD, Levodopa Equivalent Daily Dose; UPDRS-III, Unified Parkinson’s Disease Rating Scale Part III; CPV, choroid plexus volume; CPV_left, left hemisphere CPV; CPV_right, right hemisphere CPV; PVS, perivascular space volume; PVS_SUB, PVS in subcortical gray matter nuclei; PVS_WM, PVS in the white matter region; pPVS, whole-brain PVS; ALPS, index of diffusion along perivascular spaces; ALPS_left, left hemisphere ALPS; ALPS_right, right hemisphere ALPS.

|  |  | Core | | Core + SAS + SDS | | Core + MoCA | | ALL | |
| --- | --- | --- | --- | --- | --- | --- | --- | --- | --- |
|  |  | *r* | *p* | *r* | *p* | *r* | *p* | *r* | *p* |
| SPC1_All | AES | 0.364 | 0.016 | 0.242 | 0.036* | 0.229 | 0.047* | 0.175 | 0.337 |
|  | PVS_WM | -0.163 | 0.343 | -0.114 | 0.550 | -0.085 | 0.668 | -0.068 | 0.737 |
|  | PVS_SUB | 0.017 | 0.947 | 0.039 | 0.866 | 0.042 | 0.834 | 0.055 | 0.805 |
|  | pPVS | -0.066 | 0.739 | -0.024 | 0.916 | -0.007 | 0.973 | 0.012 | 0.948 |
|  | CPV | 0.451 | 0.001 | 0.425 | 0.006 | 0.396 | 0.014 | 0.398 | 0.020 |
|  | CPV_right | 0.456 | 0.001 | 0.426 | 0.006 | 0.389 | 0.014 | 0.392 | 0.020 |
|  | CPV_left | 0.396 | 0.006 | 0.376 | 0.020 | 0.361 | 0.027 | 0.361 | 0.039 |
|  | ALPS | -0.253 | 0.027* | -0.250 | 0.030* | -0.236 | 0.041* | -0.239 | 0.040* |
|  | ALPS_right | -0.261 | 0.022* | -0.249 | 0.031 | -0.243 | 0.035* | -0.240 | 0.039* |
|  | ALPS_left | -0.200 | 0.199 | -0.209 | 0.207 | -0.187 | 0.273 | -0.197 | 0.263 |
|  | negativecoupling | -0.095 | 0.616 | -0.049 | 0.835 | -0.102 | 0.588 | -0.074 | 0.705 |
|  | positivecoupling | -0.028 | 0.898 | -0.070 | 0.764 | -0.075 | 0.734 | -0.086 | 0.663 |
| SPC2_All | AES | -0.524 | ＜0.001 | -0.352 | 0.032 | -0.376 | 0.019 | -0.272 | 0.019* |
|  | PVS_WM | 0.159 | 0.347 | 0.165 | 0.342 | 0.055 | 0.770 | 0.106 | 0.579 |
|  | PVS_SUB | 0.105 | 0.572 | 0.137 | 0.465 | 0.083 | 0.681 | 0.122 | 0.523 |
|  | pPVS | 0.162 | 0.343 | 0.192 | 0.254 | 0.093 | 0.627 | 0.152 | 0.429 |
|  | CPV | -0.268 | 0.018* | -0.196 | 0.239 | -0.169 | 0.329 | -0.145 | 0.439 |
|  | CPV_right | -0.253 | 0.026* | -0.175 | 0.314 | -0.126 | 0.504 | -0.106 | 0.579 |
|  | CPV_left | -0.257 | 0.024* | -0.199 | 0.229 | -0.199 | 0.227 | -0.173 | 0.344 |
|  | ALPS | 0.312 | 0.035 | 0.326 | 0.041 | 0.301 | 0.008* | 0.317 | 0.006* |
|  | ALPS_right | 0.323 | 0.031 | 0.322 | 0.043 | 0.310 | 0.049 | 0.316 | 0.006* |
|  | ALPS_left | 0.246 | 0.101 | 0.274 | 0.018* | 0.238 | 0.038* | 0.262 | 0.024* |
|  | negativecoupling | 0.083 | 0.668 | -0.011 | 0.968 | 0.093 | 0.627 | 0.020 | 0.920 |
|  | positivecoupling | -0.024 | 0.912 | 0.062 | 0.798 | 0.035 | 0.853 | 0.084 | 0.674 |
| SPC3_All | AES | -0.617 | ＜0.001 | -0.467 | 0.004 | -0.469 | 0.003 | -0.384 | 0.022 |
|  | PVS_WM | 0.229 | 0.045* | 0.200 | 0.229 | 0.119 | 0.535 | 0.132 | 0.495 |
|  | PVS_SUB | -0.047 | 0.738 | -0.089 | 0.680 | -0.116 | 0.534 | -0.118 | 0.539 |
|  | pPVS | 0.059 | 0.783 | 0.025 | 0.916 | -0.037 | 0.851 | -0.034 | 0.856 |
|  | CPV | -0.410 | 0.004 | -0.363 | 0.025 | -0.323 | 0.043 | -0.346 | 0.006* |
|  | CPV_right | -0.430 | 0.002 | -0.381 | 0.019 | -0.321 | 0.043 | -0.319 | 0.006* |
|  | CPV_left | -0.340 | 0.020 | -0.301 | 0.009* | -0.290 | 0.011 | -0.278 | 0.017* |
|  | ALPS | 0.075 | 0.706 | 0.060 | 0.803 | 0.034 | 0.853 | 0.036 | 0.856 |
|  | ALPS_right | 0.088 | 0.644 | 0.056 | 0.822 | 0.045 | 0.821 | 0.035 | 0.856 |
|  | ALPS_left | 0.050 | 0.809 | 0.054 | 0.822 | 0.018 | 0.821 | 0.030 | 0.873 |
|  | negativecoupling | 0.061 | 0.770 | 0.229 | 0.048* | 0.071 | 0.746 | 0.005 | 0.967 |
|  | positivecoupling | 0.127 | 0.482 | -0.032 | 0.890 | 0.220 | 0.175 | 0.266 | 0.022* |
| SPC4_All | AES | -0.307 | 0.036 | -0.245 | 0.034* | -0.200 | 0.227 | -0.195 | 0.263 |
|  | PVS_WM | 0.321 | 0.032 | 0.253 | 0.029* | 0.272 | 0.018* | 0.221 | 0.185 |
|  | PVS_SUB | -0.014 | 0.947 | -0.065 | 0.777 | -0.033 | 0.853 | -0.078 | 0.691 |
|  | pPVS | 0.146 | 0.401 | 0.070 | 0.764 | 0.104 | 0.584 | 0.042 | 0.842 |
|  | CPV | -0.017 | 0.947 | 0.015 | 0.955 | 0.061 | 0.765 | 0.055 | 0.805 |
|  | CPV_right | -0.038 | 0.871 | -0.001 | 0.997 | 0.055 | 0.770 | 0.049 | 0.819 |
|  | CPV_left | 0.009 | 0.960 | 0.033 | 0.889 | 0.060 | 0.765 | 0.055 | 0.805 |
|  | ALPS | 0.357 | 0.017 | 0.365 | 0.025 | 0.346 | 0.035 | 0.358 | 0.039 |
|  | ALPS_right | 0.254 | 0.026* | 0.249 | 0.031* | 0.237 | 0.039* | 0.241 | 0.039* |
|  | ALPS_left | 0.393 | 0.007 | 0.413 | 0.008 | 0.388 | 0.014 | 0.406 | 0.020 |
|  | negativecoupling | 0.103 | 0.579 | 0.090 | 0.680 | 0.108 | 0.564 | 0.111 | 0.563 |
|  | positivecoupling | -0.002 | 0.991 | 0.008 | 0.974 | 0.033 | 0.853 | 0.020 | 0.920 |
| SPC5_All | AES | -0.524 | ＜0.001 | -0.331 | 0.041 | -0.395 | 0.014 | -0.276 | 0.092 |
|  | PVS_WM | 0.205 | 0.189 | 0.161 | 0.361 | 0.115 | 0.546 | 0.117 | 0.539 |
|  | PVS_SUB | -0.081 | 0.675 | -0.112 | 0.560 | -0.119 | 0.535 | -0.129 | 0.499 |
|  | pPVS | 0.036 | 0.871 | -0.013 | 0.966 | -0.041 | 0.834 | -0.051 | 0.817 |
|  | CPV | -0.362 | 0.016 | -0.311 | 0.007* | -0.283 | 0.014* | -0.278 | 0.017* |
|  | CPV_right | -0.346 | 0.019 | -0.286 | 0.013* | -0.245 | 0.033* | -0.243 | 0.151 |
|  | CPV_left | -0.340 | 0.020 | -0.304 | 0.008* | -0.295 | 0.010* | -0.287 | 0.013* |
|  | ALPS | 0.240 | 0.035* | 0.247 | 0.032* | 0.222 | 0.235 | 0.236 | 0.043* |
|  | ALPS_right | 0.230 | 0.044* | 0.217 | 0.185 | 0.208 | 0.209 | 0.207 | 0.226 |
|  | ALPS_left | 0.209 | 0.179 | 0.234 | 0.044* | 0.196 | 0.235 | 0.223 | 0.185 |
|  | negativecoupling | 0.109 | 0.562 | 0.028 | 0.904 | 0.120 | 0.535 | 0.052 | 0.812 |
|  | positivecoupling | 0.015 | 0.947 | 0.096 | 0.649 | 0.073 | 0.738 | 0.112 | 0.559 |

Supplementary Table S25. Correlations between glymphatic dysfunction metrics and microstructural damage metrics as well as apathy after regressing out different sets of covariates, using SPCA results from the whole cohort (PD + HC) based on the microstructural damage metrics that showed significant differences in TBSS analysis between the PD and HC groups.

Four adjustment schemes are shown: Core (age and sex), Core + SAS + SDS (age, sex, SAS, and SDS), Core + MoCA (age, sex, and MoCA), and ALL (age, sex, SAS, SDS, and MoCA). Values are partial correlation coefficients (r) and corresponding p values. * indicates p‑values before FDR correction.

AES, Apathy Evaluation Scale; CPV, choroid plexus volume; CPV_left, left hemisphere CPV; CPV_right, right hemisphere CPV; PVS, perivascular space volume; PVS_SUB, PVS in subcortical gray matter nuclei; PVS_WM, PVS in the white matter region; pPVS, whole-brain PVS; ALPS, index of diffusion along perivascular spaces; ALPS_left, left hemisphere ALPS; ALPS_right, right hemisphere ALPS.

|  | SPC1_All | | SPC2_All | | SPC3_All | | SPC4_All | | SPC5_All | |
| --- | --- | --- | --- | --- | --- | --- | --- | --- | --- | --- |
|  | *r* | *p* | *r* | *p* | *r* | *p* | *r* | *p* | *r* | *p* |
| SAS | 0.310 | 0.025 | -0.364 | 0.006 | -0.448 | ＜0.001 | -0.290 | 0.040 | -0.459 | ＜0.001 |
| SDS | 0.262 | 0.021* | -0.442 | ＜0.001 | -0.445 | ＜0.001 | -0.146 | 0.401 | -0.434 | ＜0.001 |
| MoCA | -0.318 | 0.022 | 0.420 | ＜0.001 | 0.481 | ＜0.001 | 0.254 | 0.026* | 0.389 | 0.003 |

Supplementary Table S26. Correlations between SAS, SDS, MoCA and microstructural damage metrics after regressing out the effects of age and sex, using the SPCA results from the whole cohort (PD + HC) for exploratory analysis of comorbidity mechanisms.

* indicates p‑values before FDR correction.

SAS, Self-Rating Anxiety Scale; SDS, Self-Rating Depression Scale; MoCA, Montreal Cognitive Assessment.

|  |  | Core | | Core + SAS + SDS | | Core + MoCA | | Core + LEDD + UPDRS-III | | ALL | |
| --- | --- | --- | --- | --- | --- | --- | --- | --- | --- | --- | --- |
|  |  | *r* | *p* | *r* | *p* | *r* | *p* | *r* | *p* | *r* | *p* |
| SPC1_PD | AES | 0.263 | 0.189 | 0.174 | 0.472 | 0.182 | 0.390 | 0.264 | 0.207 | 0.143 | 0.569 |
|  | PVS_WM | -0.467 | 0.014 | -0.394 | 0.852 | -0.422 | 0.033 | -0.503 | 0.013 | -0.396 | 0.023* |
|  | PVS_SUB | -0.056 | 0.740 | -0.038 | 0.852 | -0.024 | 0.889 | -0.067 | 0.717 | 0.0441 | 0.855 |
|  | pPVS | -0.218 | 0.252 | -0.106 | 0.645 | -0.167 | 0.414 | -0.237 | 0.244 | -0.093 | 0.703 |
|  | CPV | 0.446 | 0.017 | 0.444 | 0.027 | 0.414 | 0.033 | 0.441 | 0.021 | 0.434 | 0.012* |
|  | CPV_right | 0.473 | 0.014 | 0.484 | 0.022 | 0.433 | 0.031 | 0.464 | 0.020 | 0.466 | 0.006* |
|  | CPV_left | 0.369 | 0.023* | 0.351 | 0.036* | 0.355 | 0.031* | 0.372 | 0.026* | 0.362 | 0.038* |
|  | ALPS | -0.449 | 0.017 | -0.451 | 0.026 | -0.440 | 0.031 | -0.437 | 0.021 | -0.428 | 0.013* |
|  | ALPS_right | -0.346 | 0.033* | -0.336 | 0.045* | -0.334 | 0.043* | -0.335 | 0.046* | -0.301 | 0.187 |
|  | ALPS_left | -0.456 | 0.016 | -0.466 | 0.023 | -0.450 | 0.031 | -0.446 | 0.021 | -0.458 | 0.007* |
|  | negativecoupling | -0.240 | 0.228 | -0.262 | 0.230 | -0.283 | 0.170 | -0.225 | 0.258 | -0.316 | 0.165 |
|  | positivecoupling | 0.121 | 0.544 | 0.124 | 0.583 | 0.085 | 0.679 | 0.101 | 0.630 | 0.095 | 0.703 |
| SPC2_PD | AES | 0.251 | 0.210 | 0.172 | 0.472 | 0.197 | 0.364 | 0.259 | 0.207 | 0.173 | 0.527 |
|  | PVS_WM | -0.365 | 0.024* | -0.326 | 0.111 | -0.330 | 0.046* | -0.378 | 0.022* | -0.318 | 0.165 |
|  | PVS_SUB | -0.106 | 0.592 | -0.047 | 0.829 | -0.084 | 0.679 | -0.117 | 0.579 | -0.048 | 0.855 |
|  | pPVS | -0.223 | 0.248 | -0.157 | 0.481 | -0.187 | 0.387 | -0.234 | 0.244 | -0.150 | 0.567 |
|  | CPV | 0.680 | ＜0.001 | 0.678 | ＜0.001 | 0.668 | ＜0.001 | 0.677 | ＜0.001 | 0.677 | ＜0.001 |
|  | CPV_right | 0.674 | ＜0.001 | 0.680 | ＜0.001 | 0.665 | ＜0.001 | 0.671 | ＜0.001 | 0.682 | ＜0.001 |
|  | CPV_left | 0.618 | ＜0.001 | 0.608 | 0.001 | 0.612 | ＜0.001 | 0.623 | ＜0.001 | 0.615 | 0.002 |
|  | ALPS | -0.224 | 0.248 | -0.209 | 0.361 | -0.211 | 0.343 | -0.211 | 0.289 | -0.195 | 0.453 |
|  | ALPS_right | -0.237 | 0.228 | -0.218 | 0.345 | -0.225 | 0.311 | -0.236 | 0.244 | -0.211 | 0.410 |
|  | ALPS_left | -0.174 | 0.382 | -0.165 | 0.481 | -0.163 | 0.417 | -0.154 | 0.461 | -0.149 | 0.567 |
|  | negativecoupling | -0.146 | 0.457 | -0.142 | 0.526 | -0.175 | 0.399 | -0.124 | 0.567 | -0.161 | 0.557 |
|  | positivecoupling | 0.100 | 0.595 | 0.085 | 0.725 | 0.073 | 0.708 | 0.081 | 0.678 | 0.063 | 0.820 |
| SPC3_PD | AES | -0.493 | 0.011 | -0.370 | 0.027* | -0.417 | 0.033 | -0.489 | 0.013 | -0.295 | 0.187 |
|  | PVS_WM | 0.388 | 0.044 | 0.320 | 0.115 | 0.305 | 0.133 | 0.441 | 0.021 | 0.293 | 0.187 |
|  | PVS_SUB | 0.148 | 0.457 | 0.054 | 0.829 | 0.111 | 0.596 | 0.159 | 0.455 | 0.025 | 0.889 |
|  | pPVS | 0.266 | 0.189 | 0.161 | 0.481 | 0.201 | 0.363 | 0.292 | 0.159 | 0.121 | 0.646 |
|  | CPV | -0.490 | 0.011 | -0.464 | 0.023 | -0.451 | 0.031 | -0.490 | 0.013 | -0.448 | 0.009* |
|  | CPV_right | -0.490 | 0.011 | -0.468 | 0.023 | -0.435 | 0.031 | -0.489 | 0.013 | -0.443 | 0.010* |
|  | CPV_left | -0.441 | 0.017 | -0.413 | 0.045 | -0.431 | 0.031 | -0.446 | 0.021 | -0.416 | 0.016* |
|  | ALPS | 0.360 | 0.026* | 0.350 | 0.088 | 0.348 | 0.035* | 0.346 | 0.039* | 0.348 | 0.047* |
|  | ALPS_right | 0.285 | 0.157 | 0.259 | 0.230 | 0.269 | 0.193 | 0.260 | 0.207 | 0.227 | 0.366 |
|  | ALPS_left | 0.359 | 0.027* | 0.364 | 0.029* | 0.352 | 0.032* | 0.358 | 0.032* | 0.387 | 0.026* |
|  | negativecoupling | 0.061 | 0.739 | 0.028 | 0.870 | 0.111 | 0.596 | 0.055 | 0.748 | 0.113 | 0.661 |
|  | positivecoupling | -0.097 | 0.595 | -0.051 | 0.829 | -0.048 | 0.802 | -0.083 | 0.678 | -0.028 | 0.889 |

Supplementary Table S27. Correlations between glymphatic dysfunction metrics and microstructural damage metrics as well as apathy after regressing out different sets of covariates, using SPCA results from the PD cohort based on the microstructural damage metrics that showed significant differences in TBSS analysis between the PD and HC groups.

Five adjustment schemes are shown: Core (age, sex and disease duration), Core + SAS + SDS (age, sex, disease duration, SAS, and SDS), Core + MoCA (age, sex, disease duration, and MoCA), Core + LEDD + UPDRS-III (age, sex, disease duration, LEDD and UPDRS-III) and ALL (age, sex, disease duration, LEDD, UPDRS-III, SAS, SDS, and MoCA). Values are partial correlation coefficients (r) and corresponding p values. * indicates p‑values before FDR correction.

AES, Apathy Evaluation Scale; SAS, Self-Rating Anxiety Scale; SDS, Self-Rating Depression Scale; MoCA, Montreal Cognitive Assessment; LEDD, Levodopa Equivalent Daily Dose; UPDRS-III, Unified Parkinson’s Disease Rating Scale Part III; CPV, choroid plexus volume; CPV_left, left hemisphere CPV; CPV_right, right hemisphere CPV; PVS, perivascular space volume; PVS_SUB, PVS in subcortical gray matter nuclei; PVS_WM, PVS in the white matter region; pPVS, whole-brain PVS; ALPS, index of diffusion along perivascular spaces; ALPS_left, left hemisphere ALPS; ALPS_right, right hemisphere ALPS.

|  | SPC1_PD | | SPC2_PD | | SPC3_PD | |
| --- | --- | --- | --- | --- | --- | --- |
|  | *r* | *p* | *r* | *p* | *r* | *p* |
| SAS | 0.272 | 0.222 | 0.220 | 0.276 | -0.399 | 0.013* |
| SDS | 0.108 | 0.517 | 0.135 | 0.472 | -0.293 | 0.222 |
| MoCA | -0.225 | 0.276 | -0.168 | 0.402 | 0.301 | 0.222 |

Supplementary Table S28. Correlations between SAS, SDS, MoCA and microstructural damage metrics after regressing out the effects of age, sex and disease duration, using the SPCA results from the PD cohort for exploratory analysis of comorbidity mechanisms.

* indicates p‑values before FDR correction.

SAS, Self-Rating Anxiety Scale; SDS, Self-Rating Depression Scale; MoCA, Montreal Cognitive Assessment.

|  |  | Core | | Core + SAS + SDS | | Core + MoCA | | Core + LEDD + UPDRS-III | | ALL | |
| --- | --- | --- | --- | --- | --- | --- | --- | --- | --- | --- | --- |
|  |  | *r* | *p* | *r* | *p* | *r* | *p* | *r* | *p* | *r* | *p* |
| FWcluster1 | AES | 0.359 | 0.027 | 0.289 | 0.270 | 0.348 | 0.035* | 0.361 | 0.031* | 0.334 | 0.258 |
|  | PVS_WM | -0.110 | 0.710 | -0.015 | 0.930 | -0.069 | 0.822 | -0.115 | 0.672 | -0.004 | 0.999 |
|  | PVS_SUB | 0.061 | 0.836 | 0.141 | 0.622 | 0.079 | 0.797 | 0.051 | 0.862 | 0.138 | 0.628 |
|  | pPVS | 0.011 | 0.976 | 0.118 | 0.657 | 0.044 | 0.871 | 0.002 | 0.989 | 0.122 | 0.662 |
|  | CPV | 0.412 | 0.010* | 0.393 | 0.018* | 0.398 | 0.015* | 0.405 | 0.014* | 0.395 | 0.023* |
|  | CPV_right | 0.290 | 0.214 | 0.268 | 0.295 | 0.269 | 0.352 | 0.278 | 0.281 | 0.266 | 0.406 |
|  | CPV_left | 0.518 | 0.020 | 0.502 | 0.002* | 0.512 | 0.043 | 0.520 | 0.027 | 0.510 | 0.002* |
|  | ALPS | -0.102 | 0.725 | -0.081 | 0.768 | -0.091 | 0.760 | -0.085 | 0.800 | -0.066 | 0.856 |
|  | ALPS_right | -0.150 | 0.631 | -0.126 | 0.643 | -0.141 | 0.661 | -0.145 | 0.590 | -0.118 | 0.662 |
|  | ALPS_left | -0.043 | 0.870 | -0.029 | 0.918 | -0.034 | 0.891 | -0.021 | 0.987 | -0.012 | 0.999 |
|  | negativecoupling | 0.118 | 0.691 | 0.235 | 0.376 | 0.129 | 0.661 | 0.162 | 0.567 | 0.255 | 0.423 |
|  | positivecoupling | -0.092 | 0.750 | -0.137 | 0.622 | -0.129 | 0.661 | -0.137 | 0.590 | -0.168 | 0.558 |
| FWcluster2 | AES | 0.493 | 0.020 | 0.380 | 0.022* | 0.377 | 0.022* | 0.493 | 0.027 | 0.228 | 0.438 |
|  | PVS_WM | -0.138 | 0.671 | -0.158 | 0.587 | 0.027 | 0.897 | -0.140 | 0.590 | -0.021 | 0.992 |
|  | PVS_SUB | 0.057 | 0.836 | 0.111 | 0.663 | 0.126 | 0.661 | 0.054 | 0.862 | 0.183 | 0.558 |
|  | pPVS | -0.003 | 0.986 | 0.043 | 0.878 | 0.117 | 0.678 | -0.005 | 0.989 | 0.156 | 0.579 |
|  | CPV | 0.356 | 0.028* | 0.295 | 0.270 | 0.293 | 0.283 | 0.353 | 0.035* | 0.247 | 0.428 |
|  | CPV_right | 0.354 | 0.029* | 0.281 | 0.270 | 0.258 | 0.358 | 0.352 | 0.035* | 0.209 | 0.486 |
|  | CPV_left | 0.323 | 0.048* | 0.283 | 0.270 | 0.306 | 0.261 | 0.322 | 0.166 | 0.269 | 0.406 |
|  | ALPS | -0.207 | 0.480 | -0.181 | 0.525 | -0.184 | 0.551 | -0.210 | 0.563 | -0.226 | 0.438 |
|  | ALPS_right | -0.171 | 0.575 | -0.135 | 0.622 | -0.184 | 0.551 | -0.178 | 0.567 | -0.166 | 0.558 |
|  | ALPS_left | -0.201 | 0.480 | -0.187 | 0.523 | -0.145 | 0.661 | -0.199 | 0.567 | -0.234 | 0.438 |
|  | Negativecoupling | -0.080 | 0.788 | 0.046 | 0.878 | -0.052 | 0.881 | -0.069 | 0.827 | -0.003 | 0.999 |
|  | positivecoupling | -0.118 | 0.691 | -0.247 | 0.353 | -0.254 | 0.358 | -0.143 | 0.590 | -0.348 | 0.047* |
| FWcluster3 | AES | 0.501 | 0.020 | 0.390 | 0.019* | 0.446 | 0.006* | 0.506 | 0.027 | 0.370 | 0.034* |
|  | PVS_WM | -0.314 | 0.165 | -0.297 | 0.270 | -0.237 | 0.405 | -0.355 | 0.034* | -0.273 | 0.406 |
|  | PVS_SUB | -0.055 | 0.836 | 0.016 | 0.930 | -0.018 | 0.914 | -0.071 | 0.827 | 0.034 | 0.958 |
|  | pPVS | -0.161 | 0.601 | -0.091 | 0.742 | -0.098 | 0.750 | -0.185 | 0.567 | -0.061 | 0.856 |
|  | CPV | 0.453 | 0.036 | 0.410 | 0.013* | 0.417 | 0.010* | 0.448 | 0.006* | 0.393 | 0.024* |
|  | CPV_right | 0.447 | 0.036 | 0.401 | 0.015* | 0.397 | 0.015* | 0.435 | 0.008* | 0.372 | 0.033* |
|  | CPV_left | 0.415 | 0.010* | 0.381 | 0.022* | 0.402 | 0.014* | 0.423 | 0.010* | 0.384 | 0.027* |
|  | ALPS | -0.219 | 0.480 | -0.193 | 0.523 | -0.201 | 0.551 | -0.189 | 0.567 | -0.176 | 0.558 |
|  | ALPS_right | -0.193 | 0.491 | -0.158 | 0.587 | -0.174 | 0.573 | -0.167 | 0.567 | -0.135 | 0.628 |
|  | ALPS_left | -0.202 | 0.480 | -0.188 | 0.523 | -0.188 | 0.551 | -0.173 | 0.567 | -0.178 | 0.558 |
|  | negativecoupling | 0.125 | 0.691 | 0.293 | 0.270 | 0.152 | 0.661 | 0.165 | 0.567 | 0.288 | 0.406 |
|  | positivecoupling | 0.037 | 0.874 | -0.048 | 0.878 | -0.035 | 0.891 | 0.006 | 0.989 | -0.081 | 0.811 |

Supplementary Table S29. Correlations between glymphatic dysfunction metrics and microstructural damage metrics as well as apathy after regressing out different sets of covariates, using SPCA results from the PD cohort based on the microstructural damage metrics that showed significant differences in TBSS analysis between the APD and nAPD groups.

Five adjustment schemes are shown: Core (age, sex and disease duration), Core + SAS + SDS (age, sex, disease duration, SAS, and SDS), Core + MoCA (age, sex, disease duration, and MoCA), Core + LEDD + UPDRS-III (age, sex, disease duration, LEDD and UPDRS-III) and ALL (age, sex, disease duration, LEDD, UPDRS-III, SAS, SDS, and MoCA). Values are partial correlation coefficients (r) and corresponding p values. * indicates p‑values before FDR correction.

AES, Apathy Evaluation Scale; SAS, Self-Rating Anxiety Scale; SDS, Self-Rating Depression Scale; MoCA, Montreal Cognitive Assessment; LEDD, Levodopa Equivalent Daily Dose; UPDRS-III, Unified Parkinson’s Disease Rating Scale Part III; CPV, choroid plexus volume; CPV_left, left hemisphere CPV; CPV_right, right hemisphere CPV; PVS, perivascular space volume; PVS_SUB, PVS in subcortical gray matter nuclei; PVS_WM, PVS in the white matter region; pPVS, whole-brain PVS; ALPS, index of diffusion along perivascular spaces; ALPS_left, left hemisphere ALPS; ALPS_right, right hemisphere ALPS.

|  | FWcluster1 | | FWcluster2 | | FWcluster3 | |
| --- | --- | --- | --- | --- | --- | --- |
|  | *r* | *p* | *r* | *p* | *r* | *p* |
| SAS | 0.248 | 0.172 | 0.318 | 0.092 | 0.344 | 0.035 |
| SDS | 0.154 | 0.399 | 0.407 | 0.011* | 0.330 | 0.043* |
| MoCA | -0.115 | 0.492 | -394 | 0.014* | -0.257 | 0.172 |

Supplementary Table S30. Correlations between SAS, SDS, MoCA and microstructural damage metrics after regressing out the effects of age, sex and disease duration, using the significant differences in TBSS analysis between the APD and nAPD groups from PD cohort for exploratory analysis of comorbidity mechanisms.

* indicates p‑values before FDR correction.

SAS, Self-Rating Anxiety Scale; SDS, Self-Rating Depression Scale; MoCA, Montreal Cognitive Assessment.

|  |  | Core | | Core + SAS + SDS | | Core + MoCA | | Core + LEDD + UPDRS-III | | ALL | |
| --- | --- | --- | --- | --- | --- | --- | --- | --- | --- | --- | --- |
|  |  | *r* | *p* | *r* | *p* | *r* | *p* | *r* | *p* | *r* | *p* |
| FWcluster1 | AES | -0.171 | 0.886 | 0.071 | 0.995 | -0.155 | 0.911 | -0.151 | 0.943 | 0.130 | 0.809 |
|  | PVS_WM | -0.186 | 0.886 | -0.324 | 0.611 | -0.246 | 0.892 | -0.139 | 0.943 | -0.414 | 0.692 |
|  | PVS_SUB | 0.090 | 0.892 | 0.184 | 0.954 | 0.080 | 0.931 | 0.043 | 0.951 | 0.147 | 0.808 |
|  | pPVS | 0.026 | 0.979 | 0.110 | 0.995 | 0.006 | 0.983 | -0.001 | 0.999 | 0.049 | 0.950 |
|  | CPV | 0.265 | 0.824 | 0.438 | 0.590 | 0.300 | 0.711 | 0.256 | 0.762 | 0.452 | 0.692 |
|  | CPV_right | 0.109 | 0.886 | 0.335 | 0.611 | 0.157 | 0.911 | 0.107 | 0.943 | 0.350 | 0.692 |
|  | CPV_left | 0.457 | 0.386 | 0.511 | 0.590 | 0.460 | 0.434 | 0.445 | 0.572 | 0.541 | 0.692 |
|  | ALPS | -0.232 | 0.886 | -0.316 | 0.611 | -0.229 | 0.892 | -0.283 | 0.762 | -0.296 | 0.692 |
|  | ALPS_right | -0.336 | 0.656 | -0.353 | 0.611 | -0.337 | 0.711 | -0.392 | 0.649 | -0.358 | 0.692 |
|  | ALPS_left | -0.068 | 0.932 | -0.229 | 0.815 | -0.059 | 0.968 | -0.090 | 0.943 | -0.175 | 0.781 |
|  | negativecoupling | 0.325 | 0.656 | 0.336 | 0.611 | 0.323 | 0.711 | 0.342 | 0.760 | 0.326 | 0.692 |
|  | positivecoupling | -0.136 | 0.886 | -0.034 | 0.995 | -0.102 | 0.922 | -0.191 | 0.925 | 0.044 | 0.950 |
| FWcluster2 | AES | 0.219 | 0.886 | -0.031 | 0.995 | 0.216 | 0.892 | 0.288 | 0.762 | 0.012 | 0.971 |
|  | PVS_WM | 0.007 | 0.979 | -0.078 | 0.995 | 0.025 | 0.983 | 0.144 | 0.943 | 0.123 | 0.809 |
|  | PVS_SUB | 0.146 | 0.886 | 0.047 | 0.995 | 0.152 | 0.911 | 0.065 | 0.951 | -0.291 | 0.692 |
|  | pPVS | 0.134 | 0.886 | 0.029 | 0.995 | 0.146 | 0.911 | 0.096 | 0.943 | -0.255 | 0.729 |
|  | CPV | 0.458 | 0.386 | 0.410 | 0.590 | 0.465 | 0.434 | 0.457 | 0.572 | 0.445 | 0.692 |
|  | CPV_right | 0.470 | 0.386 | 0.381 | 0.590 | 0.493 | 0.434 | 0.506 | 0.528 | 0.501 | 0.692 |
|  | CPV_left | 0.351 | 0.656 | 0.386 | 0.590 | 0.351 | 0.711 | 0.316 | 0.762 | 0.327 | 0.692 |
|  | ALPS | -0.031 | 0.979 | 0.020 | 0.995 | -0.033 | 0.983 | -0.093 | 0.943 | -0.235 | 0.731 |
|  | ALPS_right | 0.089 | 0.892 | 0.076 | 0.995 | 0.089 | 0.931 | 0.035 | 0.951 | -0.187 | 0.781 |
|  | ALPS_left | -0.180 | 0.886 | -0.060 | 0.995 | -0.185 | 0.911 | -0.250 | 0.762 | -0.281 | 0.692 |
|  | Negativecoupling | -0.047 | 0.886 | -0.040 | 0.995 | -0.045 | 0.981 | -0.028 | 0.951 | -0.019 | 0.971 |
|  | positivecoupling | 0.121 | 0.886 | 0.017 | 0.995 | 0.128 | 0.911 | 0.043 | 0.951 | -0.285 | 0.692 |
| FWcluster3 | AES | 0.216 | 0.886 | 0.056 | 0.995 | 0.213 | 0.892 | 0.244 | 0.762 | 0.180 | 0.781 |
|  | PVS_WM | -0.383 | 0.573 | -0.383 | 0.590 | -0.403 | 0.544 | -0.380 | 0.649 | -0.504 | 0.692 |
|  | PVS_SUB | -0.014 | 0.979 | 0.007 | 0.995 | -0.010 | 0.983 | -0.161 | 0.943 | -0.213 | 0.763 |
|  | pPVS | -0.125 | 0.886 | -0.079 | 0.995 | -0.120 | 0.911 | -0.243 | 0.762 | -0.322 | 0.692 |
|  | CPV | 0.599 | 0.014* | 0.554 | 0.040* | 0.610 | 0.016* | 0.585 | 0.028* | 0.601 | 0.692 |
|  | CPV_right | 0.603 | 0.013* | 0.563 | 0.040* | 0.640 | 0.010* | 0.576 | 0.031* | 0.635 | 0.036* |
|  | CPV_left | 0.473 | 0.386 | 0.458 | 0.590 | 0.473 | 0.434 | 0.513 | 0.528 | 0.496 | 0.692 |
|  | ALPS | -0.422 | 0.465 | -0.380 | 0.590 | -0.425 | 0.517 | -0.410 | 0.649 | -0.379 | 0.692 |
|  | ALPS_right | -0.300 | 0.716 | -0.293 | 0.654 | -0.301 | 0.711 | -0.300 | 0.762 | -0.318 | 0.692 |
|  | ALPS_left | -0.518 | 0.040* | -0.460 | 0.590 | -0.525 | 0.044* | -0.493 | 0.528 | -0.429 | 0.692 |
|  | negativecoupling | 0.126 | 0.886 | 0.253 | 0.764 | 0.128 | 0.911 | 0.129 | 0.943 | 0.246 | 0.729 |
|  | positivecoupling | 0.110 | 0.886 | 0.002 | 0.995 | 0.115 | 0.911 | 0.065 | 0.951 | 0.144 | 0.808 |

Supplementary Table S31. Correlations between glymphatic dysfunction metrics and microstructural damage metrics as well as apathy after regressing out different sets of covariates, using SPCA results from the APD cohort based on the microstructural damage metrics that showed significant differences in TBSS analysis between the APD and nAPD groups.

Five adjustment schemes are shown: Core (age, sex and disease duration), Core + SAS + SDS (age, sex, disease duration, SAS, and SDS), Core + MoCA (age, sex, disease duration, and MoCA), Core + LEDD + UPDRS-III (age, sex, disease duration, LEDD and UPDRS-III) and ALL (age, sex, disease duration, LEDD, UPDRS-III, SAS, SDS, and MoCA). Values are partial correlation coefficients (r) and corresponding p values. * indicates p‑values before FDR correction.

AES, Apathy Evaluation Scale; SAS, Self-Rating Anxiety Scale; SDS, Self-Rating Depression Scale; MoCA, Montreal Cognitive Assessment; LEDD, Levodopa Equivalent Daily Dose; UPDRS-III, Unified Parkinson’s Disease Rating Scale Part III; CPV, choroid plexus volume; CPV_left, left hemisphere CPV; CPV_right, right hemisphere CPV; PVS, perivascular space volume; PVS_SUB, PVS in subcortical gray matter nuclei; PVS_WM, PVS in the white matter region; pPVS, whole-brain PVS; ALPS, index of diffusion along perivascular spaces; ALPS_left, left hemisphere ALPS; ALPS_right, right hemisphere ALPS.

|  | FWcluster1 | | FWcluster2 | | FWcluster3 | |
| --- | --- | --- | --- | --- | --- | --- |
|  | *r* | *p* | *r* | *p* | *r* | *p* |
| SAS | -0.117 | 0.893 | 0.062 | 0.893 | 0.249 | 0.794 |
| SDS | -0.328 | 0.794 | 0.358 | 0.794 | 0.264 | 0.794 |
| MoCA | 0.091 | 0.893 | -0.039 | 0.893 | -0.037 | 0.893 |

Supplementary Table S32. Correlations between SAS, SDS, MoCA and microstructural damage metrics after regressing out the effects of age, sex and disease duration, using the significant differences in TBSS analysis between the APD and nAPD groups from PD cohort for exploratory analysis of comorbidity mechanisms.

* indicates p‑values before FDR correction.

SAS, Self-Rating Anxiety Scale; SDS, Self-Rating Depression Scale; MoCA, Montreal Cognitive Assessment.

|  |  | Core | | Core + SAS + SDS | | Core + MoCA | | Core + LEDD + UPDRS-III | | ALL | |
| --- | --- | --- | --- | --- | --- | --- | --- | --- | --- | --- | --- |
|  |  | *r* | *p* | *r* | *p* | *r* | *p* | *r* | *p* | *r* | *p* |
| FWcluster1 | AES | 0.172 | 0.783 | 0.125 | 0.803 | 0.213 | 0.857 | 0.143 | 0.788 | 0.124 | 0.902 |
|  | PVS_WM | 0.181 | 0.783 | 0.209 | 0.803 | 0.178 | 0.857 | 0.151 | 0.788 | 0.260 | 0.753 |
|  | PVS_SUB | 0.084 | 0.811 | 0.110 | 0.803 | 0.082 | 0.865 | 0.111 | 0.788 | 0.123 | 0.902 |
|  | pPVS | 0.135 | 0.794 | 0.160 | 0.803 | 0.132 | 0.857 | 0.141 | 0.788 | 0.190 | 0.843 |
|  | CPV | 0.315 | 0.734 | 0.347 | 0.759 | 0.332 | 0.857 | 0.323 | 0.619 | 0.361 | 0.753 |
|  | CPV_right | 0.203 | 0.783 | 0.242 | 0.803 | 0.227 | 0.857 | 0.199 | 0.788 | 0.246 | 0.753 |
|  | CPV_left | 0.417 | 0.570 | 0.438 | 0.617 | 0.424 | 0.857 | 0.437 | 0.574 | 0.462 | 0.753 |
|  | ALPS | -0.149 | 0.783 | -0.102 | 0.803 | -0.198 | 0.857 | -0.101 | 0.788 | -0.078 | 0.905 |
|  | ALPS_right | -0.101 | 0.811 | -0.023 | 0.956 | -0.130 | 0.857 | -0.035 | 0.921 | -0.048 | 0.905 |
|  | ALPS_left | -0.156 | 0.783 | -0.136 | 0.803 | -0.206 | 0.857 | -0.130 | 0.788 | -0.086 | 0.905 |
|  | negativecoupling | -0.039 | 0.898 | 0.014 | 0.957 | -0.036 | 0.914 | 0.154 | 0.788 | 0.232 | 0.765 |
|  | positivecoupling | -0.175 | 0.783 | -0.139 | 0.803 | -0.206 | 0.857 | -0.379 | 0.611 | -0.415 | 0.753 |
| FWcluster2 | AES | 0.103 | 0.811 | 0.078 | 0.835 | -0.237 | 0.857 | 0.104 | 0.788 | -0.309 | 0.753 |
|  | PVS_WM | -0.095 | 0.811 | -0.143 | 0.803 | -0.065 | 0.865 | -0.113 | 0.788 | -0.150 | 0.898 |
|  | PVS_SUB | 0.073 | 0.811 | 0.117 | 0.803 | 0.128 | 0.857 | 0.004 | 0.988 | 0.035 | 0.905 |
|  | pPVS | 0.016 | 0.949 | 0.029 | 0.956 | 0.069 | 0.865 | -0.044 | 0.919 | -0.036 | 0.905 |
|  | CPV | 0.169 | 0.783 | 0.165 | 0.803 | 0.059 | 0.865 | 0.204 | 0.788 | 0.105 | 0.905 |
|  | CPV_right | 0.191 | 0.783 | 0.185 | 0.803 | 0.016 | 0.949 | 0.236 | 0.781 | 0.062 | 0.905 |
|  | CPV_left | 0.130 | 0.794 | 0.129 | 0.803 | 0.101 | 0.857 | 0.153 | 0.788 | 0.144 | 0.898 |
|  | ALPS | -0.543 | 0.016* | -0.533 | 0.028* | -0.351 | 0.857 | -0.557 | 0.020* | -0.363 | 0.753 |
|  | ALPS_right | -0.579 | 0.009* | -0.569 | 0.017* | -0.479 | 0.044* | -0.552 | 0.022* | -0.396 | 0.753 |
|  | ALPS_left | -0.430 | 0.570 | -0.429 | 0.617 | -0.187 | 0.857 | -0.480 | 0.462 | -0.281 | 0.753 |
|  | Negativecoupling | -0.156 | 0.783 | -0.096 | 0.803 | -0.263 | 0.857 | -0.178 | 0.788 | -0.360 | 0.753 |
|  | positivecoupling | -0.413 | 0.570 | -0.484 | 0.049* | -0.262 | 0.857 | -0.569 | 0.017* | -0.540 | 0.046* |
| FWcluster3 | AES | 0.253 | 0.783 | 0.276 | 0.768 | 0.106 | 0.857 | 0.286 | 0.700 | 0.195 | 0.843 |
|  | PVS_WM | -0.225 | 0.783 | -0.268 | 0.768 | -0.216 | 0.857 | -0.223 | 0.781 | -0.322 | 0.753 |
|  | PVS_SUB | -0.110 | 0.811 | -0.103 | 0.803 | -0.102 | 0.857 | -0.223 | 0.781 | -0.271 | 0.753 |
|  | pPVS | -0.172 | 0.783 | -0.178 | 0.803 | -0.163 | 0.857 | -0.251 | 0.781 | -0.318 | 0.753 |
|  | CPV | 0.319 | 0.734 | 0.310 | 0.759 | 0.269 | 0.857 | 0.372 | 0.611 | 0.337 | 0.753 |
|  | CPV_right | 0.312 | 0.734 | 0.297 | 0.759 | 0.229 | 0.857 | 0.380 | 0.611 | 0.300 | 0.753 |
|  | CPV_left | 0.302 | 0.734 | 0.301 | 0.759 | 0.294 | 0.857 | 0.334 | 0.619 | 0.355 | 0.753 |
|  | ALPS | -0.316 | 0.734 | -0.339 | 0.759 | -0.159 | 0.857 | -0.353 | 0.611 | -0.253 | 0.753 |
|  | ALPS_right | -0.292 | 0.734 | -0.330 | 0.759 | -0.172 | 0.857 | -0.282 | 0.700 | -0.149 | 0.898 |
|  | ALPS_left | -0.281 | 0.734 | -0.294 | 0.759 | -0.117 | 0.857 | -0.349 | 0.611 | -0.283 | 0.753 |
|  | negativecoupling | 0.188 | 0.783 | 0.217 | 0.803 | 0.174 | 0.857 | 0.130 | 0.788 | 0.056 | 0.905 |
|  | positivecoupling | -0.078 | 0.811 | -0.134 | 0.803 | 0.068 | 0.865 | -0.065 | 0.879 | 0.040 | 0.905 |

Supplementary Table S33. Correlations between glymphatic dysfunction metrics and microstructural damage metrics as well as apathy after regressing out different sets of covariates, using SPCA results from the nAPD cohort based on the microstructural damage metrics that showed significant differences in TBSS analysis between the APD and nAPD groups.

Five adjustment schemes are shown: Core (age, sex and disease duration), Core + SAS + SDS (age, sex, disease duration, SAS, and SDS), Core + MoCA (age, sex, disease duration, and MoCA), Core + LEDD + UPDRS-III (age, sex, disease duration, LEDD and UPDRS-III) and ALL (age, sex, disease duration, LEDD, UPDRS-III, SAS, SDS, and MoCA). Values are partial correlation coefficients (r) and corresponding p values. * indicates p‑values before FDR correction.

AES, Apathy Evaluation Scale; SAS, Self-Rating Anxiety Scale; SDS, Self-Rating Depression Scale; MoCA, Montreal Cognitive Assessment; LEDD, Levodopa Equivalent Daily Dose; UPDRS-III, Unified Parkinson’s Disease Rating Scale Part III; CPV, choroid plexus volume; CPV_left, left hemisphere CPV; CPV_right, right hemisphere CPV; PVS, perivascular space volume; PVS_SUB, PVS in subcortical gray matter nuclei; PVS_WM, PVS in the white matter region; pPVS, whole-brain PVS; ALPS, index of diffusion along perivascular spaces; ALPS_left, left hemisphere ALPS; ALPS_right, right hemisphere ALPS.

|  | FWcluster1 | | FWcluster2 | | FWcluster3 | |
| --- | --- | --- | --- | --- | --- | --- |
|  | *r* | *p* | *r* | *p* | *r* | *p* |
| SAS | 0.234 | 0.887 | 0.150 | 0.887 | -0.048 | 0.887 |
| SDS | 0.108 | 0.887 | 0.174 | 0.887 | 0.035 | 0.887 |
| MoCA | 0.052 | 0.887 | -0.637 | 0.030 | -0.390 | 0.445 |

Supplementary Table S34. Correlations between SAS, SDS, MoCA and microstructural damage metrics after regressing out the effects of age, sex and disease duration, using the significant differences in TBSS analysis between the APD and nAPD groups from nAPD cohort for exploratory analysis of comorbidity mechanisms.

* indicates p‑values before FDR correction.

SAS, Self-Rating Anxiety Scale; SDS, Self-Rating Depression Scale; MoCA, Montreal Cognitive Assessment.

| X | M | a path (SE) | a path *p* | b path (SE) | b path *p* | c'(ADE) (SE) | c' *p* | ab (ACME) [95% CI] | ab *pFDR* | c(Total) (SE) | c *p* | Type | % |
| --- | --- | --- | --- | --- | --- | --- | --- | --- | --- | --- | --- | --- | --- |
| SPC2_All | ALPS_right | 0.038 (0.013) | 0.004 | 10.749 (4.000) | 0.009 | -2.861 (0.467) | <0.001 | 0.41  [0.07, 0.90] | 0.043* | -2.46  (0.460) | <0.001 | SC | — |
| SPC2_All | ALPS | 0.034 (0.012) | 0.006 | 10.724 (4.293) | 0.015 | -2.820 (0.468) | <0.001 | 0.36  [0.05, 0.86] | 0.047* | -2.46  (0.460) | <0.001 | SC | — |
| CPV | SCP3_All | -1634.31 (419.99) | <0.001 | -2.769 (0.470) | <0.001 | 1250.58 (1872.40) | 0.506 | 4525.60  [2182.88, 7452.52] | 0.006* | 5776.18  (2056.85) | 0.006 | CM | 78.3 |
| CPV_right | SPC3_All | -3001.58 (727.12) | <0.001 | -2.755 (0.474) | <0.001 | 2310.32 (3309.00) | 0.487 | 8269.81  [4251.64, 12971.58] | 0.006* | 10580.13  (3580.02) | 0.004 | CM | 78.2 |
| CPV_left | SPC3_All | -2818.70 (899.12) | 0.002 | -2.812 (0.456) | <0.001 | 2075.01 (3775.80) | 0.584 | 7927.24  [3061.92, 13991.32] | 0.008* | 10002.25  (4339.46) | 0.024 | CM | 79.3 |
| CPV | SPC5_All | -1674.20 (498.07) | 0.001 | -1.921 (0.425) | <0.001 | 2560.86 (1966.38) | 0.197 | 3215.31  [1122.16, 5898.10] | 0.012* | 5776.18  (2056.85) | 0.006 | CM | 55.7 |
| CPV_left | SPC5_All | -3271.68 (1043.26) | 0.002 | -1.998 (0.424) | <0.001 | 3465.50 (4075.18) | 0.398 | 6536.75  [1977.17, 12691.22] | 0.012* | 10002.25  (4339.46) | 0.024 | CM | 65.4 |
| CPV_rigth | SPC5_All | -2802.22 (876.87) | 0.002 | -1.895 (0.420) | <0.001 | 5268.69 (3402.58) | 0.126 | 5311.44  [1822.98, 9643.49] | 0.012* | 10580.13  (3580.02) | 0.004 | CM | 50.2 |
| CPV_left | SPC1_All | 3574.80 (956.28) | <0.001 | 1.340 (0.504) | 0.01 | 5210.45 (4545.97) | 0.255 | 4791.80  [1234.00, 10757.52] | 0.014* | 10002.25  (4339.46) | 0.024 | SM | — |
| ALPS_right | SPC2_All | 2.763 (0.935) | 0.004 | -2.861 (0.467) | <0.001 | 10.749 (4.000) | 0.009 | -7.90  [-13.40, -2.80] | 0.015* | 2.84  (4.615) | 0.54 | SM | — |
| CPV | SPC1_All | 1960.52 (447.54) | <0.001 | 1.217 (0.515) | 0.021 | 3390.11 (2237.69) | 0.134 | 2386.07  [631.52, 5048.56] | 0.016* | 5776.18  (2056.85) | 0.006 | CM | 41.3 |
| CPV_right | SPC1_All | 3463.39 (780.96) | <0.001 | 1.180 (0.515) | 0.025 | 6493.95 (3912.81) | 0.101 | 4086.18  [1050.45, 8366.82] | 0.019* | 10580.13  (3580.02) | 0.004 | CM | 38.6 |
| CPV | SPC2_All | -1072.89 (444.86) | 0.018 | -2.228 (0.471) | <0.001 | 3385.64 (1883.43) | 0.076 | 2390.54  [675.26, 4549.05] | 0.031* | 5776.18  (2056.85) | 0.006 | CM | 41.4 |
| ALPS | SPC2_All | 2.855 (1.006) | 0.006 | -2.820 (0.468) | <0.001 | 10.724 (4.293) | 0.015 | -8.05  [-13.93, -2.14] | 0.036* | 2.67  (4.946) | 0.591 | SM | — |
| CPV_left | SPC2_All | -2133.46 (926.84) | 0.024 | -2.297 (0.474) | <0.001 | 5100.64 (3938.70) | 0.199 | 4901.61  [1169.50, 9322.45] | 0.039* | 10002.25  (4339.46) | 0.024 | CM | 49 |
| ALPS_left | SPC4_All | 2.318 (0.627) | <0.001 | -2.474 (0.764) | 0.002 | 7.380 (4.509) | 0.106 | -5.73  [-11.21, -0.87] | 0.041* | 1.65  (4.402) | 0.71 | SM | — |
| CPV_right | SPC2_All | -1769.61 (781.59) | 0.026 | -2.214 (0.466) | <0.001 | 6661.46 (3260.98) | 0.045 | 3918.67  [825.88, 8089.63] | 0.047* | 10580.13  (3580.02) | 0.004 | PM | 37 |

Supplementary Table S35. Significant mediation analysis results among the five SPCs from TBSS differences in the whole cohort (PD+HC), glymphatic system function metrics, and apathy symptoms (outcome variable Y = AES for all models).

*p*, p‑value after FDR correction; *, *pFDR* < 0.05. For suppressive mediation types, the proportion mediated is negative or meaningless, denoted as "—".

AES, Apathy Evaluation Scale; CPV, choroid plexus volume; CPV_left, left hemisphere CPV; CPV_right, right hemisphere CPV; PVS, perivascular space volume; PVS_SUB, PVS in subcortical gray matter nuclei; PVS_WM, PVS in the white matter region; pPVS, whole-brain PVS; ALPS, index of diffusion along perivascular spaces; ALPS_left, left hemisphere ALPS; ALPS_right, right hemisphere ALPS; X, independent variable; M, mediator variable; a path, coefficient of X→M; b path, coefficient of M→Y; c', direct effect (ADE); ab, indirect effect (ACME); c, total effect; SE, standard error; CI, confidence interval; %, proportion mediated (Prop_Mediated). Mediation types: CM, complete mediation; PM, partial mediation; SC, suppressive‑competitive; SM, suppressive‑masked.

| X | M | a path (SE) | a path *p* | b path (SE) | b path *p* | c'(ADE) (SE) | c' *p* | ab (ACME) [95% CI] | ab *pFDR* | c(Total) (SE) | c *p* | Type | % |
| --- | --- | --- | --- | --- | --- | --- | --- | --- | --- | --- | --- | --- | --- |
| SPC3_PD | ALPS | 0.044 (0.019) | 0.024 | 13.277 (6.079) | 0.036 | -3.031 (0.741) | <0.001 | 0.58  [0.02, 1.54] | 0.233 | -2.45 (0.726) | 0.002 | — | — |
| CPV_left | SPC3_PD | -3572.74 (1193.35) | 0.005 | -2.500 (0.820) | 0.004 | -953.60 (6634.91) | 0.836 | 8931.59  [1707.57, 18279.52] | 0.143 | 7977.99 (6586.31) | 0.287 | — | — |
| CPV | SPC3_PD | -1877.94 (547.40) | 0.001 | -2.388 (0.845) | 0.008 | 463.13 (3229.31) | 0.92 | 4485.21  [981.79, 8971.90] | 0.143 | 4948.33 (3067.11) | 0.159 | — | — |
| CPV_right | SPC3_PD | -3264.18 (951.54) | 0.001 | -2.294 (0.843) | 0.01 | 2081.86 (5604.17) | 0.742 | 7487.96  [1466.50, 14954.65] | 0.157 | 9569.82 (5286.66) | 0.123 | — | — |
| ALPS | SPC3_PD | 2.953 (1.258) | 0.024 | -3.031 (0.741) | <0.001 | 13.277 (6.079) | 0.015 | -8.95  [-19.45, -1.41] | 0.218 | 4.33 (6.769) | 0.437 | — | — |
| PVS_WM | SPC3_PD | 22.394 (8.697) | 0.014 | -2.339 (0.798) | 0.006 | -16.04 (45.835) | 0.678 | -52.38  [-118.53, -3.10] | 0.233 | -68.42 (46.338) | 0.082 | — | — |
| ALPS_left | SPC3_PD | 2.447 (1.045) | 0.025 | -2.904 (0.760) | <0.001 | 8.651 (5.176) | 0.039 | -7.11  [-15.80, -0.45] | 0.233 | 1.55 (5.649) | 0.723 | — | — |
| ALPS_right | SPC3_PD | 2.323 (1.281) | 0.078 | -2.907 (0.720) | <0.001 | 13.062 (5.854) | 0.028 | -6.75  [-16.23, -0.14] | 0.233 | 6.31 (6.670) | 0.304 | — | — |

Supplementary Table S36. Significant mediation analysis results among the three SPCs from the PD group, glymphatic system function metrics, and apathy symptoms (outcome variable Y = AES for all models).
After FDR correction, the indirect effects of all pathways did not reach statistical significance (*pFDR* > 0.05). Uncorrected raw p‑values are presented for trend reference, and confidence intervals that do not contain zero suggest a potential effect. *p*, p‑value after FDR correction; *, *pFDR* < 0.05. For suppressive mediation types, the proportion mediated is negative or meaningless, thus denoted as "—". A dash ("—") in the Type column indicates no significant mediation type after rigorous correction.
AES, Apathy Evaluation Scale; CPV, choroid plexus volume; CPV_left, left hemisphere CPV; CPV_right, right hemisphere CPV; PVS, perivascular space volume; PVS_SUB, PVS in subcortical gray matter nuclei; PVS_WM, PVS in the white matter region; pPVS, whole-brain PVS; ALPS, index of diffusion along perivascular spaces; ALPS_left, left hemisphere ALPS; ALPS_right, right hemisphere ALPS; X, independent variable; M, mediator variable; a path, coefficient of X→M; b path, coefficient of M→Y; c', direct effect (ADE); ab, indirect effect (ACME); c, total effect; SE, standard error; CI, confidence interval; %, proportion mediated (Prop_Mediated). Mediation types: CM, complete mediation; PM, partial mediation; SC, suppressive‑competitive; SM, suppressive‑masked.

| X | M | a path (SE) | a path *p* | b path (SE) | b path *p* | c'(ADE) (SE) | c' *p* | ab (ACME) [95% CI] | ab *pFDR* | c(Total) (SE) | c *p* | Type | % |
| --- | --- | --- | --- | --- | --- | --- | --- | --- | --- | --- | --- | --- | --- |
| CPV_left | FWcluster3 | 54.744  (19.780) | 0.009 | 155.975  (49.031) | 0.003 | -560.67  (6481.24) | 0.932 | 8538.66  [1744.61, 16423.79] | 0.232 | 7977.99  (6586.31) | 0.233 | — | — |
| CPV | FWcluster3 | 28.129  (9.168) | 0.004 | 148.697  (49.949) | 0.005 | 765.63  (3119.69) | 0.808 | 4182.70  [826.60, 8594.26] | 0.232 | 4948.33  (3067.11) | 0.115 | — | — |
| CPV_right | FWcluster3 | 48.064  (16.004) | 0.005 | 143.276  (49.606) | 0.007 | 2683.44  (5385.70) | 0.621 | 6886.38  [1238.48, 15004.84] | 0.232 | 9569.82  (5286.66) | 0.078 | — | — |

Supplementary Table S37. Significant mediation analysis results among TBSS differences, glymphatic system function metrics, and apathy symptoms in the APD and nAPD groups (outcome variable Y = AES for all models).

After FDR correction, the indirect effects of all pathways did not reach statistical significance (*pFDR* > 0.05). Uncorrected raw p‑values are presented for trend reference, and confidence intervals that do not contain zero suggest a potential effect. *p*, p‑value after FDR correction; *, *pFDR* < 0.05. For suppressive mediation types, the proportion mediated is negative or meaningless, thus denoted as "—". A dash ("—") in the Type column indicates no significant mediation type after rigorous correction.

Abbreviations: AES, Apathy Evaluation Scale; CPV, choroid plexus volume; CPV_left, left hemisphere CPV; CPV_right, right hemisphere CPV; PVS, perivascular space volume; PVS_SUB, PVS in subcortical gray matter nuclei; PVS_WM, PVS in the white matter region; pPVS, whole-brain PVS; ALPS, index of diffusion along perivascular spaces; ALPS_left, left hemisphere ALPS; ALPS_right, right hemisphere ALPS; X, independent variable; M, mediator variable; a path, coefficient of X→M; b path, coefficient of M→Y; c', direct effect (ADE); ab, indirect effect (ACME); c, total effect; SE, standard error; CI, confidence interval; %, proportion mediated (Prop_Mediated). Mediation types: CM, complete mediation; PM, partial mediation; SC, suppressive‑competitive; SM, suppressive‑masked.
